# Leaf-GP: an open and automated software application for measuring growth phenotypes for arabidopsis and wheat

**DOI:** 10.1186/s13007-017-0266-3

**Published:** 2017-12-22

**Authors:** Ji Zhou, Christopher Applegate, Albor Dobon Alonso, Daniel Reynolds, Simon Orford, Michal Mackiewicz, Simon Griffiths, Steven Penfield, Nick Pullen

**Affiliations:** 1 Earlham Institute, Norwich Research Park, Norwich, UK; 2 John Innes Centre, Norwich Research Park, Norwich, UK; 3 University of East Anglia, Norwich Research Park, Norwich, UK

**Keywords:** Growth phenotypes, Automated trait analysis, Feature extraction, Computer vision, Software engineering, Arabidopsis, Wheat

## Abstract

**Background:**

Plants demonstrate dynamic growth phenotypes that are determined by genetic and environmental factors. Phenotypic analysis of growth features over time is a key approach to understand how plants interact with environmental change as well as respond to different treatments. Although the importance of measuring dynamic growth traits is widely recognised, available open software tools are limited in terms of batch image processing, multiple traits analyses, software usability and cross-referencing results between experiments, making automated phenotypic analysis problematic.

**Results:**

Here, we present Leaf-GP (Growth Phenotypes), an easy-to-use and open software application that can be executed on different computing platforms. To facilitate diverse scientific communities, we provide three software versions, including a graphic user interface (GUI) for personal computer (PC) users, a command-line interface for high-performance computer (HPC) users, and a well-commented interactive *Jupyter Notebook* (also known as the iPython Notebook) for computational biologists and computer scientists. The software is capable of extracting multiple growth traits automatically from large image datasets. We have utilised it in *Arabidopsis thaliana* and wheat (*Triticum aestivum*) growth studies at the Norwich Research Park (NRP, UK). By quantifying a number of growth phenotypes over time, we have identified diverse plant growth patterns between different genotypes under several experimental conditions. As Leaf-GP has been evaluated with noisy image series acquired by different imaging devices (e.g. smartphones and digital cameras) and still produced reliable biological outputs, we therefore believe that our automated analysis workflow and customised computer vision based feature extraction software implementation can facilitate a broader plant research community for their growth and development studies. Furthermore, because we implemented Leaf-GP based on open Python-based computer vision, image analysis and machine learning libraries, we believe that our software not only can contribute to biological research, but also demonstrates how to utilise existing open numeric and scientific libraries (e.g. Scikit-image, OpenCV, SciPy and Scikit-learn) to build sound plant phenomics analytic solutions, in a efficient and effective way.

**Conclusions:**

Leaf-GP is a sophisticated software application that provides three approaches to quantify growth phenotypes from large image series. We demonstrate its usefulness and high accuracy based on two biological applications: (1) the quantification of growth traits for *Arabidopsis* genotypes under two temperature conditions; and (2) measuring wheat growth in the glasshouse over time. The software is easy-to-use and cross-platform, which can be executed on Mac OS, Windows and HPC, with open Python-based scientific libraries preinstalled. Our work presents the advancement of how to integrate computer vision, image analysis, machine learning and software engineering in plant phenomics software implementation. To serve the plant research community, our modulated source code, detailed comments, executables (.exe for Windows; .app for Mac), and experimental results are freely available at https://github.com/Crop-Phenomics-Group/Leaf-GP/releases.

**Electronic supplementary material:**

The online version of this article (10.1186/s13007-017-0266-3) contains supplementary material, which is available to authorized users.

## Background

Plants demonstrate dynamic growth phenotypes that are determined by genetic and environmental factors [1–3]. Phenotypic features such as relative growth rates (RGR), vegetative greenness and other morphological characters are popularly utilised by researchers to quantify how plants interact with environmental changes (i.e. G × E) and different experimental treatments [4–6]. In particular, to assess the growth and development, growth phenotypes such as leaf area, leaf convex hull size and leaf numbers are considered as key measurements by plant scientists [7–12], indicating the importance of scoring differences of growth related traits between experiments. To accomplish the above tasks, high quality image-based growth data need to be collected from many biological replicates over time [13, 14], which is then followed by manual, semi-automated, or automated trait analysis [15, 16]. However, the current bottleneck lies in how to extract meaningful results from the increasing image-based data, effectively and efficiently [14, 17].

To facilitate the quantification of dynamic growth traits, a range of imaging hardware and software have been developed. To demonstrate the development of this research domain, we summarise some representative tools and techniques as follows:

- LeafAnalyser [18] uses image-processing techniques to measure leaf shape variation as well as record the position of each leaf automatically.
- GROWSCREEN [12] quantifies dynamic seedling growth under altered light conditions.
- GROWSCREEN FLUORO [19] measures leaf growth and chlorophyll fluorescence to detect stress tolerance.
- LemnaGrid [20] integrates image analysis and rosette area modelling to assess genotype effects for *Arabidopsis*.
- Leaf Image Analysis Interface (LIMANI) [21] segments and computes venation patterns of *Arabidopsis* leaves.
- Rosette Tracker [22] provides an open Java-based image analysis solution to evaluate plant-shoot phenotypes to facilitate the understanding of *Arabidopsis* genotype effects.
- PhenoPhyte [23] semi-automates the quantification of various 2D leaf traits through a web-based software application.
- Depth imaging systems were used to measure 3D leaf areas using a segmentation algorithm, so that plants can be phenotyped from a top-view perspective [24].
- OSCILLATOR [25] analyses rhythmic leaf growth movement using infrared photography combined with wavelet transformation in mature plants.
- HPGA (a high-throughput phenotyping platform for plant growth modelling and functional analysis) [5], which produces plant area estimation and growth modelling and analysis to high-throughput plant growth analysis.
- LeafJ [26] provides an ImageJ plugin to semi-automate leaf shape measurement.
- Integrated Analysis Platform (IAP) [16] is an open framework that performs high-throughput plant phenotyping based on the LemnaTec system.
- Low-cost 3D systems such as Microsoft Kinect and the David laser scanning system are evaluated for their potential applications in plant phenotyping [27].
- Easy Leaf Area [28] uses colour-based feature to differentiate and measure leaves from their background using a red calibration area to replace scale measurement.
- Phytotyping^4D^ [29] employs a light-field camera to simultaneously provide a focus and a depth image so that distance information from leaf surface can be quantified.
- Large-scale gantry system, LeasyScan [30], is able to assess canopy traits affecting water use based on leaf area, leaf area index and transpiration. The system is based on 3D laser scanning techniques and Phenospex’s proprietary software to conduct 3D trait measurements.
- Leaf Angle Distribution Toolbox [31] is a Matlab-based software package for quantifying leaf surface properties via 3D reconstruction from stereo images.
- MorphoLeaf [32] is a plug-in for the Free-D software to perform analysis of morphological features of leaves with different architectures.
- rosettR [33] is a high-throughput phenotyping protocol for measuring total rosette area of seedlings grown in plates.
- A real-time machine learning based classification phenotyping framework [34] can extract leaf canopy to rate soybean stress severity.
- Phenotiki [35] is an affordable system for plant phenotyping, integrating off-the-shelf hardware components and easy-to-use Matlab-based software for phenotyping rosette-shaped plants.

While many hardware and software solutions have been created, the threshold for using these existing tools for measuring growth phenotypes is still relatively high. This is due to many analytic software solutions that are either customised for specific hardware platforms (e.g. LemnaTec Scanalyzer and Phenospex PlantEye), or relied on proprietary (LemnaTec HTS Bonit) or specialised software platforms (e.g. Matlab), restricting smaller or not well-funded laboratories to access the existing solutions [22]. Hence, data annotation, phenotypic analysis, and results cross-referencing are still frequently done manually in many laboratories, which is time consuming and prone to errors [21].

Available open software tools are also limited in terms of batch processing, multiple trait analysis, and software usability, making automated phenotypic analysis problematic [33]. In order to provide a fully open analytics software framework for a broader plant research community to measure key growth-related phenotypes, we developed Leaf-GP (Growth Phenotypes), an open-source and easy-to-use software solution that can be easily setup for analysing images captured by low-cost imaging devices. The software uses the community driven Python-based scientific and numeric libraries. After continuous development and testing, Leaf-GP can now extract and compare growth phenotypes reliably from large image series, including projected leaf area (mm^2^), leaf perimeter (mm), leaf convex hull length and width (mm), leaf convex hull area (mm^2^), stockiness (%), compactness (%), leaf numbers and greenness (0–255). We demonstrate its high accuracy and usefulness through experiments using *Arabidopsis thaliana* and *Paragon* wheat (a UK spring wheat variety). The software can be executed on mainstream operating systems with Python and Anaconda distribution preinstalled. More importantly, we followed the open software design strategy, which means that our work is expandable and new functions or modules can be easily added to the software framework.

## Methods

### Applying Leaf-GP to plant growth studies

**Fig. 1:**
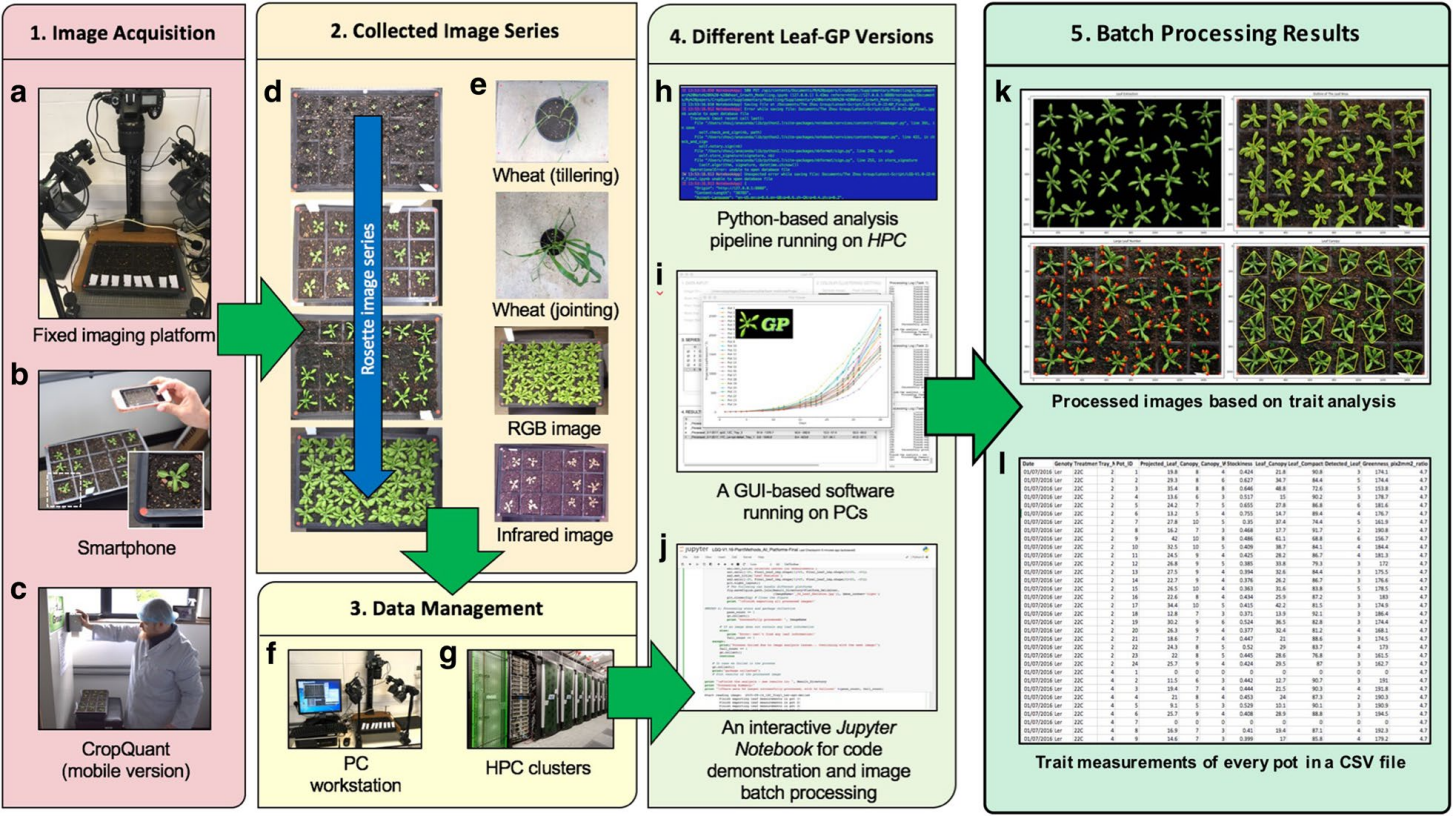
An overview of how to utilise Leaf-GP in plant growth research. **a**–**c** A range of imaging devices, including a fixed imaging platform, smartphones, or a mobile version CropQuant equipped with either a *Pi* NoIR sensor or an RGB sensor. **d**, **e** The regions of a tray or a pot need to be captured. **f**, **g** Both raw and processed image data can be loaded and saved by Leaf-GP on PCs, HPC clusters, or cloud-based computing storage. **h**, **j** Three versions of Leaf-GP, including HPC, GUI and a *Jupyter Notebook*. **k**, **l** Processed images highlighting key growth phenotypes and CSV files containing trait measurements are produced after the batch image processing

Figure 1 illustrates how Leaf-GP was applied to quantify growth phenotypes for *Arabidopsis* rosettes and *Paragon* wheat over time. To improve the software flexibility, Leaf-GP was designed to accept both RGB (a red, green and blue colour model) and infrared (sensitive to short-wavelength infrared radiation at around 880 nm) images acquired by a range of low-cost devices, including a fixed imaging platform using a Nikon D90 digital camera (Fig. 1a), smartphones (e.g. an iPhone, Fig. 1b), or a mobile version CropQuant [36] equipped with either a *Pi* NoIR (no infrared filter) sensor or an RGB sensor (Fig. 1c). When taking pictures, users need to ensure that the camera covers the regions of interest (ROI), i.e. a whole tray (Fig. 1d) or a pot region (Fig. 1e). Red circular stickers (4 mm in radius in our case) need to be applied to the four corners of a tray or a pot (Fig. 1b). In doing so, Leaf-GP can extract ROI from a given image based on red markers’ positions and then convert measurements from pixels to metric units (i.e. millimetre, mm) using the diameter of the marker as the scale of the image. Both raw and processed image data can be loaded and saved by Leaf-GP on personal computers (PCs), HPC, or cloud-based computing storage (Figs. 1f, g).

As different research groups may have access to dissimilar computing infrastructures, we developed three versions of Leaf-GP to enhance the accessibility of the software: (1) for users utilising HPC clusters, a Python-based script was developed to perform high-throughput trait analysis through a command-line interface (Fig. 1h), which requires relevant scientific and numeric libraries such as SciPy [37], computer vision (i.e. the Scikit-image library [38] and the OpenCV library [39]), and machine learning libraries (i.e. the Scikit-learn library [40]) pre-installed on the clusters; (2) for users working on desktop PCs, a GUI-based software version was developed to incorporate batch image processing, multi-traits analyses, and results visualisation (in CSV format, comma-separated values) in a user-friendly window (Fig. 1i); and (3) for computational biologists and computer scientists who are willing to exploit our source code, we created an interactive *Jupyter Notebook* (Fig. 1j, see Additional file 1) to explain the trait analysis workflow as well as the software implementation. In particular, we have enable the *Notebook* version to process large image series via a *Jupyter* server, which means users can carry out stepwise algorithm execution and/or batch processing images directly using the Notebook version. Due to software distribution licensing issues, we recommend users to install the Anaconda Python distribution (Python 2.7 version) and OpenCV (v2.4.11) libraries before using Leaf-GP. We used PyInstaller [41] to package Leaf-GP. Additional file 2 explains the step-by-step procedure of how to install Python and necessary libraries for our software.

**Fig. 2:**
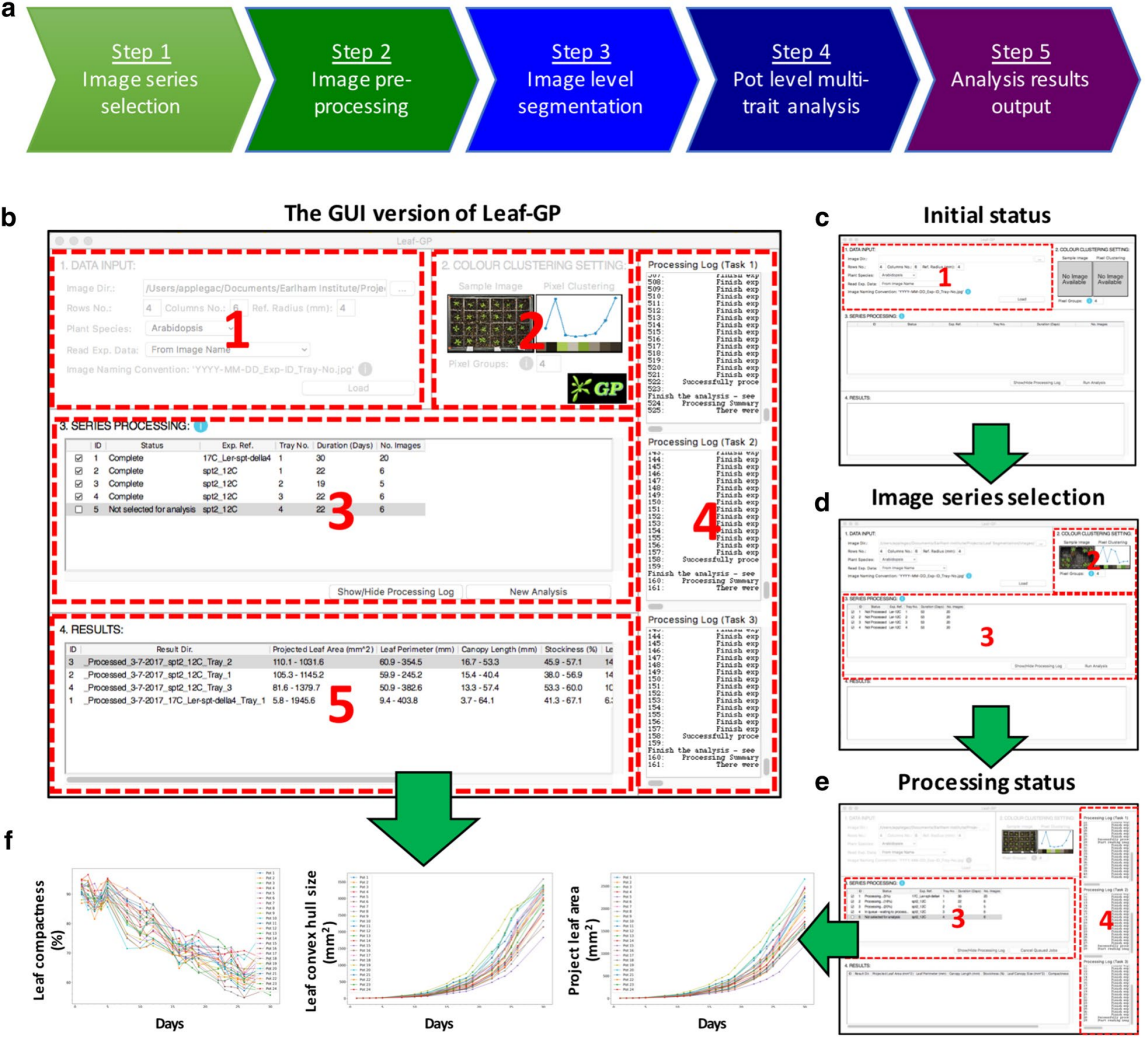
The analysis workflow and the GUI of Leaf-GP. **a** The high-level analysis workflow of Leaf-GP, containing five main steps. **b** Five self-explanatory sections designed to integrate the analysis workflow into the GUI version of the software. **c** The initial status of the GUI. **d** The screenshot after selecting image series. **e** The screenshot when image datasets are being processed in parallel computing. **f** Growth-related trait plots can be generated based on the result CSV file, by clicking the associated cell in the Results table

After trait analysis, two types of output results are generated. First, *processed images* (Fig. 1k), which includes pre-processing results, calibrated images, colour clustering, and figures exhibiting key growth traits such as leaf outlines, leaf skeletons, detected leaves, and leaf convex hull (Additional file 3). Second, *comprehensive CSV files* that follow the open ISA framework [42] and the PlantOmics [43] naming convention (Fig. 1l), containing image name, experimental data, pot ID, pixel-to-mm ratio, and biologically relevant outputs including projected leaf area (mm^2^), leaf perimeter, convex hull length and width (in mm), stockiness (%), leaf convex hull size (mm^2^), leaf compactness (%), the number of detected leaves, and greenness (Additional file 4). These CSV files were produced with experimental metadata and phenotypic data, so that they can be indexed on the clusters or searched on PCs by experiments or treatments. Also, Leaf-GP can visualise each CSV file automatically, within the software framework (Fig. 2). The source code used to plot and compare growth phenotypes is provided in Additional file 5, called Leaf-GP plot generator.

### The GUI of Leaf-GP

As plant researchers commonly use PCs for their analyses, we specifically develop the Leaf-GP GUI version using Python’s native GUI package, Tkinter [44]. The GUI version can operate on different platforms (e.g. Windows and Mac OS) and the default resolution of the main window is set to 1024 × 768 pixels, so that it can be compatible with earlier operating systems (OS) such as Windows Vista. Figure 2 illustrates how to use the GUI window to process multiple growth image series. A high-level analysis workflow of Leaf-GP is presented in Fig. 2a, containing five steps: (1) data selection (2) image pre-processing (3) global ROI segmentation (i.e. at image level), (4) local trait analysis (i.e. at the pot level), and (5) results output. To explain the analysis workflow, we also prepared a detailed UML (unified modelling language) activity diagram [45] that elucidates stepwise actions in Additional file 6, which includes software engineering activities such as choice, iteration, and concurrency to enable the batch processing of large image datasets.

Figure 2b shows five self-explanatory sections designed to integrate the above analysis workflow into the GUI version, including: Data Input, Colour Clustering Setting, Series Processing, Processing Log (a hidden section), and Results Section. To analyse one or multiple image series, users need to follow these sections sequentially. A number of information icons (coloured blue) have been included to explain how to enter input parameters.

#### Section 1—data input

To simplify the data input phase, we only require users to enter essential information regarding their images and associated experiments. To complete the section (Fig. 2c), a user first needs to choose a directory (“Image Dir.”) which contains captured image series. The GUI version can accept both JPEG and PNG files (see Step 4.1 in Additional file 1), with resolutions ranged from 4288  ×  2848 (5–7 MB) to 2592  ×  1944 (3–5 MB). Then, the user shall enter parameters in the “Row No.” and “Column No.” input boxes to define the layout of the tray used in the experiment as well as “Ref. Radius (mm)” to specify the radius of the red stickers. Finally, the user needs to select from “Plant Species” and “Read Exp. Data” dropdowns. All inputs will be verified upon entry to ensure only valid parameters can be submitted to the core algorithm.

In particular, the “Read Exp. Data” dropdown determines how Leaf-GP reads experiment metadata such as imaging date, treatments and genotypes. For example, choosing the “From Image Name” option allows the software to read information from the filename, selecting the “From Folder Name” option will extract metadata from the directory name, whereas the “No Metadata Available” selection will group all images as an arbitrary series for trait analysis. This option allows users to analyse images that are *not* following any data annotation protocols. Although not compulsory, we developed a simple naming convention protocol (Additional file 7) to assist users to speedily annotate their images or folder names for Leaf-GP.

#### Section 2—colour clustering setting

Once the data input phase is completed, the user can click the ‘Load’ button to initiate series sorting, which will populate the *Colour Clustering Setting* section automatically (Fig. 2d). A sample image from the midpoint of a given series (e.g. in a 10-image series, the 5th image is treated as the midpoint) will be chosen by the software. The midpoint image normally contains representative colour groups during experiment. The image is then processed by a simple k-means method [40], producing a colour clustering plot and a *k* value that represents the number of representative colour groups detected by the k-means method. The *k* value is then populated in the “Pixel Groups” input box. The user can override the *k* value; however, to reduce the computational complexity, Leaf-GP only accepts a maximum value of 10 (i.e. 10 colour groups) and a minimum value of 3 (i.e. three colour groups). The generated *k* value is passed to the core analysis algorithm when analysing growth phenotypes.

#### Sections 3, 4—series processing

In the *Series Processing* section (Fig. 2e), the software fills the processing table with experimental metadata that can help users identify different experiments, including experiment reference (“Exp. Ref.”), the tray number (“Tray No.”), and the number of images in a series (“No. Images”). To improve the appearance of the table, each column is resizable. Checkboxes are prepended to each recognised series (see Additional file 7). Users can toggle one or multiple checkboxes to specify how many experiments will be processed simultaneously. If the ‘No Metadata Available’ option is selected (see the “Data input” section), information such as “Exp. Ref.” and “Tray No.” will not be populated.

The initial status of each processing task (“Status”) is *Not Processed*, which will be updated constantly during the image analysis. When more than one experiment is selected, Python’s thread pool executor function will be applied, so that these experiments can be analysed simultaneously in multiple cores in the central processing unit (CPU). We have limited up to *three* analysis threads (section 4 in Fig. 2e), because many Intel processors comprise *four* physical cores and conducting parallel computing can have a high demand of computing resources (e.g. CPU and memory), particularly when raw image datasets are big.

Once the processing table is filled, the user can click the ‘Run Analysis’ button to commence the analysis. Section 5 (Fig. 2b) shows the screenshot when five experiments (i.e. five series) are recognised and four of them have been analysed. Due to the multi-task design of Leaf-GP, the ‘Status’ column will be continually updated for each series, indicating how many images have been processed in the series. It is important to note that, although Leaf-GP was designed for parallel computing, some functions used in the core algorithm are not thread-safe and hence cannot be executed by multiple threads at a time. Because of this limit, we have utilised lock synchronisation mechanisms to protect certain code blocks (i.e. modules or functions), so that these thread-unsafe blocks can only be executed by one thread at a time. In addition to the processing status, more analysis information and processing log data can be viewed by opening the *Processing Log* section (section 4 in Fig. 2e), which can be displayed or hidden by clicking the ‘Show/Hide Processing Log’ button on the main window.

#### Section 5—Results

When all processing tasks are completed, summary information will be appended to the Results section, including processing ID and a link to the result folder which contains a result CSV file and all processed images (“Result Dir.”). Depending on which species (i.e. *Arabidopsis* rosette or wheat) is selected, trait plots will be generated based on the result CSV file, showing key growth phenotype plots (e.g. the projected leaf area, leaf perimeter, leaf convex hull, leaf compactness, and leaf numbers) by clicking on the associated trait cell in the Results table (Fig. 2f). The range of the measurement is also listed in the *Results* section. The GUI version saves processing statistics, for example, how many images have been successfully analysed and how many images have been declined, together with related error or warning messages in a log file for debugging purposes.

### Core trait analysis algorithms

Multiple trait analysis of *Arabidopsis* rosettes and wheat plants is the core part of Leaf-GP. Not only does it utilise a range of computer vision algorithms for automated trait analysis, it also encapsulates feature extraction methods to produce measures that are biologically relevant to growth phenotypes. In the following sections, we explain the algorithms and related software implementation in detail.

#### Step 2—Pre-processing and calibration

**Fig. 3:**
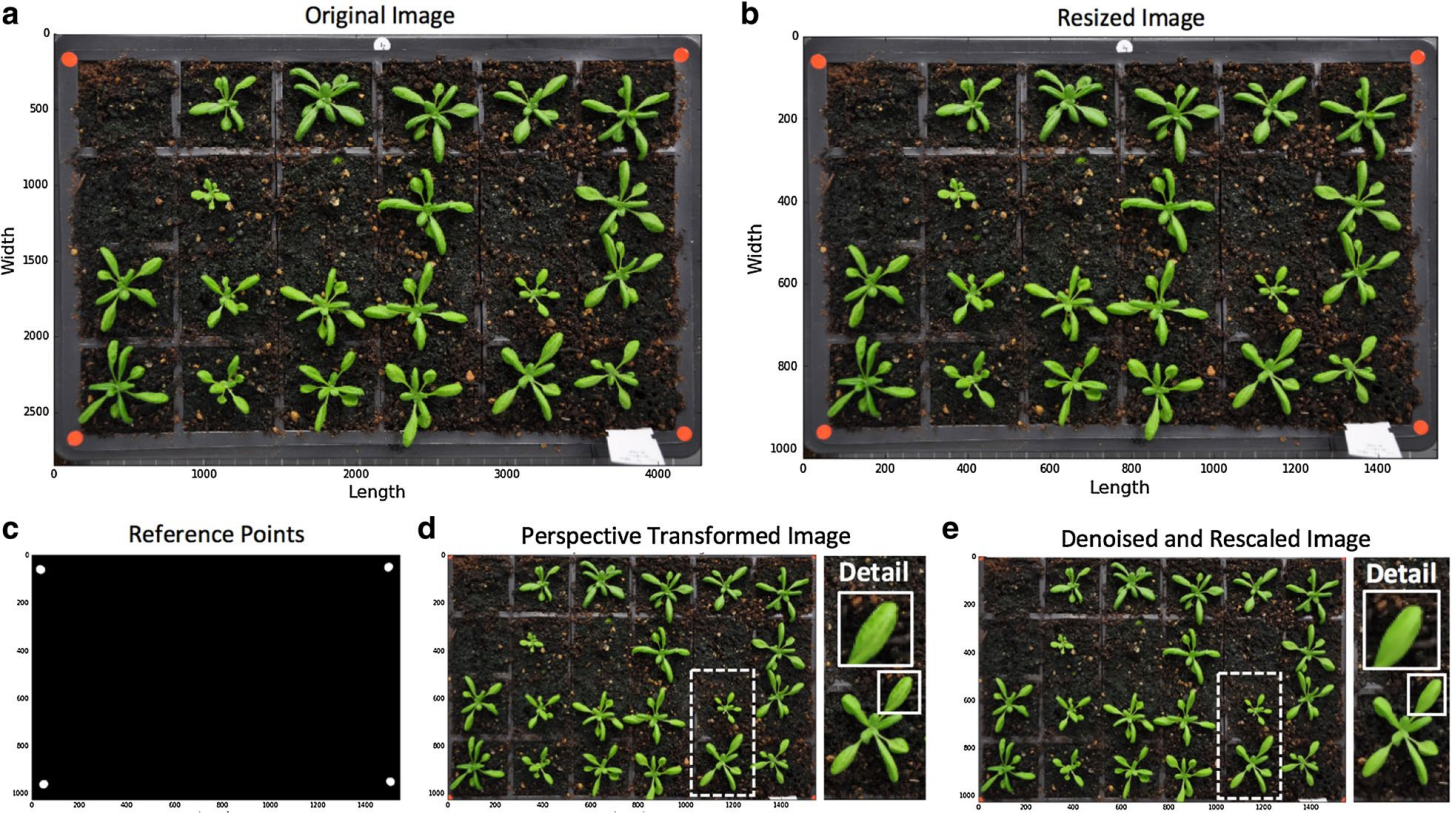
Steps of image pre-processing and calibration. **a**, **b** Fix the height (i.e. y-axis) of all processed images in a given series to reduce computational complexity, raw images are not affected. **c** Detect red circular markers as the image scale. **d** Extract ROI from the original image based on red markers’ positions. **e** Denoise the image to smooth leaf surface for the global leaf segmentation

Different imaging devices, camera positions and even lighting conditions can cause quality variance during image acquisition. Hence, it is important to calibrate images before conducting automated trait analysis. We developed a pre-processing and calibration procedure as shown in Fig. 3. To control memory usage during the batch processing, we first resized each image (Fig. 3a) to a fixed resolution so that the height (i.e. y-axis) of all images in a given series could be fixed. A **rescale** function in Scikit-image was used to dynamically transform the image height to 1024 pixels (Fig. 3b). This resizing approach only modifies the processed image object and hence will not sacrifice potential user power as the raw image is not affected. After that, we created a **RefPoints** function (*Function_2* in Additional file 1) to detect red circular markers attached to the corners of a tray or a pot region. To extract these markers robustly under different illumination conditions, we designed $g\left( {x,y} \right)$, a multi-thresholding function to segment red objects derived from a single-colour extraction approach [46]. The function defines which pixels shall be retained (intensity is set to 1) and which pixels shall be discarded (intensity is set to 0) after the thresholding:

1 $$ g\left( {x,y} \right) = \left\{ {\begin{array}{*{20}l} {1,} &if\; {f_{R} \left( {x,y} \right) > 125\; and\; f_{B} \left( {x,y} \right) < 225\; and\; (f_{R} \left( {x,y} \right) - f_{G} \left( {x,y} \right)) > 50} \\ {0,} & {otherwise} \\ \end{array} } \right. $$

where $f_{R} \left( {x,y} \right)$ is the red channel of a colour image, $f_{B} \left( {x,y} \right)$ represents the blue channel and $f_{G} \left( {x,y} \right)$ the green channel. The result of the function is saved in a reference binary mask.

We then used the **regionprops** function in Scikit-image to measure morphological features of the reference-point mask to filter out false positive items. For example, if there are red-coloured objects on the image, they will be detected by the RefPoints function. However, as their area, eccentricity or solidity readings will *not* fit into the characteristics of a red circular marker, these objects will be discarded during the feature selection. After this step, only genuine circular reference markers are retained (Fig. 3c) and the average radius (in pixels) of the markers is converted to mm units (the radius of the red markers is 4 mm). Using the positions of these markers, we developed a tailored algorithm called **PerspectiveTrans_2D** (*Function_5* in Additional file 1) to extract the tray region, which includes using **getPerspectiveTransform** and **warpPerspective** functions in OpenCV to retain the region that is enclosed by the red markers (Fig. 3d). Finally, we employed a non-local means denoising function called **fastNlMeansDenoisingColored** in OpenCV to smooth leaf surface for the following global leaf ROI segmentation (Fig. 3e).

#### Step 3—Global leaf ROI segmentation

**Fig. 4:**
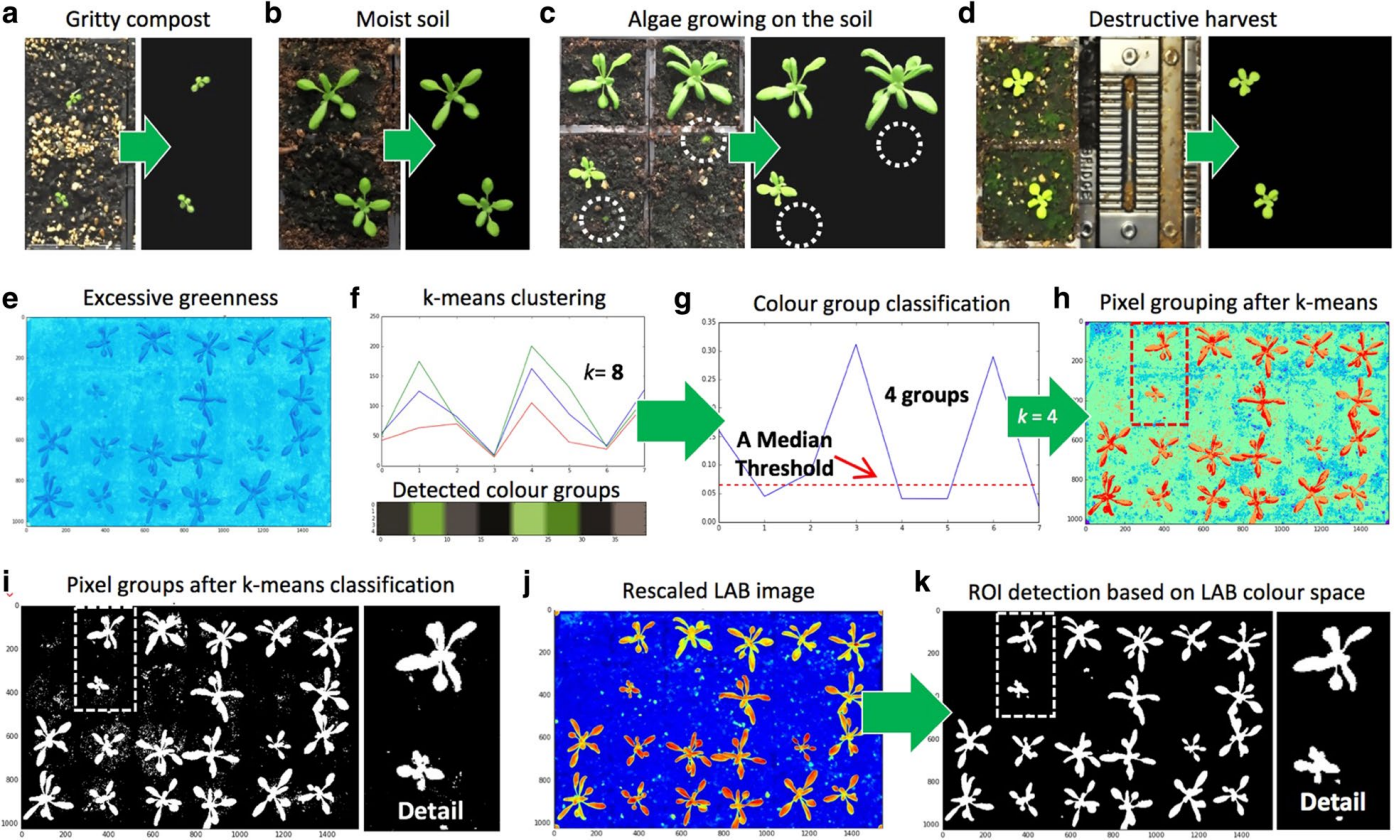
Steps of defining global leaf ROI. **a**–**d** A number of experiment-related problems encountered whilst developing Leaf-GP (to the left of the figures) and results of our solutions (to the right of figures). **e** A pseudo vegetative greenness image generated. **f**, **g** Using k-means to estimate how many colour groups can be classified from a given image. **h** The classification result of the k-means approach based on the pseudo vegetative greenness picture, highlighting green pixels in red. **i** A global adaptive Otsu thresholding used to generate a global leaf ROI binary mask. **j**, **k** A LAB colour space approach used to extract leaf ROI objects at the image level to improve the global leaf ROI result

Besides imaging related issues, changeable experimental settings could also cause problems for automated trait analysis. Figure 4a–d illustrate a number of problems we had encountered whilst developing Leaf-GP. For example, the colour and texture of the soil surface can change considerably between different experiments, especially when gritty compost and other soil types are used (Fig. 4a, b); sometimes plants are *not* positioned in the centre of a pot (Fig. 4b), indicating leaves that cross over to adjacent pots should be segmented; algae growing on the soil has caused false detection due to their bright green colour (Fig. 4c, d); finally, destructive harvest for weighing biomass could occur from time to time throughout an experiment, indicating the core analysis algorithm needs to handle random pot disruption robustly (Fig. 4d). To address the above technical challenges, we developed a number of computer vision and simple machine-learning algorithms based on open scientific libraries. Detection results of our software solutions can be seen to the right of Fig. 4a–d.

The first approach we developed is to establish a consistent approach to extract pixels containing high values of greenness (i.e. leaf regions) from an RGB image robustly. Using a calibrated image, we computed vegetative greenness $G_{V} \left( {x,y} \right)$ [13] based on excessive greenness $Ex_{G} \left( {x,y} \right)$ and excessive red $Ex_{R} \left( {x,y} \right)$ indices [47]. The pseudo vegetative greenness image ($G_{V}$, Fig. 4e) is produced by Eq. 2, based on which we implemented a **compute_greenness_img** function (*Function_8* in Additional file 1) to transfer an RGB image into a $G_{V}$ picture. Excessive greenness is defined by Eq. 3 and excessive red is defined by Eq. 4:

2 $$ G_{V} \left( {x,y} \right) = Ex_{G} \left( {x,y} \right) - Ex_{R} \left( {x,y} \right) $$

3 $$ Ex_{G} \left( {x,y} \right) = 2*f_{G} \left( {x,y} \right) - f_{R} \left( {x,y} \right) - f_{B} \left( {x,y} \right) $$

4 $$ Ex_{R} \left( {x,y} \right) = 1.4*f_{R} \left( {x,y} \right) - f_{B} \left( {x,y} \right) $$

where $f_{R} \left( {x,y} \right)$ is the red channel of a colour image, $f_{B} \left( {x,y} \right)$ represents the blue channel, and $f_{G} \left( {x,y} \right)$ the green channel.

After that, we applied a simple unsupervised machine learning algorithm **KMeans** (default *k* = 8 was used, assuming 8 representative colour groups in a given image) and **KMeans.fit** in Scikit-learn to estimate how many colour groups can be classified (Fig. 4f, *Function_8.1* in Additional file 1). We used a median threshold (red dotted line) to classify the colour groups and obtained the *k* value (Fig. 4g). Also, this process has been integrated into the GUI version (i.e. the *Colour Clustering Setting* section), as descried previously. Utilising the computed *k* value (e.g. *k* = 4, Fig. 4g), we designed a **kmeans_cluster** function (*Function_9* in Additional file 1) to classify the pseudo vegetative greenness picture, highlighting greenness values in red (Fig. 4h). A global adaptive Otsu thresholding [48] was used to generate an image level leaf ROI binary mask (Fig. 4i). After integrating the k-means approach into global ROI segmentation step, we can also provide a sound detection of pot regions that have been destructively harvested, because the colour groups of the harvested pots are often different from the leaf and soil regions. However, it is noticeable that the simple machine learning approach could produce many miss-detected leaf objects due to complicated colour presentations during plant growth experiments (e.g. Fig. 4a–d). For example, the k-means approach performed well when the size of the plants is between 25 and 75% of the size of a pot, but created many false detections when leaves are tiny or the soil background is too complicated. Hence, we designed another approach to improve the leaf detection based on the result of the k-means approach.

We employed Lab colour space [49], which incorporates lightness and green–red colour opponents to refine the detection. We created an internal procedure called **LAB_Img_Segmentation** (*Function_7* in Additional file 1) to transfer RGB images into Lab images using the **color.rgb2lab** function in Scikit-image, based on which green pixels were featured in a non-linear fashion (Fig. 4j). Again, a global adaptive Otsu thresholding was applied to extract leaf objects and then a Lab-based leaf region mask (Fig. 4k). However, the Lab-based approach alone cannot handle destructively harvested pots soundly. As a result, we decided to combine the Lab-based mask with the k-means mask as the output of the phase of global leaf ROI segmentation.

#### Step 4.1—Pot level segmentation

**Fig. 5:**
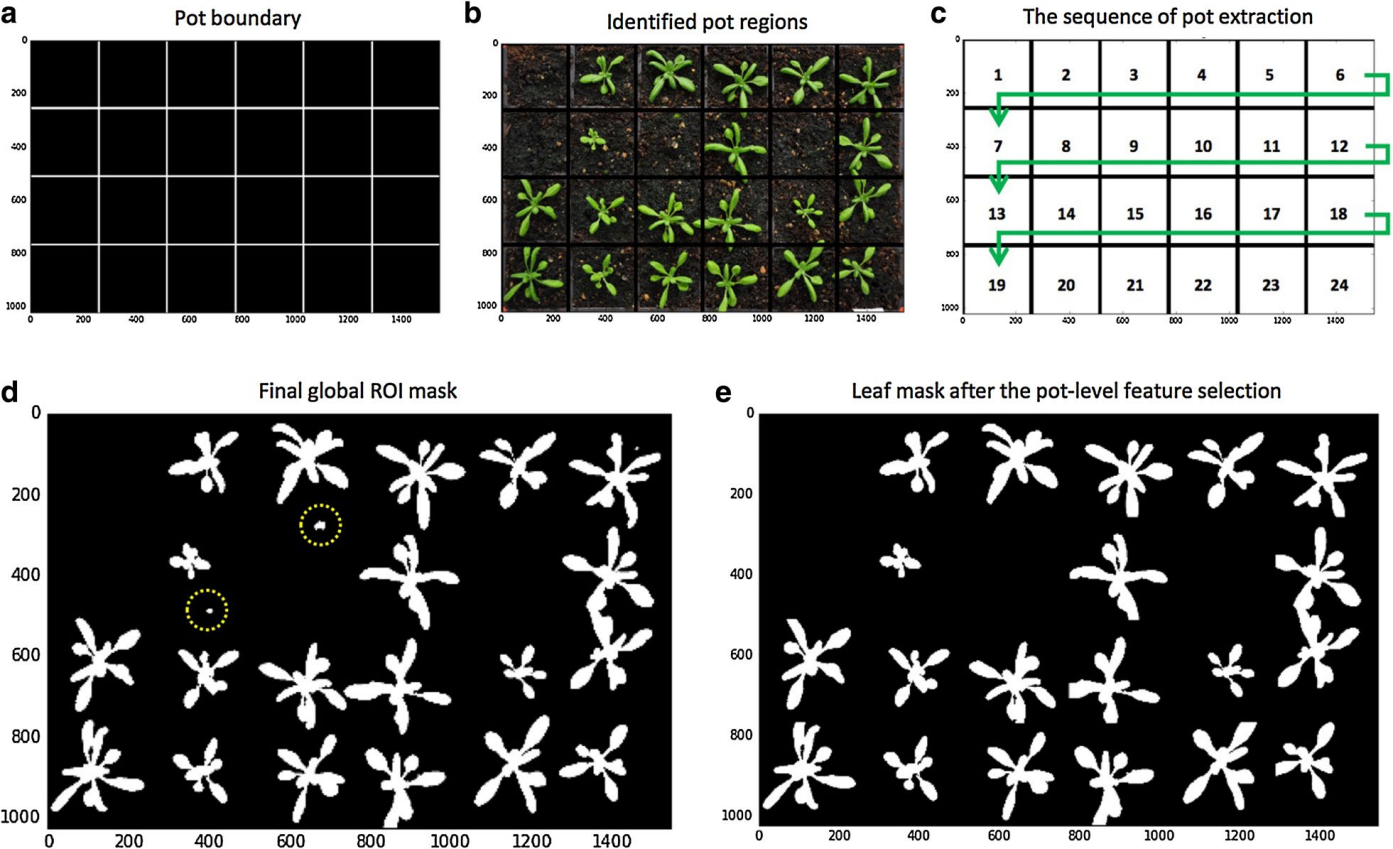
Steps of conducting pot level segmentation in a sequential manner. **a** Depending on the number of rows and columns entered before, anti-aliased lines are generated to define the pot layout. **b** Segmented a given image into a number of sub-images. **c** The sequence of going through each pot. **d**, **e** A local detection method is applied to improve leaf detection

To measure growth phenotypes in a given pot over time, plants within each pot need to be monitored over time. Using the calibrated images, we have defined the tray region, based on which we constructed the pot framework in the tray. To accomplish this task, we designed an iterative layout drawing method called **PotSegmentation** (*Function_5* in Additional file 1) to generate anti-aliased lines using the **line_aa** function in Scikit-image to define the pot layout (Fig. 5a). After constructing the framework, we segmented the whole leaf image into a number of sub-images (Fig. 5b), so that plant can be analysed locally, i.e. at the pot level. Again, we developed an iterative analysis approach to go through each pot with the sequence presented in Fig. 5c.

Within each pot, we conducted a local leaf ROI detection method. For example, by combining leaf masks produced by the machine learning (Fig. 4i) and the Lab colour space (Fig. 4k) approaches, some false positive objects may still remain (Fig. 5d). The local leaf detection can therefore enable us to use pot-level contrast and intensity distribution [50], weighted image moments [51], texture descriptor [52], and leaf positional information to examine each sub-image to refine the leaf detection (Fig. 5e, Step_4.4.2 in Additional file 1). This local feature selection method (detailed in the following sections) can also help us decrease the computational complexity (i.e. memory and computing time) during the batch image processing, as detailed analysis is now carried out within smaller sub-images.

#### Step 4.2—Local multiple trait measurements

**Fig. 6:**
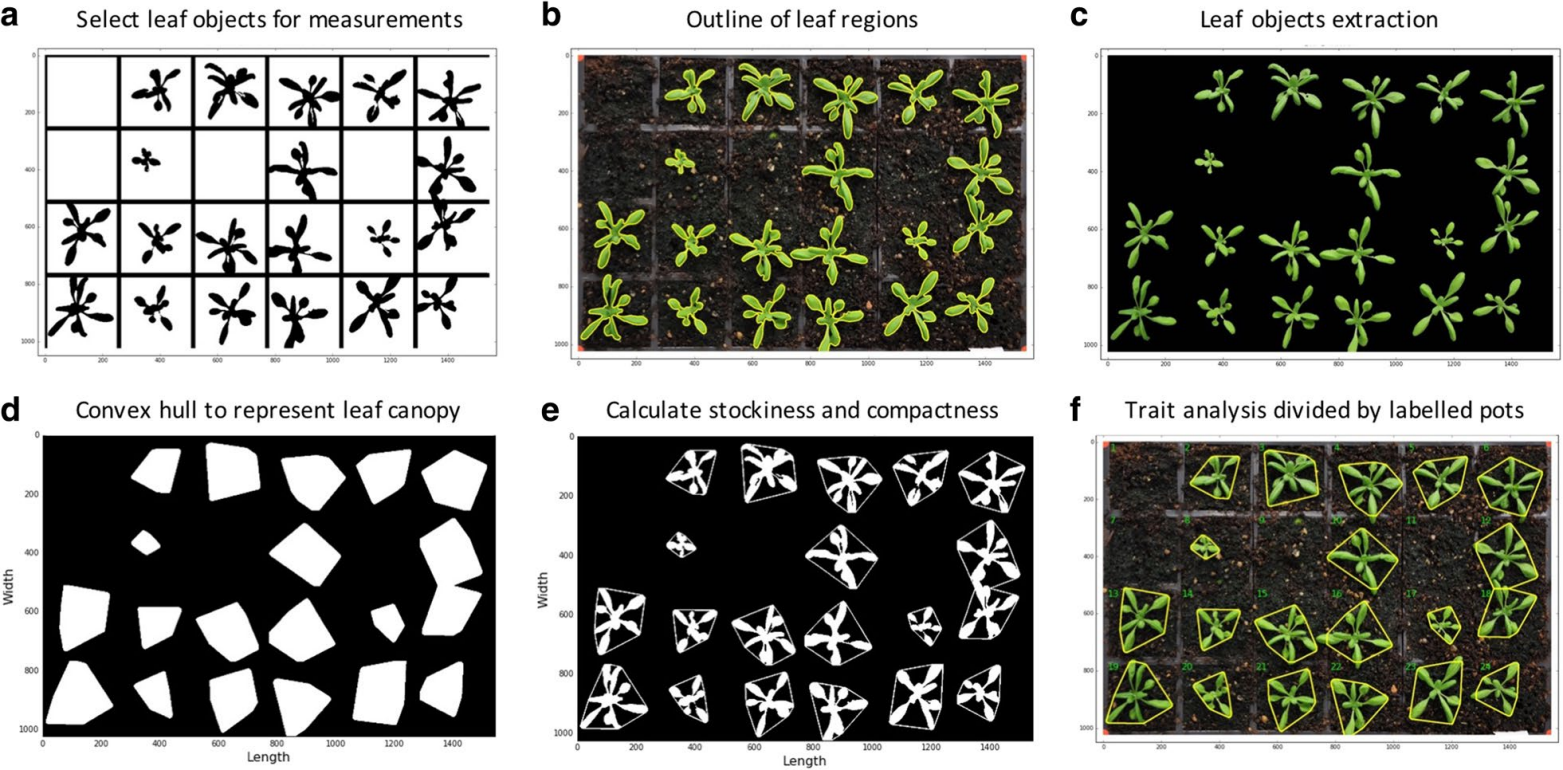
Steps of measuring multiple growth traits. **a** Refined leaf masks for every pot. **b** Contours generated to outline the leaf region. **c** Green pixels enclosed by the contours are totalled for computing the size of the projected leaf area. **d** Convex hulls created in every pot. **e** Stockiness and compactness calculated based on the ratio between the plant projected area and the leaf perimeter. **f** Trait analyses are divided by each pot

Utilising the pot-level leaf masks (Fig. 6a), a number of growth phenotypes could be quantified reliably (Steps_4.4.2 and 4.4.3 in Additional file 1). They are enumerated briefly as follows:

1. “Projected Leaf Area (mm^2^)” measures the area of an overhead projection of the plant in a pot. While implementing the function, the **find_contours** function in Scikit-image is used to outline the leaf region (coloured yellow in Fig. 6b). Green pixels enclosed by the yellow contours are totalled to compute the size of the projected leaf area (Fig. 6c). Pixel-based quantification is then converted to mm units based on the pixel-to-mm exchange rate computed using the reference markers. This trait is a very reliable approximation of the leaf area and has been used in many plant growth studies [20, 22, 53].
2. “Leaf Perimeter (mm)” is calculated based on the length of the yellow contour line that encloses the detected leaf region. Again, pixel-based measurements are converted to mm units, which are then used to compute the size change of a plant over time.
3. “Daily Relative Growth Rate (%)” (Daily RGR) quantifies the speed of plant growth. Derived from the RGR trait described previously [19, 54], the Daily RGR here is defined by Eq. 5:
  5 $$ \frac{1}{{\left( {t2 - t1} \right)}}*\left( {\ln (Area2_{i} } \right) - { \ln }(Area1_{i} )/{ \ln }(Area1_{i} ) $$
  where ${ \ln }$ is natural logarithm, $Area1_{i}$ is the projected leaf area in pot *i* in the previous image, $Area2_{i}$ is the leaf area in pot *i* in the current image, and $\left( {t2 - t1} \right)$ is the duration (in days) between the two consecutive images.
4. “Leaf Convex Hull (mm^2^)” expresses the extracted leaf region that is enclosed by a 2D convex hull in a pot [19, 20, 22]. The convex hull was generated using the **convex_hull_image** function in Scikit-image, enveloping all pixels that belong to the plant with a convex polygon [55]. Figure 6d presents all convex hulls created in a given tray. As described previously [19], this trait can be used to define the coverage of the leaf region as well as how the petiole length changes during the growth.
5. “Stockiness (%)” is calculated based on the ratio between the leaf projected area and the leaf perimeter [22, 56]. It is defined as $(4\pi *Area_{i} )/\left( {2\pi *R_{i} } \right)^{2}$, where $Area_{i}$ is the projected leaf area detected in pot *i* and $R_{i}$ is the longest radius (i.e. major axis divided by 2) of the convex hull polygon in pot *i* (Fig. 6e). This trait (0–100%) has been used to measure how serrated a plant is, which can also indicate the circularity of the leaf region (e.g. a perfect circle will score 100%).
6. “Leaf Compactness (%)” is computed based on the ratio between the projected leaf area and the area of the convex hull enclosing the plant [20, 22]. Figure 6f shows how green leaves are enclosed by yellow convex hull outlines that calculates the leaf compactness trait.
7. “Greenness” monitors the normalised greenness value (0–255) within the convex hull region. As described before, we used the **compute_greenness_img** function to provide the greenness reading, so that we could minimise the background noise caused by algae and soil types. Greenness can be used to study plant growth stages such as vegetation and flowering [16].

### Step 4.3—Leaf number detection

As the number of rosette leaves and the leaf size are popularly used to determine key growth stages for *Arabidopsis* [15], we therefore designed a leaf structure detection algorithm to explore how to provide a consistent reading of traits such as the number of detected leaves and the number of large leaves over time. This algorithm comprises of a 2D skeletonisation algorithm (*Function_10* in Additional file 1) and an outline sweeping method (*Function_11* in Additional file 1).

**Fig. 7:**
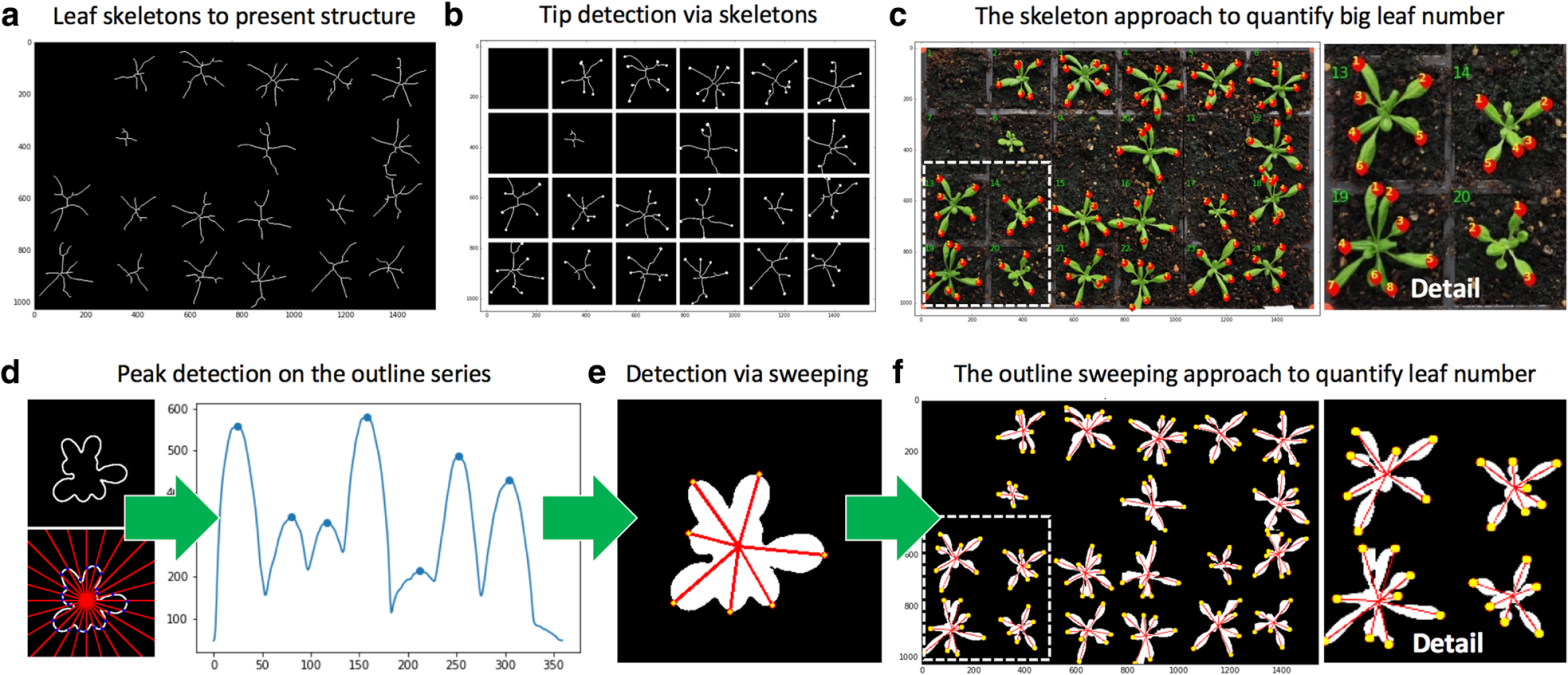
Steps of detecting leaf structure. **a** The result of a 2D skeletonisation approach to extract leaf structure. **b** Detect end points of the leaf structure that correlate with leaf tips. **c** Large or long rosette leaves identified if they are between 50 and 70% of the final size. **d** Generate a leaf outline series to represent the distance between the plant centroid and its leaf contour, at angles between 0 and 359 degrees with a 15-degree interval. **e**, **f** The number of detected peaks are used to represent the number of leaf tips

Figure 7a demonstrates the result of the skeletonisation approach, which utilises the **skeletonize** function in Scikit-image to extract 2D skeletons from the leaf masks within each pot. The skeletons can be used to quantify the structural characteristics of a plant [57]. In our case, we use the approach to measure the number of leaf tips and branching points of a rosette leaf. For example, we designed a **find_end_points** function to detect end points (i.e. leaf tips) using the **binary_hit_or_miss** function in the SciPy library to match the four possible 2D matrix representations:

6 $$ \begin{array}{*{20}c} 0 & 0 & 0 \\ 0 & 1 & 0 \\ \end{array}\quad          or\quad     \begin{array}{*{20}c} 0 & 1 & 0 \\ 0 & 0 & 0 \\ \end{array}\quad      or\quad     \begin{array}{*{20}c} 0 & 0 \\ 0 & 1 \\ 0 & 0 \\ \end{array}\quad      or\quad     \begin{array}{*{20}c} 0 & 0 \\ 1 & 0 \\ 0 & 0 \\ \end{array} $$

The **find_end_points** function outputs 2D coordinates of end points that correlates with leaf tips (Fig. 7b). We have employed the function to measure large or long rosette leaves, if they are over 50 or 70% of the final size (Fig. 7c and *Step_4.4.2.7* in Additional file 1). To accomplish this, we evaluated the leaf skeleton as a weighted graph and then treated: (1) the skeleton centroid and end points as *vertices* (i.e. *nodes*), (2) lines between the centre point and end points as *edges*, and (3) the leaf area and the length between vertices as *weights* assigned to each *edge*. Depending on the experiment, if the *weights* are greater than a predefined threshold (i.e. over 15 mm in length or over 100 mm^2^ in leaf size in our case), the associated leaf will be recognised as a long or large leaf. The predefined threshold is also changeable in the Notebook and HPC versions of Leaf-GP.

As the skeletonisation approach could miss very small leaves if they are close to the centroid or partially overlapping with other leaves, we therefore implemented a **leaf_outline_sweeping** module to establish another approach to detect the total leaf number based on the distance between the plant centroid and detected leaf tips. This procedure is based on a published leaf tip identification algorithm applied to three images [5]. We improved upon the algorithm for batch processing by using the leaf boundary (i.e. contour) to enhance the accuracy of the detection and reduce the computational complexity. For a given plant, the algorithm generates a distance series represents the squared Euclidean distances from the plant centroid to its contour, at angles between 0 and 359 degrees with a 1-degree interval (for presentation purposes, we used 15 degree intervals in Fig. 7d). To reduce noise, the distance series was smoothed by a Gaussian kernel (Fig. 7d). Finally, a Python-based peak detection algorithm called **PeakDetect** [58] is integrated to detect peaks on the distance series (*Step_4.4.2.8* in Additional file 1). The module implemented here supports our assumption that the number of peaks can largely represent the number of leaf tips during the batch processing (Fig. 7e, f). When quantifying the total number of leaves, results from both skeleton and outline sweeping approaches are combined to produce the number measurement. Notably, although we have generated highly correlated leaf number reading against human scoring (R^2^ = 0.924 on three image series) and between the two approaches (R^2^ = 0.903 on three series), we want to point out that the leaf number detection method is still at an early stage, requiring a joint community effort to improve its soundness and accuracy.

## Results

Leaf-GP can facilitate plant growth studies through automating trait analysis and cross-referencing results between experiments. Instead of only using machine learning algorithms to build neural network architecture for pixel clustering or trait estimates [59], we chose an approach that combines simple unsupervised machine learning, computer vision and image analysis algorithms to establish an efficient analysis framework. This approach has enabled us to generate biologically relevant outputs at both image and pot levels. Here, we exhibit three use cases where Leaf-GP were employed to study key growth phenotypes for *Arabidopsis* rosettes and *Paragon* wheat.

### Use case 1—Tracking three genotypes in a single tray

**Fig. 8:**
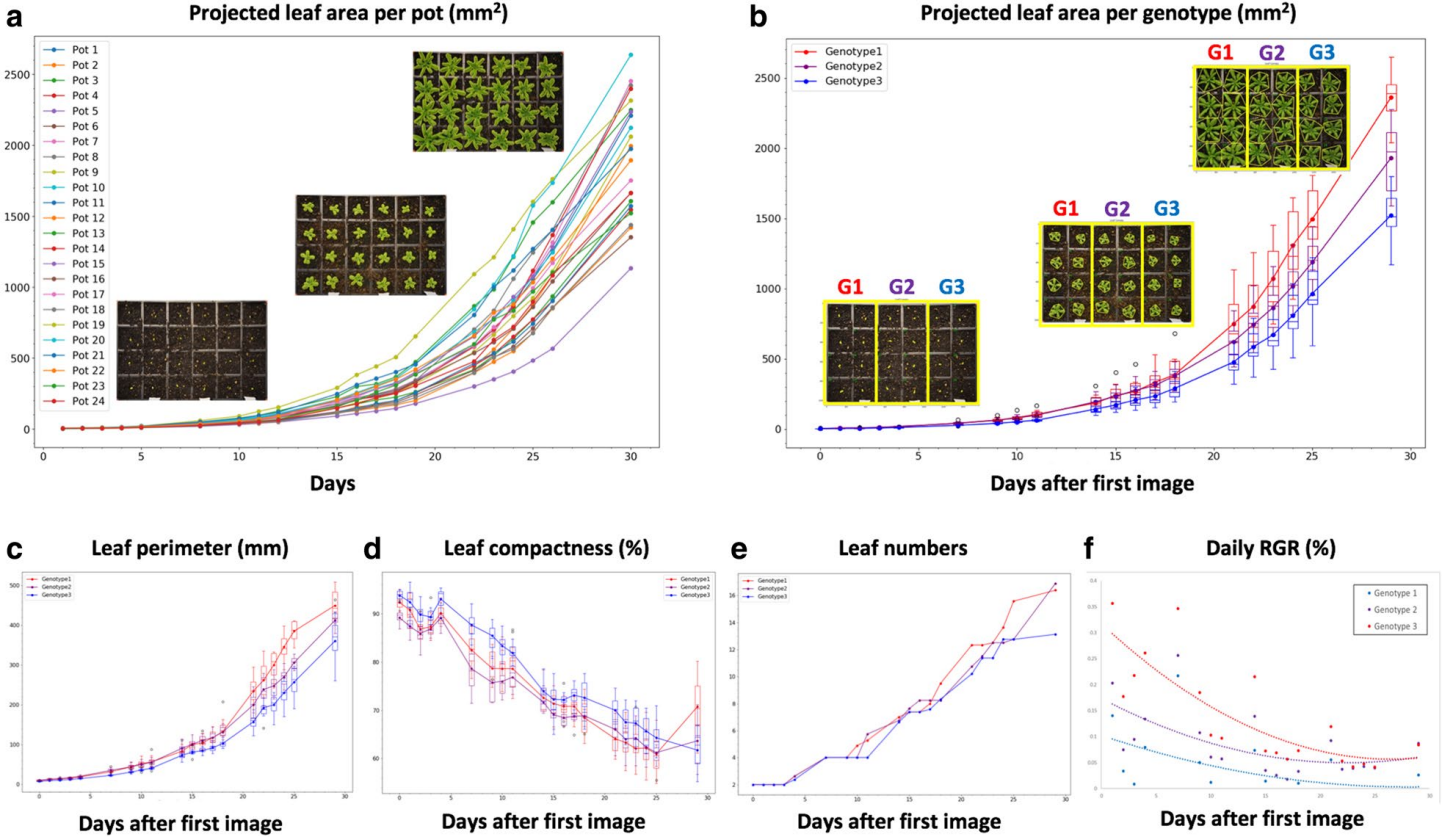
Case study 1: Analysis results of a tray with three genotypes. **a** The projected leaf area trait in 24 pots was quantified by Leaf-GP. **b** The projected leaf area trait divided into three genotype groups (i.e. G1, G2, G3). **c**–**f** A number of growth-related traits such as leaf perimeter, compactness, leaf number, and daily RGR of the three genotypes are quantified

We applied Leaf-GP to measure growth phenotypes in a tray containing three genotypes L*er* (wildtype), *spt*-*2*, and *gai*-*t6 rga*-*t2 rgl1*-*1 rgl2*-*1* (*della4)* at 17 °C. Each pot in the tray was monitored and cross-referenced during the experiment. The projected leaf area trait in 24 pots was quantified by Leaf-GP (Fig. 8a) and rosette leaves were measured from stage 1.02 (2 rosette leaves, around 5 mm^2^) to stage 5 or 6 (flower production, over 2400 mm^2^), a duration of 29 days after the first image was captured.

After dividing the quantification into three genotype groups, we used the projected leaf area readings (Fig. 8b) to verify the previously manually observed growth differences between L*er*, *spt*-*2*, and *della4* [2, 3]. Furthermore, the differences in phenotypic analyses such as leaf perimeter, compactness, leaf number, and daily RGR of all three genotypes can be differentiated (Fig. 8c–f). Particularly for Daily RGR (Fig. 8f), the three genotypes exhibit a wide variety of growth rates that verify the known genetic factors published previously [60]. Based on image series, Leaf-GP can integrate time and treatments (e.g. temperature signalling or chemicals) with dynamic growth phenotypes for cross referencing. We provided the CSV file for *Use Case 1* in Additional file 4, containing plot-level trait measurements over time. The Python script we used to plot and cross-reference pot- or genotype-based growth phenotypes is provided in Additional file 5, which is also integrated in the GUI version.

### Use case 2—Two genotypes under different temperatures

**Fig. 9:**
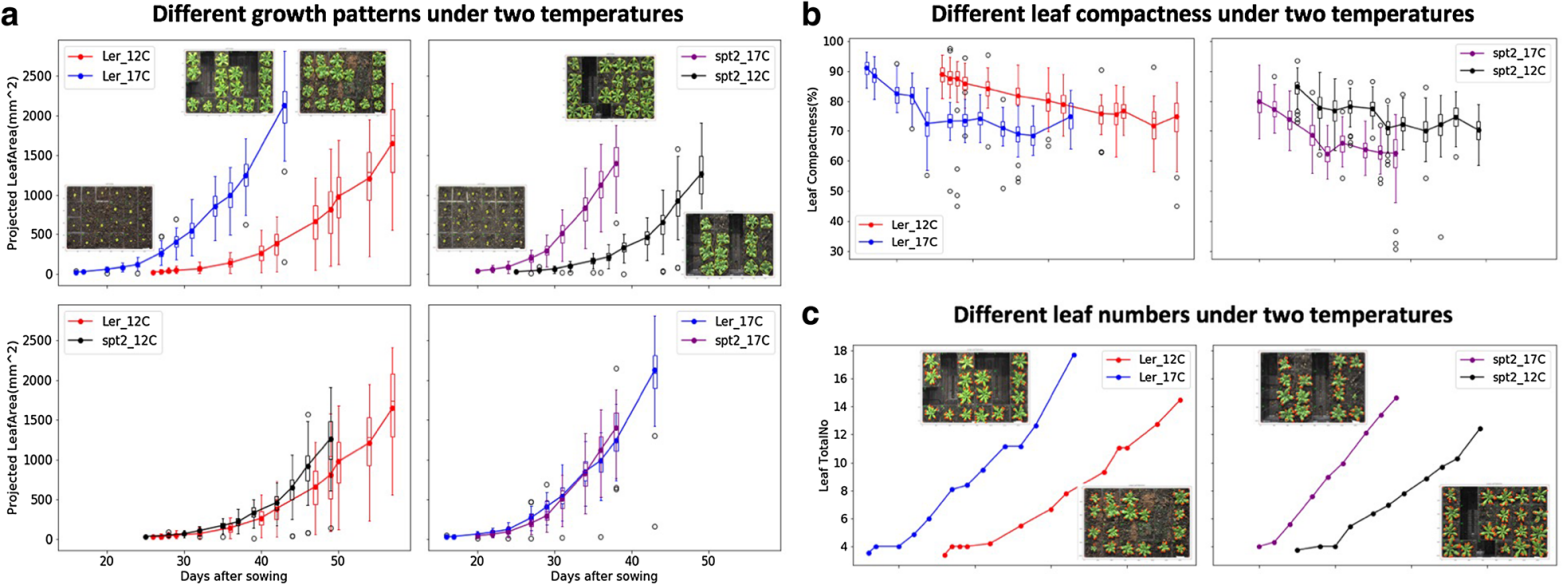
Case Study 2: Analysis results of multiple experiments. **a** The projected leaf area measurements used to observe how temperatures affect vegetative growth on both L*er* and *spt*-*2*. **b** Plants of both genotypes growing at 12 °C had more compact rosettes than those growing at 17 °C. *spt*-*2* was less compact than L*er* in general. **c** The number of leaves produced was greater at the warmer temperature

We also used the software to detect different rosette growth patterns between L*er* (wildtype) and *spt*-*2* grown at different temperatures, i.e. 12 and 17 °C. Utilising the projected leaf area measurements, we observed that temperatures affect vegetative growth greatly on both genotypes (Fig. 9a). Similar to previously studied [2, 3], lower temperatures can have a greater effect on the growth of *spt*-*2* than L*er.* Around seven weeks after sowing, the projected leaf area of *spt*-*2* was around 50% greater on average (1270 mm^2^) compared to L*er* (820 mm^2^), when grown at 12 °C (Fig. 9c). However, when grown in 17 °C, at 36 days-after-sowing *spt*-*2* had a similar area at around 1200 mm^2^, but L*er* had an area of 1000 mm^2^, a much smaller difference.

As our software can export multiple growth phenotypes, we therefore investigated both linked and independent effects of temperature on wildtype and *spt*-*2*. For instance, the larger rosette in *spt*-*2* causes a similar increase in rosette perimeter, canopy length and width, and canopy size. At similar days after sowing, plants of both genotypes grown at 12 °C had more compact rosettes that those growing at 17 °C (Fig. 9b), whereas *spt*-*2* was less compact than L*er* in general. The number of leaves produced was greater at the warmer temperature (Fig. 9c). This ability to easily export a number of key growth traits of interest is useful and relevant to broader plant growth research. We provided detailed phenotypic data (csv files) for the L*er* (12 and 17 °C, Additional file 8) and *spt*-*2* (12 and 17 °C, Additional file 9) experiments with processed images, which can be downloaded freely at https://github.com/Crop-Phenomics-Group/Leaf-GP/releases.

### Use case 3—Monitoring wheat growth

**Fig. 10:**
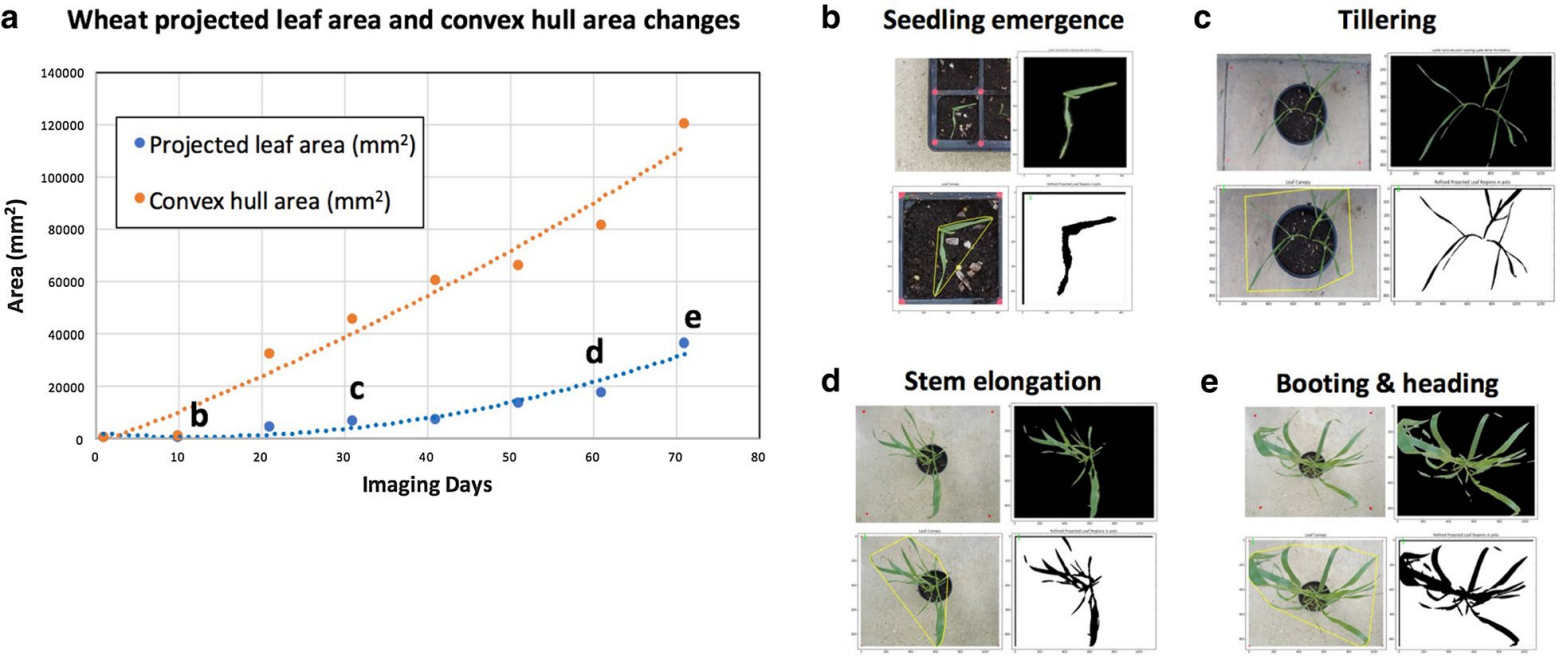
Case Study 3: Applying Leaf-GP on wheat growth studies. **a** A proof-of-concept study of how to measure the projected leaf area and the convex hull size based on *Paragon* wheat images captured over a 70-day period in greenhouse. **b**–**e** Analysis results generated from the sprouting stage to the heading stage

Another application for which Leaf-GP has been designed is to analyse wheat growth images taken in glasshouses or growth chambers using smartphones. In this case, every image only contains one wheat pot. Similarly, red circular stickers (5 mm in radius) are required to attach to the corners of the pot region so that Leaf-GP can extract ROI and transfer traits in mm units. Figure 10 demonstrates a proof-of-concept study demonstrating how Leaf-GP could be used to measure projected leaf area and leaf convex hull based on *Paragon* (a UK spring wheat) image series taken over a 70-day period in greenhouse (Fig. 10a), from sprouting (Fig. 10b) to tillering (Fig. 10c), and then from booting (Fig. 10d) to heading (Fig. 10e). With a simple and low-cost imaging setting, Leaf-GP can quantify growth phenotypes for wheat under different experimental conditions. Please note that the leaf counting function in Leaf-GP cannot be reliably applied to quantify wheat leaves due to the complicated plant architecture of wheat plants (the Notebook version for wheat can also be seen on Github, at https://github.com/Crop-Phenomics-Group/Leaf-GP/releases).

## Discussion

Different environmental conditions and genetic mutations can impact a plant’s growth and development, making the quantification of growth phenotypes a useful tool to study how plants respond to different biotic and abiotic treatments. Amongst many popularly used growth phenotypes, imaging leaf-related traits is a non-destructive and reproducible approach for plant scientists to record plant growth over time. In comparison with many published image analysis software tools for leaf phenotyping, our software provides an open and automated software framework that is capable of extracting multiple traits from large image datasets; and moreover, it can provide traits analysis that can be used to cross reference different experiments. In order to serve a broader plant research community, we designed three versions of Leaf-GP, including a GUI version for PC users, a command-line interface for HPC users, and a Notebook version for computational users. We provide all steps of the algorithm design and commented software implementation openly, together with raw and processed datasets for others to reproduce our *Arabidopsis* and *Paragon* wheat studies at NRP.

### Open software design

When developing the software, we particularly considered how to enable different plant research laboratories to utilise our work for screening large populations of *Arabidopsis* and wheat in response to varied treatments using low-cost imaging devices. Hence, we focused on software usability (e.g. simple command-line interface or GUI), capability (automatic multiple traits analyses running on different platforms), expandability (open software architecture, new functions and modules can be easily added, see the **PeakDetect** procedure in Additional file 1), and biological relevance (i.e. the extracted features are biological relevant). We trust that Leaf-GP is suitable for studying the growth performance with limited imaging hardware investment and software requirements.

The software has been used to evaluate noisy images caused by algae and different soil surfaces. Still, it can reliably execute the analysis tasks without users’ intervention. To verify Leaf-GP’s measurements, we have scored manually the key growth phenotypes on the same pots and obtained an average correlation coefficient of 0.958 on three traits (i.e. projected leaf area, leaf compactness, and leaf numbers). As the software is implemented based on open scientific libraries, it can be easily adopted or redeveloped for other experiments. We have also tested the performance of the software when handling large image datasets. Using the profile function in PyCharm [61], we recorded that the software could finish processing 2.6 GB (a series of 437 images with an average size of 6 MB) on an ordinary PC (Intel Core i5 5th generation, quad core 3 GHz, 8 GB memory) within 3 h, averagely 25 s per image.

From a software engineering perspective, we followed best practices in phenotypic analysis [62], i.e. choosing traits based on the statistical variation or dispersion of a set of phenotypic data values. Whilst implementing the software, we built on our previous work in batch processing and high-throughput trait analysis [56, 63, 64] and improved software implementation in areas such as reducing computational complexity (e.g. the usage of CPU cores and memory in parallel computing), optimising data annotation and data exchange between application programming interfaces (APIs), i.e. the objects passing between internal and external functions or methods, promoting mutual global and local feature verification (e.g. cross validating positional information at the image and the pot levels), and implementing software modularity and reusability when packaging the software. Furthermore, we verify that, instead of fully relying on a black-box machine learning approach without an in-depth understanding of why clustering or estimation is accomplished, it is more efficient to establish an analysis pipeline based on a sound knowledge of the biological challenges that we need to address. If the features we are interesting is countable and can be logically described, computer vision methods could be efficient for our phenotypic analysis missions. To support computational users to exploit our work, we have provided very detailed comments in the source code.

### The potential use of the software

From a biological perspective, the use of key growth phenotypes generated by the software can be an excellent toolkit for screening leaf growth, leaf symmetry, leaf morphogenesis and movement, e.g. phototropism. For example, the leaf skeleton is a useful tool to estimate hyponasty (curvature of the leaf). Colour features in combination with leaf convex hull could be used as a marker to quantify plant maturation, e.g. *Arabidopsis* plants transits to the reproductive stage (i.e. flowering), a change from vegetative to flowering meristem when cauline leaves are produced. Some phenotypes are also useful in studies other than plant development biology, for instance, vegetative greenness can be used in plant pathogen interaction to analyse the activity of pathogens on the leaf surface, as most of the times broad yellowish symptoms can be observed from susceptible plants (e.g. rust in wheat).

## Conclusions

In this paper, we presented Leaf-GP, a sophisticated software application for analysing large growth image series to measure multiple growth phenotypes in response to different treatments over time. We demonstrated that treatment effects between genotypes could be detected reliably by the software. We also showed the usefulness and the accuracy of the analysis based on quantifying growth traits for *Arabidopsis* genotypes under varied temperature conditions and wheat growth in the glasshouse. To serve a broader plant research community, we improved the usability of the software so that it can be executed on different platforms. To help users to gain an in-depth understanding of the algorithms and the software, we have provided our source code, detailed comments, software modulation strategy, and executables (.exe and .app), together with raw image data and processing results in this paper as well as at https://github.com/Crop-Phenomics-Group/Leaf-GP/releases.

Leaf-GP software can be used without programming skills and limited requirements on imaging equipment. Our software has confirmed previously reported results in the literature, which can be reproduced in other plant growth studies. Our case studies of temperature effects and different genotypes or plant species are not limited. Many plant growth and development experiments can be analysed by Leaf-GP, for example, natural variation in plant growth, or plants experiencing mineral or nutrient stress.

## Additional files

**Additional file 1.** The interactive *Jupyter Notebook version* for Leaf-GP (version 1.18).

**Additional file 2.** Install manual for Python environment, Anaconda Python distribution and OpenCV-Python binding.

**Additional file 3.** Processed images of *Arabidopsis* rosettes at different growth stages.

**Additional file 4.** Multiple trait measurements results based on a testing series.

**Additional file 5.** The *Jupyter Notebook version* for plotting and cross-referencing growth traits between experiments.

**Additional file 6.** The analysis workflow and a detailed activity diagram of Leaf-GP.

**Additional file 7** The manual for importing image datasets via the GUI version of Leaf-GP.

**Additional file 8.** Multiple trait measurements results based on L*er* 12, 17, 22 °C.

**Additional file 9.** Multiple trait measurements results based on *spt*-*2* 12, 17, 22 °C

## Abbreviations

RGB: A red, green and blue colour model
NoIR: No infrared filter
ROI: Regions of interest
GUI: Graphic user interface
HPC: High-performance computer
CSV: Comma-separated values
OS: Operating systems
CPU: Central processing unit
Lab: Lightness, a for the colour opponents green–red, and b for the colour opponents blue–yellow
RGR: Relative growth rate
L*er*: Landsberg *erecta* (wildtype)
*spt*-*2*: Spatula-2
API: Application programming interfaces
