# Supplementary material for: Leaf-GP: an open and automated software application for measuring growth phenotypes for arabidopsis and wheat
Source: Plant Methods. 2017 Dec 22;13:117. doi: 10.1186/s13007-017-0266-3 (PMC5740932; doi:10.1186/s13007-017-0266-3)
Supplement: Supplementary file 1 — Additional file 1. The interactive Jupyter Notebook version for Leaf-GP (version 1.18). [file 13007_2017_266_MOESM1_ESM.html]

LeafGP-V1.18-All\_Platforms-Final


In [1]:

```
###################################################################
#                      Document History                           #
#                                                                 #
#    Author: Dr Ji Zhou (EI/JIC)                                  #         
#               <ji.zhou@tgac.ac.uk>                              # 
#            Dr Christopher Applegate (The Zhou lab, EI)          #
#               <Christopher.Applegate@earlham.ac.uk>             #
#            Dr Nick Pullen (JIC)                                 #
#               <Nick.Pullen@jic.ac.uk>                           #
#                                                                 #
#    Date: 7th July 2015, V0.1 24th June 2015                     #
#    Version: 0.75 on TGAC internal Github                        #
#    SciPy version: 0.15.1, Scikit-image: 0.11.2                  #
#    Changes: 1) Modulate the code                                #
#             2) Changed the setting on ruler, LAB and            #
#                pot segmentation                                 #
#                                                                 #
#    Date: 7th October 2015                                       #
#    Version: 0.95 on TGAC internal Github                        #
#             Version 0.1-0.95 are based in testing box HPC       #
#    New libraries:                                               #
#             OpenCV python binding                               #
#    Changes: 1) Add red reference points detection               #
#             2) Redesign the pot segmentation                    # 
#             3) Optimised for batch processing                   # 
#             4) fix detection and quantification bugs            # 
#                                                                 #
#    Date: 25th October 2015                                      #
#    Version: 0.975 on TGAC internal Github                       #
#             Version 0.1-0.975 are based in testing box HPC      #
#    Software license: BSD license, the algorithm below is        #
#    developed by Dr Zhou and his team, which shall only be       #
#    used for research purpose only.                              #
#                                                                 #
#    Changes: 1) Changed the LUV colour spacing                   #
#             2) Redesign leaf filtering at the pot level         # 
#             3) Optimised codes for memory consumption           # 
#                                                                 #
#    Date: March 2016                                             #
#    Version: 0.99 optimised for HPC clusters...                  #
#    Changes: 1) New red dot identifier as NP screwed up photos   #
#                now based on difference between R & G channel    #
#             2) Calculate area in mm^2 not just pixels           #
#                area average (but should just use 2 biggest)     #
#             3) Write all results to 1 file not separate ones    #
#             4) Put in plt.close() to save memory                #
#             5) Enable garbage collection                        #
#             6) Rescale images to 0.25 (means some size params   #
#                have needed to change e.g. region.area)          #
#             7) Main loop is in try/except for graceful failure  #
#                and moving on to next image                      #
#                                                                 #
#    Date: September 2016                                         #
#    Version: 1.0                                                 #
#    Changes: 1) Add kmeans to improve the precision of leaf      #
#                segmentation                                     #
#             2) Improve object shape detection                   #
#             3) Improve leaf measurement for various lighting    #
#             4) Rectify to remove small objects with different   #
#                LAB value - greenness                            #
#             5) Build the HPC version                            #
#                                                                 #
#    Date: January 2017                                           #
#    Version: 1.05                                                #
#    Changes: 1) Improve the precision of segmentation            #
#             2) Refine the output csv and processed images       #
#             3) Add excessive greenness for internal detection   #
#                                                                 #
#    Date: May 2017                                               #
#    Version: 1.1                                                 #
#    Changes: 1) Add a skeleton function for leaf detection       #
#             2) Use graph theory to seek start/end points        #
#                as well as skeleton joint points                 #
#             3) Add a GUI and package the software for release   #
#                                                                 #
#    Date: May 2017                                               #
#    Version: 1.15                                                #
#    Changes: 1) Refine the skeleton function for big leaf        #
#                detection                                        #
#             2) Refine a GUI to control workflow and high-       #
#                throughput image processing tasks                #
#             3) Test and configure for all OSs, including        #
#                Windows and Mac OSs                              #
#                                                                 #
#    Date: May 2017                                               #
#    Version: 1.16-1.18                                           #
#    Changes: 1) Hanlde various image file names                  #
#             2) A new leaf detection method                      #
#             3) profiled code for distribution                   #
#             4) optimsed for paralle computing                   #
#                                                                 #
###################################################################

#STEP 1: Import libraries 
% matplotlib inline

# Essential libraries 
import numpy as np
import scipy as sp

# Computer vision libraries 
from scipy import ndimage
from skimage.color import rgb2gray
from skimage import io 
# All other skimage functions will be listed with the code fragments below
import cv2
from matplotlib import pyplot as plt # Plotting and generating figures

# Other libraries
import math # Feature measures 
import os # Access the file system
import gc # Garbage collection 
import csv # For results output
import sys # for operating systems 
gc.enable()
```

In [2]:

```
#STEP 2: Enable GUI interactions for users to select an image file 
#        in iPython Notebook to locate an image series

##STEP 2.1: GUI dialog panel to locate where to import images 
import Tkinter, tkFileDialog

# Make a top-level instance, but without displaying it
# Use Tkinter for GUI interactions 
root = Tkinter.Tk() 
root.withdraw()
root.overrideredirect(True) 

# Invisible without decorations, 0 size, to the top left corner.
root.geometry('0x0+0+0')
root.deiconify() # show window and lift it to top position
root.lift() # Place the dialog panel before the terminal.
root.focus_force()

# Assign the selected filename with a full path
fullname = tkFileDialog.askopenfilename(parent=root)
root.destroy() 
# get rid of the top-level instance 
# Finish read the full path from local file system
```

In [3]:

```
import PeakDetect as pd 
# import the PeakDetect py
# Copyright (C) 2016 Sixten Bergman
# License WTFPL
#
# The PeakDetect program is free software. It comes without any warranty, to the extent
# permitted by applicable law.
# You can redistribute it and/or modify it under the terms of the Do What The
# **** You Want To Public License, Version 2, as published by Sam Hocevar. See
# http://www.wtfpl.net/ for more details.
```

In [4]:

```
# Function_0 
# Cross-platform delimiter 

def PlatformDelimiter(): 
    """Generate Delimiter based on Platforms"""
    # Current OS 
    OS_Delimiter = ''
    if sys.platform == 'win32':
        # windows codes
        OS_Delimiter = '\\'
    elif sys.platform == 'darwin':
        # mac codes
        OS_Delimiter = '/' 
    else:
        # other code here (linux)
        OS_Delimiter = '/' 
    # Return the delimiter     
    return OS_Delimiter
```

In [5]:

```
##STEP 2.2: Reassemble the directory of the selected image 
import glob # Find all the pathnames matching a specified pattern

# Get the full path of the image
fullPath = fullname.split("/", -1) 
# The following can handle different platforms
Platform_Delimiter = PlatformDelimiter()
fullPathRef = Platform_Delimiter.join(fullPath[: -1]) + Platform_Delimiter
print 'The selected image: ', fullname
print 'The full path: ', fullPathRef

##STEP 2.3 Start to prepare a folder to save processing results
CurrentDir = os.getcwd()
# print CurrentDir 
tmp_Folders = [] 
tmp_Folders_Final = []
workingRootFolder = fullPathRef
# Locate the image directoriy 
print "The working directory: ",workingRootFolder

# Loop through the working folder, testing for batch processing 
for root, dirs, files in os.walk(workingRootFolder):
    Work_Dir_Root = root
    tmp_Folders.append(dirs)
    tmp_Folders_Final = filter(None, tmp_Folders) 
    # A fast way to get rid of empty element while using os.walk 
    # when going through a file instead of a folder
    
# Directories found in the root folder 
if not len(tmp_Folders_Final) == 0:
    workDirFolders = tmp_Folders_Final[0] 
else: 
    workDirFolders = workingRootFolder
# First level of the working directory, use ONE to search the second level of the directory
# In case this folder has been processed
print "Folders found in the selected directory: ", workDirFolders
```

```
The selected image:  /Users/zhouj/Documents/My papers/LeafSeg/Publication Testing/Testing/2016-07-11_Ler_22C_Tray_4.jpg
The full path:  /Users/zhouj/Documents/My papers/LeafSeg/Publication Testing/Testing/
The working directory:  /Users/zhouj/Documents/My papers/LeafSeg/Publication Testing/Testing/
Folders found in the selected directory:  ['Processed_2017-6-12', 'Processed_2017-6-19']
```

In [6]:

```
# Function_1 
# Return the current date and time based on the OS
import time
from datetime import datetime, timedelta

def LocalTimeStamp(): 
    """Detect curret data and time"""
    # Current OS date and time 
    currentDate = time.localtime(time.time())
    return currentDate
```

In [7]:

```
##STEP 2.4: Set up a result folder to contain the processing results  
# Sort different image datasets included in the GUI system 

# Get the pre-processing date 
timeStamp = LocalTimeStamp()
curYear = timeStamp[0]
curMonth = timeStamp[1]
curDay = timeStamp[2]

# Assemble a result folder for processed results
Result_Folder =  'Processed_%d' %curYear + '-%d' %curMonth + '-%d' %curDay
Result_Directory = workingRootFolder + Result_Folder

# If the user repeats the image selection with different criteria 
if not os.path.exists(Result_Directory):
    os.makedirs(Result_Directory)
# Folder for processed results 
print 'Result folder: ', Result_Directory
```

```
Result folder:  /Users/zhouj/Documents/My papers/LeafSeg/Publication Testing/Testing/Processed_2017-6-21
```

In [8]:

```
# Function_2
# Locate the red reference points 

def RefPoints(ref_image, blank_image):
    """Locate red reference points"""
    # STEP 1: Locate red pixels
    # R >= 150, B <= 100, and G <= 100, due to colour distortion  
    Ref_Points_Red = ref_image[:, :, 0] > 125
    # Use blue channel to remove the white pixels
    Ref_Points_Blue = ref_image[:, :, 2] < 225
    Ref_Points_Color_Selection_1 = np.logical_and(Ref_Points_Red, Ref_Points_Blue)
    # Add second approach based on the difference between red and green channles 
    Ref_Points_Color_Selection_2 = np.array(ref_image[:,:,0], dtype='int')-np.array(ref_image[:,:,1], dtype="int")>50
    
    # STEP 2: Extract Red ref points from the previous mask 
    Ref_Points_Refined = np.logical_and(Ref_Points_Color_Selection_1, Ref_Points_Color_Selection_2)
    Ref_Point = remove_small_objects(Ref_Points_Refined, 125) # get rid of small pixels, as the resolution is fixed 
    Fill_Ref_Points = ndimage.binary_fill_holes(Ref_Point)

    ## Create a list for the areas of the detected red circular reference points 
    Ref_Point_Areas = []
    Ref_Point_Array = []
    # Start to Label the Red reference points
    Labelled_Ref_Point = label(Fill_Ref_Points, connectivity =1)
    processed_Ref_Img = blank_image.copy()
    Obj_counter = 0
    maxLength = Ref_Points_Refined.shape[1]/25
    minLength = Ref_Points_Refined.shape[1]/55
    # Go through every red reference point objects
    for region in regionprops(Labelled_Ref_Point): 
        Ref_Tmp_Img = blank_image.copy()
        # Purely based on morphology features
        if region.area < math.pi*(minLength/2)**2 or region.area > math.pi*(maxLength/2)**2:
            # set area lower and upper bounds
            continue                                  
        if region.solidity < 0.75: # Compare ref points with their convex areas.
            continue
        if region.eccentricity > 0.75: # These ref points are not totally round.
            continue
        # get 2D coordinates of the ref points
        Obj_counter = Obj_counter + 1 
        # print "ID: ", str(Obj_counter), 'Centroid: ', int(region.centroid[0]), int(region.centroid[1])
        pot_row_max = [region.bbox[2], ref_image.shape[0]]
        # column first (y) and then row (x)
        Ref_Point_Array.append([int(region.centroid[1]), int(region.centroid[0])])
        Ref_Point_Areas.append(region.area)
        Ref_coord = region.coords.astype(int)
        Ref_Tmp_Img[Ref_coord[:,0], Ref_coord[:,1]] = 1
        # Generate the final mask
        processed_Ref_Img = np.logical_or(processed_Ref_Img, Ref_Tmp_Img)
        
    # Set up empty arraies for boundary
    Upper_Left_Point = []
    Lower_Left_Point = []
    Upper_Right_Point = []
    Lower_Right_Point = []

    # Sort the position of red reference points on the image 
    for x_coor, y_coor in Ref_Point_Array:
        if x_coor < ref_image.shape[1] * 0.75 and y_coor < ref_image.shape[0] * 0.75:
            Upper_Left_Point.append([x_coor, y_coor])
        if x_coor < ref_image.shape[1] * 0.75 and y_coor > ref_image.shape[0] * 0.75:
            Lower_Left_Point.append([x_coor, y_coor])
        if x_coor > ref_image.shape[1] * 0.75 and y_coor < ref_image.shape[0] * 0.75:
            Upper_Right_Point.append([x_coor, y_coor])
        if x_coor > ref_image.shape[1] * 0.75 and y_coor > ref_image.shape[0] * 0.75:
            Lower_Right_Point.append([x_coor, y_coor])

    Ref_Point_Array_Ref = []        
    Ref_Point_Array_Ref.append(Upper_Left_Point[0])
    Ref_Point_Array_Ref.append(Upper_Right_Point[0])
    Ref_Point_Array_Ref.append(Lower_Left_Point[0])
    Ref_Point_Array_Ref.append(Lower_Right_Point[0])

    # Return the Red reference points' coordindates
    return Ref_Point_Array_Ref, Ref_Point_Areas, processed_Ref_Img
```

In [9]:

```
# Function_3
# Calcuale the pixel to mm conversion 

def convert_pixels_to_mm(pixels_in_circ, circle_width):
    """pixels_in_circ is the array of `region.area`s"""
    averagePixelArea = np.mean(pixels_in_circ)
    # Calculate the pixel to mm conversion rate 
    pixels_per_mm = round(math.sqrt(averagePixelArea/math.pi)/circle_width, 2)
    return(pixels_per_mm)
```

In [10]:

```
# Function_4
# Flatten an image for pixel rescaling  

def flatten_img(img):
    """Convert an image with size (M, N, 3) to (M * N, 3). 
    Flatten pixels into a 1D array where each row is a pixel and the columns are RGB values.
    """
    # The image needs to contain 3 channels...
    result_image = img.reshape((np.multiply(*img.shape[:2]), 3))
    return result_image
```

In [11]:

```
# Function_5
# Perform perspective transformation, OpenCV is used to improve the performance 

def PerspectiveTrans_2D(ref_image, Ref_Point_Array):
    """Perform perspective transformation in 2D"""
    # Read the original image for 2D resolution 
    img = ref_image.copy()
    rows,cols,ch = img.shape
    # rows - y axis; cols - x axis 
    Reference_Distance = math.sqrt((Ref_Point_Array[0][0]-Ref_Point_Array[1][0])**2 + 
                                 (Ref_Point_Array[0][1]-Ref_Point_Array[1][1])**2)
    Tray_Cols = 0
    if Reference_Distance > cols * 0.75:
        # This is a full tray 5*8 or 4*6
        columns = cols
    else: 
        # This is a half tray 5x4 or 4*4
        columns = cols/2

    # Red points positions on the original image 
    pts1 = np.float32([Ref_Point_Array[0], Ref_Point_Array[1], Ref_Point_Array[2], Ref_Point_Array[3]])
    # Positions on the transformed image
    pts2 = np.float32([[0,0], [columns,0], [0,rows], [columns, rows]])
    # Using 2D list, not an array
    # This will be returned for following process
    New_Ref_Points_Array = [[0,0], [columns,0], [0,rows], [columns, rows]]
    # Use OpenCV to perform the perspective transformation
    Transform_Array = cv2.getPerspectiveTransform(pts1, pts2)
    Transformed_img = cv2.warpPerspective(img, Transform_Array,(columns, rows)) # x, y

    # Return the transformed image and reference points array
    return Transformed_img, New_Ref_Points_Array
```

In [12]:

```
# Function_6
# Generate segmented pots, 1x1 is accepted 

from skimage.draw import line_aa 
# anti-aliased, to segment the pots

def PotSegmentation(img, row_no, column_no, Ref_Points_Array):
    """Generate an image to contain segmented pots"""
    # Generate a blank image to contain segmented pots  
    Blank_Img = np.zeros((img.shape[0], img.shape[1]), dtype=np.uint8)
    Pot_Image = Blank_Img.copy()
    Seg_Img_tmp = Blank_Img.copy()
    tmp_line_img = Blank_Img.copy()

    # Set up empty arrays for boundary
    Upper_Left_Point = []
    Lower_Left_Point = []
    Upper_Right_Point = []
    Lower_Right_Point = []

    # Read x,y coordinates from the reference points array
    for x_coor, y_coor in Ref_Points_Array:
        if x_coor < img.shape[0] * 0.5 and y_coor < img.shape[1] * 0.5:
            Upper_Left_Point.append([x_coor, y_coor])
        if x_coor < img.shape[0] * 0.5 and y_coor > img.shape[1] * 0.5:
            Lower_Left_Point.append([x_coor, y_coor])
        if x_coor > img.shape[0] * 0.5 and y_coor < img.shape[1] * 0.5:
            Upper_Right_Point.append([x_coor, y_coor])
        if x_coor > img.shape[0] * 0.5 and y_coor > img.shape[1] * 0.5:
            Lower_Right_Point.append([x_coor, y_coor])
    # print Upper_Left_Point, Lower_Left_Point, Upper_Right_Point, Lower_Right_Point  

    # Start to segment pots - rows
    # Add the bottom line!!!
    for i in range(row_no): # Draw the top/bottom lines
        # row_no + 1 lines will be produced
        range_row = int((i * (Upper_Left_Point[0][1] + Lower_Left_Point[0][1])/row_no))  
        # In case the calculation is within the image boundary 
        if (Upper_Left_Point[0][1] + range_row) < (img.shape[0] - 5): 
            # Draw lines in rows, make it a little bit skew to correct labelling issue
            rr, cc, val = line_aa(Upper_Left_Point[0][1] + range_row, Upper_Left_Point[0][0], 
                                  Upper_Right_Point[0][1] + range_row + 1, Upper_Right_Point[0][0]-1)
        else: # out of the image boundary 
            rr, cc, val = line_aa(img.shape[0]-5, Upper_Left_Point[0][0], 
                                  img.shape[0]-5, Upper_Right_Point[0][0]-1)
        # At y-axis will be 0..shape[1]-1
        tmp_line_img[rr, cc] = val * 255
        Pot_Image = np.logical_or(Pot_Image, tmp_line_img)   

    # Start to segment pots - columns
    tmp_line_img = Blank_Img.copy() # reset the temp line image
    for j in range(column_no):
        # column_no + 1 lines will be produced 
        range_col = int((j * (Upper_Left_Point[0][0] + Upper_Right_Point[0][0])/column_no)) 
        # In case the calculation is within the image boundary 
        if (Upper_Left_Point[0][0] + range_row) < (img.shape[1]-5): 
            # Draw lines in columns  
            rr, cc, val = line_aa(Upper_Left_Point[0][1], Upper_Left_Point[0][0] + range_col, 
                                  Lower_Left_Point[0][1]-1, Lower_Left_Point[0][0] + range_col)   
        else: # out of the image boundary
             rr, cc, val = line_aa(Upper_Left_Point[0][1], img.shape[1]-5, 
                                   Lower_Left_Point[0][1]-1, img.shape[1]-5)
        # At x-axis will be 0..shape[0]-1
        tmp_line_img[rr, cc] = val * 255
        Pot_Image = np.logical_or(Pot_Image, tmp_line_img)    

    # Dilate the pot image    
    selem = disk(3)
    Pot_Image = dilation(Pot_Image, selem)
    # Find the segment region
    Pot_Segment = np.logical_not(Pot_Image) 
    Pot_Segment_Refine = erosion(Pot_Segment, disk(4))

    # Return pot and segment border images  
    return Pot_Image, Pot_Segment_Refine
```

In [13]:

```
# Function_7
# Non-linear LAB based green region extraction 

def LAB_Img_Segmentation(img, pix2mm):
    """Extract green regions from the image"""
    # Colour space with dimension L for lightness 
    # a and b for the colour-opponent dimensions, based on nonlinearly compressed 
    
    # Colour space with dimension L for lightness 
    # a and b for the colour-opponent dimensions, nonlinearly compressed CIELAB
    leaf_img_LAB = color.rgb2lab(img)
    # color-opponent - negative values indicate green and hence are retained
    a_2D_Color_ND = leaf_img_LAB[:, :, 1] 
    # color-opponent - positive values indicate yellow, which means bright objects will be retained
    b_2D_Color_ND = leaf_img_LAB[:, :, 2] 
    LAB_Image_Ref = (b_2D_Color_ND - a_2D_Color_ND)
    # Use global adaptive thresholding to seek the threshold, as the image has been denoised
    global_thresh_Ref_Points_Value = filters.threshold_otsu(LAB_Image_Ref) 
    # Use one standard deviation to segment intensity distribution, if the otus value is too low  
    if global_thresh_Ref_Points_Value > 0 and global_thresh_Ref_Points_Value >= LAB_Image_Ref.max() * 0.425:
        binary_global_LAB = LAB_Image_Ref > LAB_Image_Ref.max() * 0.75 # close to 32% - tails of 1SD
    elif global_thresh_Ref_Points_Value > 0 and global_thresh_Ref_Points_Value < LAB_Image_Ref.max() * 0.425:  
        binary_global_LAB = LAB_Image_Ref > LAB_Image_Ref.max() * 0.35 # close to 68% - 1SD 
        # Close to 2 standard deviations, Was using 0.25 for 1SD
    else: # global_thresh_Ref_Points_Value < 0, in very rare cases 
        binary_global_LAB = LAB_Image_Ref > 0

    # 1-pixel regions, expanded 1 pixel, which needs to be rescaled during quantification  
    Img_cleaned = remove_small_objects(binary_global_LAB, pix2mm * 2.5) 
    # use the pixel and metrics ratio, as small leaves are around 10 mm^2
    # Do not fill images, as the first two true leaves are not touched
    selem = disk(3) 
    Img_cleaned_Dilated = dilation(Img_cleaned, selem)
    Img_cleaned_Ref = remove_small_holes(Img_cleaned_Dilated, min_size=int(pix2mm * 3.25)**3)
    # In total 1 pixels have been expanded around the outline of every object
    erode_binary_Img = erosion(Img_cleaned_Ref, disk(2))
    erode_binary_Img = remove_small_objects(erode_binary_Img, pix2mm * 5.25) 
    # use the pixel and metrics ratio, as dilated small leaves are around 25 mm^2

    # Leaf segmented image based on LAB colour spacing
    return erode_binary_Img, leaf_img_LAB
```

In [14]:

```
# Function_8 
# Generate an image that is represented by excessive greenness and excessive red 

# normalise the specified numpy matrix so that it is in range [0,1]
# @param mat: the numpy matrix to normalise
def norm_range(mat, min_val, max_val):
    """Normalise a specified numpy matrix"""
    # get the range
    range = max_val - min_val
    # as long as the range is not 0 then scale so that range is 1
    if range > 0:
        # subtract offset so min value is 0
        mat -= min_val
        # normalise so values are in range 0
        mat /= float(range)

# return excessive green representation of a provided image
# @param img: RGB image needs to be converted (np.uint8)
def compute_greenness_img(img):
    """Transfer a given image to excessive greenness and excessive red"""
    # convert to floating point representation [0, 1]
    img = img.astype(np.float64) / 255.0
    # split image into its r, g, and b channels
    r, g, b = cv2.split(img)
    # create 2D sum matrix (element-wise addition of r, g, and b values
    sum = r + g + b
    # divide each colour channel by the sum (element-wise)
    r = np.divide(r, sum)
    g = np.divide(g, sum)
    b = np.divide(b, sum)
    # compute excessive green image
    ex_g = 2.0 * g - r - b
    # compute excessive red image
    ex_r = 1.4 * r - b
    # compute vegetative image (excessive green - excessive red)
    veg = ex_g - ex_r
    # noramlsie the image
    norm_range(veg, -2.4, 2.0) # -2.4 is the minimum veg value (1, 0, 0) and 2.0 is maximum veg value (0, 1, 0)
    # convert back to 8-bit unsigned int representation [0, 255]
    veg = veg * 255
    veg = veg.astype(np.uint8)
    # return the vegetative image
    return veg
```

In [15]:

```
# Function_9
# Use kmeans to precisely segment pixel groups 

def kmeans_cluster(img, k_value):
    """Cluster pixels"""
    # As requested by OpenCV, transfer to float32
    kmeans_img = np.float32(img.reshape((-1,1)))
    # Define criteria, number of clusters(K) and apply kmeans()
    criteria = (cv2.TERM_CRITERIA_EPS + cv2.TERM_CRITERIA_MAX_ITER, 10, 1.0)
    # cv2.TERM_CRITERIA_EPS stops the algorithm iteration if specified accuracy is reached. 
    # cv2.TERM_CRITERIA_MAX_ITER stops the algorithm after the specified number of iterations is reached 
    if k_value > 10:
        k_value = 10 # k_value should be less than 10, otherwise the classificaiton will be too slow
    # Four groups were determined based on distances NearestNeighbors 
    # 1) leaf, 2) soil/compost, 3) reference points, 4) others such as reflection
    #..! OpenCV 2.4.11 version below 
    ret,label,center=cv2.kmeans(kmeans_img, k_value, criteria, 10, cv2.KMEANS_RANDOM_CENTERS)
    # cv2.KMEANS_RANDOM_CENTERS - for cluster centers, not required in the analysis
    # Convert the image back to uint8
    center = np.uint8(center)
    res = center[label.flatten()]
    res2 = res.reshape((img.shape))
    # return the kmeans image
    return res2
```

In [16]:

```
# Function_10
# Use graph theory to locate end or branching points of a skeleton 

from scipy.ndimage import binary_hit_or_miss

def find_end_points(skel):
    """Detect end points of a skeleton"""
    # Four possible matrix representation
    struct1, origin1 = np.array([
            [0, 0, 0],
            [0, 1, 0],
        ]), (0, 0)
    struct2, origin2 = np.array([
            [0, 0],
            [0, 1],
            [0, 0],
        ]), (0, 0)
    struct3, origin3 = np.array([
            [0, 1, 0],
            [0, 0, 0],
        ]), (-1, 0)
    struct4, origin4 = np.array([
            [0, 0],
            [1, 0],
            [0, 0],
        ]), (0, -1)
    
    # Match end point structures with the skeleton
    ret = None
    for i in range(1, 5):
        struct, origin = locals()['struct%d' % (i)], locals()['origin%d' % (i)]
        if ret is None:
            ret = binary_hit_or_miss(skel, structure1=struct, origin1=origin)
        else:
            ret = np.logical_or(ret, binary_hit_or_miss(skel, structure1=struct, origin1=origin))
    return np.transpose(np.nonzero(ret)[::-1])

def find_branches(skel):
    """Detect branching points of a skeleton"""
    # Five possible matrix representation
    struct1 = np.array([
        [0, 1, 0],
        [0, 1, 0],
        [1, 0, 1]
    ])   
    struct2 = np.array([
        [1, 0, 0],
        [0, 1, 1],
        [0, 1, 0]
    ])
    struct3 = np.array([
        [1, 0, 0],
        [0, 1, 0],
        [1, 0, 1]
    ])    
    struct4 = np.array([
        [0, 0, 0],
        [1, 1, 1],
        [0, 1, 0]
    ])
    struct5 = np.array([
        [0, 1, 0],
        [1, 1, 1],
        [0, 1, 0]
    ])
    
    # Match branching point structures with the skeleton
    structs = [np.rot90(struct1, i)   for i in range(4)]
    structs += [np.rot90(struct2, i)   for i in range(4)]
    structs += [np.rot90(struct3, i)   for i in range(4)]
    structs += [np.rot90(struct4, i)   for i in range(4)]
    structs += [np.rot90(struct5, i)   for i in range(4)]
    
    ret = None
    for i in range(len(structs)):
        if ret is None:
            ret = binary_hit_or_miss(skel, structure1=structs[i])
        else:
            ret = np.logical_xor(ret, binary_hit_or_miss(skel, structure1=structs[i]))
    return np.transpose(np.nonzero(ret)[::-1])
```

In [17]:

```
# Function_11
'''Leaf number detection based on a sweeping method to go through the leaf outline waveform'''  

def draw_line(img, cx, cy, rad, colour):
    h, w = img.shape[:2]
    diagonal = math.sqrt(h * h + w * w)
    x = diagonal * math.cos(rad)
    y = diagonal * math.sin(rad)
    x0 = int(round(cx))
    y0 = int(round(cy))
    x1 = int(round(x0 + x))
    y1 = int(round(y0 + y))
    cv2.line(img, (x0, y0), (x1, y1), colour, 3)
    return img

def sweeping(img, cx, cy):
    h, w = img.shape[:2]
    diagonal = math.sqrt(h * h + w * w)
    distances2 = []
    points = []
    # Generate a mask to contain radians, 0-360
    mask = np.zeros((h, w), np.uint8)

    for i in range(0, 360):
        mask[:, :] = 0
        rad = math.radians(i)
        x = diagonal * math.cos(rad)
        y = diagonal * math.sin(rad)
        x0 = int(round(cx))
        y0 = int(round(cy))
        x1 = int(round(x0 + x))
        y1 = int(round(y0 + y))
        cv2.line(mask, (x0, y0), (x1, y1), (255), 3)
        indices = np.nonzero(np.bitwise_and(img, mask))
        max_dist2 = 0
        max_dist2_point = (cx, cy)
        for j in range(len(indices[0])):
            iy = indices[0][j]
            ix = indices[1][j]
            dx = ix - cx
            dy = iy - cy
            dist2 = dx * dx + dy * dy
            if dist2 > max_dist2:
                max_dist2 = dist2
                max_dist2_point = (ix, iy)
        distances2.append(max_dist2)
        points.append(max_dist2_point)

    return distances2, points

def find_peaks(data):
    [peaks, _] = pd.peakdetect(np.array(data), lookahead=5)
    indices = []
    values = []
    for peak in peaks:
        indices.append(peak[0])
        values.append(peak[1])
    return np.array(indices), np.array(values)

def smooth_series(data, half_kernel_size, sigma=-1):
    kernel_size = half_kernel_size * 2 + 1
    kernel = list(cv2.getGaussianKernel(kernel_size, sigma)[:, 0])
    new_data = [0] * len(data)
    for i in range(len(data)):
        values = []
        for j in np.arange(-half_kernel_size, half_kernel_size + 1):
            weighted_value = data[(i + j) % len(data)] * kernel[j + half_kernel_size]
            values.append(weighted_value)
        new_data[i] = np.mean(values)
    return new_data

def find_leaves(img, out_img, cx, cy, pot_region, font=cv2.FONT_HERSHEY_SIMPLEX, font_size=1, font_thickness=2):

    img = img.astype(np.uint8) * 255
    bb_y0, bb_x0, bb_y1, bb_x1 = pot_region.bbox

    try:
        out_img[bb_y0:bb_y1 + 1, bb_x0:bb_x1 + 1, 0] = img[bb_y0:bb_y1 + 1, bb_x0:bb_x1 + 1]
        out_img[bb_y0:bb_y1 + 1, bb_x0:bb_x1 + 1, 1] = img[bb_y0:bb_y1 + 1, bb_x0:bb_x1 + 1]
        out_img[bb_y0:bb_y1 + 1, bb_x0:bb_x1 + 1, 2] = img[bb_y0:bb_y1 + 1, bb_x0:bb_x1 + 1]
        # get the boolean sub image
        sub_img = img[bb_y0:bb_y1 + 1, bb_x0:bb_x1 + 1]
        # find the contour
        contours, _ = cv2.findContours(sub_img, cv2.RETR_EXTERNAL, cv2.CHAIN_APPROX_SIMPLE)
        # clear the sub image and replace with boundary (contour)
        sub_img[:, :] = 0
        cv2.drawContours(sub_img, contours, -1, (255), 3)
        # perform sweeping algorithm
        distances, points = sweeping(sub_img, cx - bb_x0, cy - bb_y0)
        # smooth the resulting series
        distances_smooth = smooth_series(distances, 5, sigma=1.0)
        # get the index with the smallest value (to be used as offset for start point of plot)
        lowest_index = np.argmin(distances_smooth)
        # shift the distance and point arrays by the offset
        shifted_distances = distances[lowest_index:]
        shifted_points = points[lowest_index:]
        shifted_distances.extend(distances[:lowest_index])
        shifted_points.extend(points[:lowest_index])
        # smooth the shifted distances
        new_distances = smooth_series(shifted_distances, 5, sigma=1.0)

        d = np.zeros((len(new_distances)), np.int64)
        for i in range(len(new_distances)):
            d[i] = new_distances[i]
        peaks = find_peaks(d)

        scatter_x = []
        scatter_y = []
        for i in range(len(peaks[0])):
            scatter_x.append(peaks[0][i])
            scatter_y.append(peaks[1][i])

        peak_counter = 0
        for peak_index in peaks[0]:
            text = str(peak_counter + 1)
            # real_peak_index = peak_index + lowest_index
            (ix, iy) = shifted_points[peak_index]
            ix += bb_x0
            iy += bb_y0
            cv2.line(out_img, (int(cx), int(cy)), (int(ix), int(iy)), (255, 0, 0), 2)
            (text_w, text_h), baseline = cv2.getTextSize(text, font, font_size, font_thickness)
            # compute the text offsets (x, y)
            tx = int(round(ix - (text_w / 2.0)))
            ty = int(round(iy + (text_h / 2.0)))
            # render the text
            cv2.circle(out_img, (ix, iy), 10, (255, 255, 0), -1)
            cv2.circle(out_img, (ix, iy), 10, (255, 0, 0), 1)
            # cv2.putText(out_img, text, (tx, ty), font, font_size, (255, 0, 0), font_thickness)
            peak_counter += 1
        return out_img, len(peaks[0])
    except:
        out_img[bb_y0:bb_y1 + 1, bb_x0:bb_x1 + 1] = (0, 0, 0)
        return out_img, 0
```

In [18]:

```
#STEP 4: Start to loop through the images   
# The following libraries are used for image processing
from skimage import color
from skimage import filters
from skimage import img_as_float, img_as_ubyte
from skimage import feature
from skimage import exposure
from skimage.transform import rescale
from skimage.morphology import skeletonize
from skimage import measure
from skimage.measure import label, find_contours
from skimage.measure import regionprops
from skimage.morphology import dilation, erosion, remove_small_objects
from skimage.morphology import disk, remove_small_holes
from skimage.morphology import convex_hull_image
import matplotlib.patches as mpatches
from skimage.draw import circle


##STEP 4.1: Set up a result folder to contain the processing results  
# pattern match jpg and png files, make sure images are .jpg, .jpeg, or .png
if sys.platform == 'win32':
    # windows codes
    imageTypes = ('*.jpg', '*.jpeg', '*.png') # the tuple of jpeg and png file types
elif sys.platform == 'darwin':
    # mac codes
    imageTypes = ('*.jpg', '*.JPG', '*.jpeg', '*.JPEG', '*.png', '*.PNG') # the tuple of jpeg and png file types
else:
    # other code here (linux)
    imageTypes = ('*.jpg', '*.JPG', '*.jpeg', '*.JPEG', '*.png', '*.PNG') 

ImgDirectory = workingRootFolder
ImageFiles = []
# Only select jpg related images 
for imgType in imageTypes:
    ImageFiles.extend(glob.glob(ImgDirectory + imgType))    
# Sort the image files based on their create dates 
ImageFiles.sort(key=str.lower) # changed from os.path.getmtime

# Loop based on the index of image files
##STEP 4.2: Create an empty csv file to save analysis results 
Leaf_file = open(os.path.join(Result_Directory, (time.strftime("%Y-%m-%d") + "_LeafMeasure.csv")), 'wb')
result_List = ["Img_name", "EXP_Date", "Genotype_Treatment","Tray_No","Pot_ID", "Pot_X", "Pot_Y", 
               "Projected_LeafArea(mm^2)", "Leaf_perimeter(mm)", "Canopy_Length(mm)", "Canopy_Width(mm)", 
               "Stockiness(%)", "Leaf_CanopySize(mm^2)", "Leaf_Compactness(%)", 
               "Large_Leaf_No", "Leaf_TotalNo", "Greenness(0-255)", "pix2mm2_ratio"]
# Close the file object as the format of the csv is finished
write_line = csv.writer(Leaf_file, delimiter=',', quoting=csv.QUOTE_NONE)
write_line.writerow(result_List)
Leaf_file.close()   
print "A CSV file called \"%s\" is created in: \n\t%s" %(time.strftime("%Y-%m-%d")+"_LeafArea.csv",Result_Directory)
```

```
A CSV file called "2017-06-21_LeafArea.csv" is created in: 
	/Users/zhouj/Documents/My papers/LeafSeg/Publication Testing/Testing/Processed_2017-6-21
```

In [19]:

```
##STEP 4.3: Batch process the image series
# Set up counters for processing stats
pass_count, fail_count = (0, 0)

# Loop through images in the selected directory
for tmp_IMG_File in ImageFiles:
    # An image used for analysis 
    # pattern match jpg or png files, make sure images are .jpg, .jpeg or .png
    Image_FullName = tmp_IMG_File.split(Platform_Delimiter)[-1]
    # Other parts of an image name    
    ImageName_Length = len(Image_FullName)
    ImageDirectory = tmp_IMG_File[:(ImageName_Length * -1)]
    ImageName = Image_FullName[:-4]
    print "Start reading image: ", ImageName
    
    #***Step_4.3.1***# 
    # Buffer the image file to the memory
    img_tmp = io.imread(tmp_IMG_File)
    Resize_Ratio = 1.0/(img_tmp.shape[0]/1024.0) # dynamically transfer the original resolution 
    image_resized = img_as_ubyte(rescale(img_tmp.copy(), Resize_Ratio)) 
    # The length (y-axis) of the image has been transferred to 1024 pixels
    # Standardise the image size to improve processing efficiency and accuracy
    # Set up a blank image for carrying following image objects in the process
    Blank_Img = np.zeros((image_resized.shape[0], image_resized.shape[1]), dtype=np.uint8)
    # Buffer only one blak image in the memory using numpy 

    # Start to process the image file 
    try: # try...except is used to handle excpetional cases 
        #***Step_4.3.2***# Detect Red reference points on the image 
        # The reference points mask is only generated for presentation purpose
        Ref_Point_Array_Ref, Ref_Point_Areas, RefPoint_image = RefPoints(image_resized, Blank_Img)

        #***Step_4.3.3***# Find the ratio of pixels in the detected red circular reference points
        ## radius of the red sticker used in the experiment is 4mm
        #..! This value shall be input from the GUI
        pix2mm_ratio = convert_pixels_to_mm(Ref_Point_Areas, 4) 
          
        #***Step_4.3.4***# Perspective Transformation based on reference points 
        Transformed_img, New_Ref_Points_Array = PerspectiveTrans_2D(image_resized, Ref_Point_Array_Ref)
        
        #***Step_4.3.5***# Generate segmented pot sections, for a rows x cols tray
        #..! Hard code in iPython notebook, GUI can capture these parameters 
        #######################################
        row_no = 4; column_no = 6 # or 5 and 8 
        #######################################
        Pot_Image, Pot_Segment_Refine = PotSegmentation(Transformed_img, row_no, column_no, New_Ref_Points_Array)
        
        #***Step_4.3.6***# Generate segmented pot sections, for a rows x cols tray
        # Denoising using OpenCV to smooth the leaf surface so that shadows can be presented 
        # in a linear manner. Skimage functions are too slow for this process 
        Transformed_img_Denoised = cv2.fastNlMeansDenoisingColored(Transformed_img,None,10,10,5,
                                                               searchWindowSize=15)
        dilated_lab_Img, leaf_LAB_img = LAB_Img_Segmentation(Transformed_img_Denoised, pix2mm_ratio)
        # Rescale lab image so that it can be used for leaf intensity check 
        # LAB colour spacing deals with green-related lights in uniform chromaticity diagram 
        # This function is designed for select features from the mature leaves 
        p0, p100 = np.percentile(leaf_LAB_img[:, :, 1], (0, 100))
        leaf_LAB_img_rescale = exposure.rescale_intensity(leaf_LAB_img[:, :, 1], in_range=(p0, p100))

        #***Step_4.3.7***# Use Excessive greenness and kmeans to define pixel clustering 
        # Four groups were set: green leaves, soil/compost, reference points and others such as reflection
        img_EGreen = compute_greenness_img(Transformed_img.copy())
        # Reduce the RGB to excessive greenness image for ML performance
        img_ref = img_EGreen.copy() 
        # Use kmeans to cluster pixel groups
        kmeans_img_final = kmeans_cluster(img_ref, 5) # the value of k can be changed, maximum k = 10  
        kmeans_mask = kmeans_img_final > np.mean(kmeans_img_final)*1.0125 # ONLY Five Pixel Groups, 1.025
        
        #***Step_4.3.8***# Finish finding the leaf mask
        dilated_kmeans_Img = dilation(kmeans_mask, disk(3))
        leaf_mask_final = np.logical_and(dilated_lab_Img, dilated_kmeans_Img) # Leaf ROI          
        leaf_mask_ref_1 = np.logical_and(leaf_mask_final, dilation(kmeans_mask, disk(1)))
        leaf_mask_ref = remove_small_holes(leaf_mask_ref_1, 125) # remove small holes in the mask
        Leaf_size_overall = int(np.sum(leaf_mask_ref) * 0.01) # Calculate 1% of the total leaf area
        
    except IOError:
        print "Error: can\'t find file or read data"
        fail_count += 1      
    except ValueError:
        print('Non-numeric data found in the file.')
        fail_count += 1 
    except ImportError:
        print "Import module(s) cannot be found"  
        fail_count += 1
    # Finish the section for running functions    
    except:
        print("Process failed due to analysis issues, see the log file for more information...")
        print ("\t\t Continuing with the next image!")
        fail_count += 1
        gc.collect()    
        continue     


##STEP 4.4: Quantify traits from the images
    try:
        # Start to extract the quantification          
        if leaf_mask_ref.mean() > 0: # The leaf mask contains information
            #***Step_4.4.1***# Prepare leaf measurements 
            ## Step_4.4.1.1 Prepare lists for carrying measurements 
            pot_array = [] # collect the pot location 
            label_array = [] # Collect labels for objects that have been selected 
            no_Count = 0 # used for counting the iteration       
            
            ## Step_4.4.1.2 Prepare images for carrying processed images
            Final_Leaf_img = Blank_Img.copy() 
            Final_Leaf_Skeleton = Blank_Img.copy()
            Final_Tip_img = Blank_Img.copy()
            Leaf_hull_Final = Blank_Img.copy()
            Leaf_hull_Outline_Final = Blank_Img.copy()
            (Leaf_Region_Width, Leaf_Region_Length) = (0, 0) 
            
            # Prepare images for carry leaf tip detection 
            blank_h, blank_w = Blank_Img.shape[:2]
            Final_Leaf_Sweep_img = np.zeros((blank_h, blank_w, 3), np.uint8)
            hull_region = None
            
            ## Step_4.4.1.3 For segmenting pots, use ndimage to extract pot level features  
            Labelled_Pot_Img, num_features = ndimage.measurements.label(Pot_Segment_Refine)              
            ## Step_4.4.1.4 Prepare the processed image
            fig, (ax1, ax2) = plt.subplots(ncols=2, nrows=1, figsize=(25, 15))
            # The background of the processed image
            ax1.imshow(Transformed_img)             
            ax2.imshow(Transformed_img) 
            
            
            #***Step_4.4.2***# Start to generate leaf measurements 
            # Go through every recognised pot 
            for pot_region in regionprops(Labelled_Pot_Img, cache=True):

                ## Step_4.4.2.1 skip too small or too large objects
                if (pot_region.area > (0.925 * Transformed_img.size/(row_no * column_no))) or (pot_region.area < 
                            (0.075 * Transformed_img.size/(row_no * column_no))):
                    continue # Ignore the pot as to big/small***")
                    
                ## Step_4.4.2.2 Prepare temporary pot image base
                pot_tmp_img = Blank_Img.copy()
                pot_coord = pot_region.coords.astype(int)
                # coordinates based on labelled outlines
                pot_tmp_img[pot_coord[:,0], pot_coord[:,1]] = 1
                # expand the pot region to include leaves growing outside the pot
                kernel = np.ones((18,18),np.uint8) # Use OpenCV to increase the dilation performance
                pot_tmp_img_Ref = cv2.dilate(pot_tmp_img,kernel,iterations = 1)
                
                ## Step_4.4.2.3 Generate temporary leaf image base
                Overlap_Leaf_tmp = Blank_Img.copy()
                # Use leaf_mask_final to leaf level detection, use leaf_mask_ref for leaf area measurement 
                Overlap_Leaf_tmp = np.logical_and(leaf_mask_ref, pot_tmp_img_Ref)
                Overlap_Leaf_tmp_Ref = Blank_Img.copy()
                Leaf_Area_tmp = 0.0

                ## Step_4.4.2.4 Start to trim leaves in every pot
                # Normalised LAB is used as the intensity template for feature seleciton
                Labelled_leaf_Img = measure.label(Overlap_Leaf_tmp)             
                for leaf_region in regionprops(Labelled_leaf_Img, intensity_image=leaf_LAB_img_rescale, cache=True):
                    # Remove stones and other non-green small objects
                    if leaf_region.area <= 25: # the resolution is fixed 
                        continue
                    Intensity_Diff = leaf_region.max_intensity - leaf_region.mean_intensity  
                    if Intensity_Diff< 0.15 and leaf_region.area < 225: # Deviation from the scaled difference, 
                        continue        
                    # After rescaling, the most green part has a value -1, 1SD round to 65% -> 35%
                    if leaf_region.mean_intensity > -0.3 or leaf_region.min_intensity > -0.6: 
                        # the LAB has been rescaled between -1.0 and 0 for presenting greenness
                        continue
                    # The position of the leaf object    
                    if leaf_region.centroid[0]<pot_region.bbox[0]*1.025 or leaf_region.centroid[0]>pot_region.bbox[2]*0.975:
                        continue # Column, y-axis
                    if leaf_region.centroid[1]<pot_region.bbox[1]*1.025 or leaf_region.centroid[1]>pot_region.bbox[3]*0.975:
                        continue # Row, x-axis   
                    leaf_contrast = Intensity_Diff*1.0/(leaf_region.max_intensity+leaf_region.min_intensity)
                    # Remove based on contrast levels and intensity values
                    if (leaf_region.mean_intensity>-0.525 or leaf_contrast>-0.185) and leaf_region.area<pix2mm_ratio*pix2mm_ratio*10: 
                        #very small leaves 
                        continue
                    if leaf_contrast > -0.125 and leaf_region.area < pix2mm_ratio*pix2mm_ratio*30: #30 mm^2 
                        #middle size leaves 
                        continue
                    if leaf_contrast>-0.195 and leaf_region.mean_intensity>-0.525 and leaf_region.area>=pix2mm_ratio*pix2mm_ratio*30:
                        # large leaves 
                        continue    
                    #print (no_Count+1), ': ', leaf_contrast, " - ", Intensity_Diff, " - ", leaf_region.area
                    #print leaf_region.mean_intensity, "_", Leaf_size_overall*0.15, "_"
                    leaf_coord = leaf_region.coords.astype(int)
                    Overlap_Leaf_tmp_Ref[leaf_coord[:, 0], leaf_coord[:, 1]] = 1  
                # Final refinement   
                Final_Leaf_Clean = remove_small_objects(dilation(leaf_mask_ref, disk(3)), Leaf_size_overall*0.15)
                Overlap_Leaf_tmp_Ref = np.logical_and(Final_Leaf_Clean, Overlap_Leaf_tmp_Ref)
                Overlap_Leaf_tmp_Ref = remove_small_objects(Overlap_Leaf_tmp_Ref, Leaf_size_overall*0.32)
                Final_Leaf_img = np.logical_or(Overlap_Leaf_tmp_Ref, Final_Leaf_img)  
                Leaf_Area_tmp = np.sum(Overlap_Leaf_tmp_Ref)*1.0/(pix2mm_ratio*pix2mm_ratio)
                #print "Finish processing leaf surface in pot %d!" %(no_Count + 1)
                ## Finish trimming leaves

                ## Step_4.4.2.5 Label pots
                no_Count = no_Count + 1
                ax1.text(pot_region.bbox[1]+25, pot_region.bbox[0]+25, '%d' %(no_Count), ha='center', 
                        va='bottom', color = 'lime', size = 16)# altered from +25 and +25
                ax2.text(pot_region.bbox[1]+25, pot_region.bbox[0]+25, '%d' %(no_Count), ha='center', 
                        va='bottom', color = 'lime', size = 16)# altered from +25 and +25
                
                ## Step_4.4.2.6 Extract Convex Hull for leaf objects
                # Add convex hull to the leaf objects  
                Leaf_hull_tmp = Blank_Img.copy()
                Leaf_hull_Outline = Blank_Img.copy()
                if np.sum(Overlap_Leaf_tmp_Ref) > 0: # Plants found in a pot
                    Leaf_hull_tmp = convex_hull_image(Overlap_Leaf_tmp_Ref)   
                    Leaf_hull_Outline = np.logical_xor(Leaf_hull_tmp, erosion(Leaf_hull_tmp, disk(2))) 
                    Leaf_hull_Outline_Final = np.logical_or(Leaf_hull_Outline_Final, Leaf_hull_Outline)
                    Leaf_hull_Final = np.logical_or(Leaf_hull_Final, Leaf_hull_tmp)
                # Leaf canopy
                Hull_area = np.sum(Leaf_hull_tmp)
                # generate a circle to present the centroid of convex hull 
                Leaf_Centroid_TMP = Blank_Img.copy()
                if (np.sum(Leaf_hull_tmp) > 0): # Plant canopy found in a pot
                    # Measure the region properties of the leaf canopy 
                    label_hull_img = label(Leaf_hull_tmp, connectivity=Leaf_hull_tmp.ndim)
                    hull_region = measure.regionprops(label_hull_img, intensity_image=leaf_LAB_img_rescale, cache=True) 
                    # Measure the full length and width of the canopy  
                    Leaf_Region_Width = round(hull_region[0].minor_axis_length, 0)
                    Leaf_Region_Length = round(hull_region[0].major_axis_length, 0)
                    Leaf_Region_Radius = (Leaf_Region_Length * 1.0) / 2
                    # to remove early small leaves, as the calculation is dynamic
                    Leaf_Region_Hull = (2.75*pix2mm_ratio)*4 # diameter is 4 mm, 50% of final size
                    rr, cc = circle(hull_region[0].centroid[0], hull_region[0].centroid[1], Leaf_Region_Hull)
                    Leaf_Centroid_TMP[rr, cc] = 1 # A filled circle is generated 
                else: # an empty canopy region   
                    Leaf_Region_Width = 0
                    Leaf_Region_Length = 0
                    Leaf_Region_Radius = 0
                # Finish detecting leaf canopy  
                # Overlap_Leaf_tmp_Ref
                #print "Finish processing leaf canopy in pot %d!" %(no_Count)
                   
                ## Step_4.4.2.7 Extract skeletons based on the detected leaf regions
                Sk_tmp_img = Blank_Img.copy()
                Large_Leaf_tmp = Blank_Img.copy()
                # Process the image before skeletonisation 
                total_size_leaf = np.sum(Overlap_Leaf_tmp_Ref)
                # Remove 5% small objects, if the trim function still kept some shining soil objects
                Overlap_Leaf_Sk = remove_small_objects(Overlap_Leaf_tmp_Ref, int(total_size_leaf * 0.05))
                # Improve the skeleton detection by filling holes and dilating the leaf masks 
                Overlap_Leaf_Sk_Ref = ndimage.binary_fill_holes(dilation(Overlap_Leaf_Sk, disk(3))).astype(int)
                # Extract the skeleton 
                Sk_tmp_img = skeletonize(Overlap_Leaf_Sk_Ref)
                
                # Find the end points of the skeleton
                Leaf_tip_array = find_end_points(dilation(Sk_tmp_img, disk(2)))
                Leaf_tmp_tip = Blank_Img.copy()
                Leaf_tmp_tip[Leaf_tip_array[:,1], Leaf_tip_array[:,0]] = 1
                # Overlap with the centroid region to remove small leaves
                Large_Leaf_tmp_tip = np.logical_xor(np.logical_and(Leaf_tmp_tip, Leaf_Centroid_TMP), Leaf_tmp_tip)
                # Prepare for the measurement and output processed picture
                Sk_tmp_img = dilation(Sk_tmp_img, disk(1))
                Large_Leaf_tmp_tip = dilation(Large_Leaf_tmp_tip, disk(5))
                # Start to detect leaf numbers in every pot
                Labelled_leaf_Img = measure.label(Large_Leaf_tmp_tip)
                tip_Count = 1
                for leaf_tip_counter in regionprops(Labelled_leaf_Img, cache=True):
                    # add text onto the leaf tips 
                    ax2.text(leaf_tip_counter.bbox[1], leaf_tip_counter.bbox[0], '%d' %(tip_Count), ha='center', 
                        va='center', color = 'yellow', fontweight='bold', size = 12) 
                    tip_Count = tip_Count + 1
                # Finalise the skeleton graph
                Final_Leaf_Skeleton = np.logical_or(Sk_tmp_img, Final_Leaf_Skeleton)
                Final_Tip_img = np.logical_or(Large_Leaf_tmp_tip, Final_Tip_img)
                #print "Finish processing leaf skeleton in pot %d!" %(no_Count)
                ## Finish generating skeleton for leaf measurements 
                
                
                ## Step_4.4.2.8 Leaf detection based on waveform sweeping method
                if hull_region is not None:
                    Final_Leaf_Sweep_img, total_leaf_number = find_leaves(Overlap_Leaf_tmp_Ref, Final_Leaf_Sweep_img, 
                                                                          hull_region[0].centroid[1], 
                                                                          hull_region[0].centroid[0], pot_region)
                else:
                    total_leaf_number = 0 # If not leaf has found in the pot
                ## Finish generating leaf tip measurements 
            
                #***Step_4.4.3***# Append the quantification to the csv file  
                Leaf_file = open(Result_Directory + "/"  + time.strftime("%Y-%m-%d") + "_LeafMeasure.csv", 'a')
                if ImageName.count('_') < 2: # At least two underscores need to be found 
                    # wrong format of the image name 
                    Imaging_Date = ''
                    Genotype_Treatment_tmp = ''
                    Tray_No_tmp = ''                
                elif ImageName.count('_') >= 4: 
                    # Proper annotation 
                    Imaging_Date = ImageName.split("_")[0]
                    Genotype_Treatment_tmp = ImageName.split("_")[1]+"_"+ImageName.split("_")[2]
                    Tray_No_tmp = ImageName.split("_")[4] 
                else:
                    # Format with two-three '_' in the image name  
                    Imaging_Date = ImageName.split("_")[0]
                    Genotype_Treatment_tmp = ''
                    for elem in ImageName.split("_")[1:-2]: 
                        Genotype_Treatment_tmp = Genotype_Treatment_tmp + elem
                    Tray_No_tmp = ImageName.split("_")[-1] 
                
                if Leaf_Area_tmp > 100 and np.sum(Leaf_tmp_tip) > total_leaf_number: 
                    # When projected leaf areas are small, the skeleton method will over count leaf numbers
                    # 100 means 100 mm^2
                    total_leaf_number = np.sum(Leaf_tmp_tip) 
                    # Choose the larger value calculated from the skeleton method and th tip detection method
                    
                if np.sum(Overlap_Leaf_tmp_Ref)<Leaf_size_overall*0.2 and Leaf_Area_tmp > 1000: 
                    # only applying when the leaf area is over 1000 mm^2
                    Leaf_Region_Radius = 0 # not counted for trait analysis 
                    
                # The string to be appended
                if Leaf_Region_Radius > 0: 
                    Leaf_Stockiness = 4.0*math.pi*(np.sum(Overlap_Leaf_tmp_Ref))/math.pow((2*math.pi*Leaf_Region_Radius),2)
                    result_List = [ImageName, Imaging_Date, Genotype_Treatment_tmp, Tray_No_tmp, no_Count, 
                                   int(pot_region.centroid[1]), int(pot_region.centroid[0]), 
                                   round(np.sum(Overlap_Leaf_tmp_Ref)*1.0/(pix2mm_ratio*pix2mm_ratio), 1),
                                   round(measure.perimeter(Overlap_Leaf_tmp_Ref, neighbourhood=4)/pix2mm_ratio, 1),
                                   round(Leaf_Region_Length*1.0/pix2mm_ratio, 1), 
                                   round(Leaf_Region_Width*1.0/pix2mm_ratio, 1), 
                                   round(Leaf_Stockiness * 100, 1),
                                   round(Hull_area*1.0/(pix2mm_ratio*pix2mm_ratio), 1),
                                   round(np.sum(Overlap_Leaf_tmp_Ref)*100.0/Hull_area, 1),
                                   (tip_Count-1), total_leaf_number,
                                   round(abs(hull_region[0].mean_intensity*1.125)*255.0, 1), round(pix2mm_ratio, 1)
                                   ]
                else: # No leaf found in the pot
                    result_List = [ImageName, Imaging_Date, Genotype_Treatment_tmp, Tray_No_tmp, no_Count, 
                                   int(pot_region.centroid[1]), int(pot_region.centroid[0]), 
                                   0.0, 0.0, 0.0, 0.0, 0.000, 0.0, 0.0, 0, 0,
                                   0.0, round(pix2mm_ratio, 1)
                                   ]
                    
                write_line = csv.writer(Leaf_file, delimiter=',', quoting=csv.QUOTE_NONE)
                write_line.writerow(result_List)
                Leaf_file.close()   
                print "\tFinish exporting leaf measurements in pot %d!" %(no_Count)
                #Finish exporting to the csv file for leaf measurements

                
                
##STEP 5: Export processed images
            ## Step_5.1 Generate contour images for leaf outlines and pot ID
            contours_image = np.logical_and(Final_Leaf_Clean, dilation(leaf_mask_ref, disk(0))) 
            contours2 = find_contours(Leaf_hull_Outline_Final, 0.9)
            for n2, contour2 in enumerate(contours2):
                ax1.plot(contour2[:, 1], contour2[:, 0], linewidth=2, color='yellow')
            tip_contours = find_contours(Final_Tip_img, 0.9)   
            for n3, contour3 in enumerate(tip_contours):
                ax2.plot(contour3[:, 1], contour3[:, 0], linewidth=9, color='red', fillstyle = 'full')
            
            ax2.set_title('Large Leaf Detection', fontsize=12) 
            ax2.axis([-25, Final_Leaf_img.shape[1]+25, Final_Leaf_img.shape[0]+25, -25])  
            # Prepare the processed figure     
            ax1.set_title('Leaf Canopy', fontsize=12) 
            ax1.axis([-25, Final_Leaf_img.shape[1]+25, Final_Leaf_img.shape[0]+25, -25])    
            plt.tight_layout()
            # The following can handle different platforms 
            fig.savefig(os.path.join(Result_Directory+Platform_Delimiter,
                                     (ImageName+'_05_Leaf_Analysis.jpg')), bbox_inches='tight')
            plt.close(fig) # Close the figure 

            ## Step_5.2 Output processed images - calibration
            # Produce processed images
            fig, (ax1, ax2) = plt.subplots(ncols=2, nrows=1, figsize=(24, 12))
            ax1.set_title('Original Image', fontsize=12) 
            ax1.axis([-25, Final_Leaf_img.shape[1]+25, Final_Leaf_img.shape[0]+25, -25])  
            ax1.imshow(image_resized) # The background of the processed image
            ax2.set_title('Transformed Image', fontsize=12) 
            ax2.imshow(Transformed_img_Denoised) # Transformed image
            # Prepare the processed figure     
            plt.axis([-25, Final_Leaf_img.shape[1]+25, Final_Leaf_img.shape[0]+25, -25])    
            plt.tight_layout()
            # The following can handle different platforms 
            fig.savefig(os.path.join(Result_Directory+Platform_Delimiter,
                                     (ImageName+'_01_Image_Calibration.jpg')), bbox_inches='tight')
            plt.close(fig) # Close the figure 
            
            ## Step_5.3 Output processed images - ML clustering 
            # Produce processed images
            fig, (ax1, ax2) = plt.subplots(ncols=2, nrows=1, figsize=(24, 12))
            ax1.set_title('LAB Colour Space with Green-Red Colour Opponents', fontsize=12) 
            ax1.imshow(leaf_LAB_img[:,:,2]) # The non-linear LAB image
            ax2.set_title('Colour Clustering Groups Using Kmeans', fontsize=12)
            ax2.imshow(kmeans_mask, cmap='rainbow')
            # Prepare the processed figure     
            ax1.axis([-25, Final_Leaf_img.shape[1]+25, Final_Leaf_img.shape[0]+25, -25])   
            ax2.axis([-25, Final_Leaf_img.shape[1]+25, Final_Leaf_img.shape[0]+25, -25])    
            plt.tight_layout()
            # The following can handle different platforms 
            fig.savefig(os.path.join(Result_Directory+Platform_Delimiter,
                                     (ImageName+'_02_Colour_Clustering.jpg')), bbox_inches='tight')
            plt.close(fig) # Close the figure 
            
            ## Step_5.4 Output leaf regions
            # Produce processed images
            fig, (ax1, ax2) = plt.subplots(ncols=2, nrows=1, figsize=(24, 12))
            ax1.imshow(np.logical_and(np.invert(Final_Leaf_img), Pot_Segment_Refine), cmap='gray') 
            # Prepare the processed figure  
            ax1.set_title('Refined Projected Leaf Regions in pots', fontsize=12) 
            ax1.axis([-25, Final_Leaf_img.shape[1]+25, Final_Leaf_img.shape[0]+25, -25])  
            ax2.imshow(np.logical_and(Final_Leaf_img, leaf_mask_ref_1), cmap='gray') 
            ax2.set_title('Selected Leaves for Trait Analysis', fontsize=12) 
            ax2.axis([-25, Final_Leaf_img.shape[1]+25, Final_Leaf_img.shape[0]+25, -25]) 
            plt.tight_layout()
            # The following can handle different platforms 
            fig.savefig(os.path.join(Result_Directory+Platform_Delimiter,
                                     (ImageName+'_04_Refined_Leaf_ROI.jpg')), bbox_inches='tight')
            plt.close(fig) # Close the figure 
            
            ## Step_5.4 Output processed images - Leaf extraction 
            # Produce processed images
            fig, (ax1, ax2) = plt.subplots(ncols=2, nrows=1, figsize=(24, 12))
            # The background of the processed image
            ax2.imshow(Transformed_img_Denoised) # The denoised image
            
            contours1 = find_contours(erosion(remove_small_holes(Final_Leaf_Clean, min_size= 525), disk(3)), 0.9)
            for n1, contour1 in enumerate(contours1):
                ax2.plot(contour1[:, 1], contour1[:, 0], linewidth=2, color='yellow')
            ax2.set_title('Outline of Leaves of Interest', fontsize=12)   
            # Crop leaf for presentation 
            Leaf_Crop = Transformed_img_Denoised.copy()
            for layer in range(Leaf_Crop.shape[-1]):
                Leaf_Crop[np.where(np.invert(erosion(Final_Leaf_Clean, disk(3))))] = 1
            ax1.set_title('Leaf Extraction using Lab and Kmeans', fontsize=12)    
            ax1.imshow(Leaf_Crop) 
            # Prepare the processed figure     
            ax1.axis([-25, Final_Leaf_img.shape[1]+25, Final_Leaf_img.shape[0]+25, -25])   
            ax2.axis([-25, Final_Leaf_img.shape[1]+25, Final_Leaf_img.shape[0]+25, -25])    
            plt.tight_layout()
            # The following can handle different platforms 
            fig.savefig(os.path.join(Result_Directory+Platform_Delimiter,
                                     (ImageName+'_03_Leaf_Extraction.jpg')), bbox_inches='tight')
            plt.close(fig) # Close the figure 
            
            ## Step_5.5 Output leaf skeletons 
            # Produce processed images
            fig, (ax1, ax2) = plt.subplots(ncols=2, nrows=1, figsize=(24, 12))
            Skel_img = Final_Leaf_Skeleton.copy()
            # Add end points to the leaf
            Tip_img = Final_Tip_img.copy()
            Skel_Overall = np.logical_or(Skel_img, Tip_img)
            Skel_Overall = np.ma.masked_where(Skel_Overall == 1, Transformed_img[:,:,1])
            # Prepare the processed figure  
            ax1.imshow(np.logical_xor(Pot_Segment_Refine, np.invert(Skel_Overall)), cmap='gray') 
            ax1.set_title('Leaf Skeleton', fontsize=12) 
            ax1.axis([-25, Final_Leaf_img.shape[1]+25, Final_Leaf_img.shape[0]+25, -25])           
            ax2.imshow(Final_Leaf_Sweep_img)
            ax2.set_title('Leaf Tip Detection', fontsize=12) 
            ax2.axis([-25, Final_Leaf_img.shape[1]+25, Final_Leaf_img.shape[0]+25, -25]) 
            plt.tight_layout()
            # The following can handle different platforms 
            fig.savefig(os.path.join(Result_Directory+Platform_Delimiter,
                                     (ImageName+'_06_Leaf_Detection.jpg')), bbox_inches='tight')
            plt.close(fig) # Close the figure 
            print "\tFinish exporting all processed images!"
            
##STEP 6: Processing stats and garbage collection 
            pass_count += 1
            gc.collect()      
            print "Successfully processed: ", ImageName

        # If an image does not contain any leaf information     
        else:
            print "Error: can\'t find any leaf information!"
            fail_count += 1  
    except:
        # In case we failed in the process 
        print("Process failed due to image analysis issues... Continuing with the next image!")
        fail_count += 1
        gc.collect()    
        # Clear memory
        #del img_tmp, image_resized, Transformed_img, Transformed_img_Denoised
        #del leaf_LAB_img, leaf_LAB_img_rescale, img_EGreen
        #del img_ref, kmeans_img_final, kmeans_mask
        continue   
    
    # One image has been processed   
    gc.collect()  
    # Clear memory for the batch processing 
    #del img_tmp, image_resized, Transformed_img, Transformed_img_Denoised
    #del leaf_LAB_img, leaf_LAB_img_rescale, img_EGreen
    #del img_ref, kmeans_img_final, kmeans_mask
    print("garbage collected")
    # Plot results of the processed image

print "\nFinish the analysis - see results in: ", Result_Directory
print "Processing Summary:" 
print "\tThere were %d images successfully processed, with %d failures" %(pass_count, fail_count)
```

```
Start reading image:  2016-07-11_Ler_22C_Tray_4
```

```
/Users/zhouj/anaconda/lib/python2.7/site-packages/skimage/util/dtype.py:110: UserWarning: Possible precision loss when converting from float64 to uint8
  "%s to %s" % (dtypeobj_in, dtypeobj))
/Users/zhouj/anaconda/lib/python2.7/site-packages/ipykernel/__main__.py:28: RuntimeWarning: invalid value encountered in divide
/Users/zhouj/anaconda/lib/python2.7/site-packages/ipykernel/__main__.py:29: RuntimeWarning: invalid value encountered in divide
/Users/zhouj/anaconda/lib/python2.7/site-packages/ipykernel/__main__.py:30: RuntimeWarning: invalid value encountered in divide
```

```
	Finish exporting leaf measurements in pot 1!
```

```
/Users/zhouj/anaconda/lib/python2.7/site-packages/skimage/morphology/misc.py:122: UserWarning: Only one label was provided to `remove_small_objects`. Did you mean to use a boolean array?
  warn("Only one label was provided to `remove_small_objects`. "
```

```
	Finish exporting leaf measurements in pot 2!
	Finish exporting leaf measurements in pot 3!
	Finish exporting leaf measurements in pot 4!
	Finish exporting leaf measurements in pot 5!
	Finish exporting leaf measurements in pot 6!
	Finish exporting leaf measurements in pot 7!
	Finish exporting leaf measurements in pot 8!
	Finish exporting leaf measurements in pot 9!
	Finish exporting leaf measurements in pot 10!
	Finish exporting leaf measurements in pot 11!
	Finish exporting leaf measurements in pot 12!
	Finish exporting leaf measurements in pot 13!
	Finish exporting leaf measurements in pot 14!
	Finish exporting leaf measurements in pot 15!
	Finish exporting leaf measurements in pot 16!
	Finish exporting leaf measurements in pot 17!
	Finish exporting leaf measurements in pot 18!
	Finish exporting leaf measurements in pot 19!
	Finish exporting leaf measurements in pot 20!
	Finish exporting leaf measurements in pot 21!
	Finish exporting leaf measurements in pot 22!
	Finish exporting leaf measurements in pot 23!
	Finish exporting leaf measurements in pot 24!
	Finish exporting all processed images!
Successfully processed:  2016-07-11_Ler_22C_Tray_4
garbage collected

Finish the analysis - see results in:  /Users/zhouj/Documents/My papers/LeafSeg/Publication Testing/Testing/Processed_2017-6-21
Processing Summary:
	There were 1 images successfully processed, with 0 failures
```

In [20]:

```
###################################################################
#                      END of the algorithm                       #
###################################################################
```

In [ ]:

```

```

In [21]:

```
###################################################################
#                   Explanationa of functions                     #
###################################################################
```

In [26]:

```
"""Function_2 Red Reference Points Detection"""
fig, (ax1, ax2, ax3) = plt.subplots(ncols=3, nrows=1, figsize=(30, 20))
ax1.set_xlabel('Length', fontsize=16)
ax1.set_ylabel('Width', fontsize=16)
ax1.set_title('Original Image', fontsize=16)
ax1.imshow(img_tmp)
ax2.set_xlabel('Length', fontsize=16)
ax2.set_ylabel('Width', fontsize=16)
ax2.set_title('Resized Image', fontsize=16)
ax2.imshow(image_resized)
ax3.set_xlabel('Length', fontsize=16)
ax3.set_ylabel('Width', fontsize=16)
ax3.set_title('Reference Points', fontsize=16)
ax3.imshow(RefPoint_image, cmap = 'gray')
print "Centroids of the red refernce points: ", Ref_Point_Array_Ref
print "Every red reference point contain pixels: ", Ref_Point_Areas
```

```
Centroids of the red refernce points:  [[36, 63], [1492, 53], [50, 960], [1500, 949]]
Every red reference point contain pixels:  [1069, 1132, 1071, 1104]
```

In [27]:

```
"""Function_5 2D Perspective Tansformation"""
fig, (ax1, ax2) = plt.subplots(ncols=2, nrows=1, figsize=(15, 20))
#ax1.set_xlabel('Length', fontsize=16)
#ax1.set_ylabel('Width', fontsize=16)
ax1.set_title('Resized Image', fontsize=16)
ax1.imshow(image_resized)
#ax2.set_xlabel('Length', fontsize=16)
#ax2.set_ylabel('Width', fontsize=16)
ax2.set_title('Perspective Tansformed Image', fontsize=16)
ax2.imshow(Transformed_img, cmap = 'gray')
print "Boundaries of the transformed image: ", New_Ref_Points_Array
```

```
Boundaries of the transformed image:  [[0, 0], [1542, 0], [0, 1024], [1542, 1024]]
```

In [28]:

```
"""Function_6 Generate Pot Regions"""
fig, (ax1, ax2, ax3) = plt.subplots(ncols=3, nrows=1, figsize=(30, 20))
#ax1.set_xlabel('Length', fontsize=16)
#ax1.set_ylabel('Width', fontsize=16)
ax1.set_title('Pot Boundary', fontsize=16)
ax1.imshow(Pot_Image, cmap = 'gray')

#ax2.set_xlabel('Length', fontsize=16)
#ax2.set_ylabel('Width', fontsize=16)
ax2.set_title('Perspective Tansformed Image', fontsize=16)
ax2.imshow(Transformed_img, cmap = 'gray')

#ax3.set_xlabel('Length', fontsize=16)
#ax3.set_ylabel('Width', fontsize=16)
ax3.set_title('Identified Pot Regions', fontsize=16)
# Overlay with resized image
Region_Crop = Transformed_img.copy()
for layer in range(Region_Crop.shape[-1]):
    Region_Crop[np.where(np.invert(Pot_Segment_Refine))] = 1
ax3.imshow(Region_Crop, cmap = 'gray')
```

Out[28]:

```
<matplotlib.image.AxesImage at 0x122fa4890>
```

In [29]:

```
"""Function_7.1 Image Denoise"""
fig, (ax1, ax2) = plt.subplots(ncols=2, nrows=1, figsize=(15, 20))
#ax1.set_xlabel('Length', fontsize=16)
#ax1.set_ylabel('Width', fontsize=16)
ax1.set_title('Transformed Image', fontsize=16)
ax1.imshow(Transformed_img)

#ax2.set_xlabel('Length', fontsize=16)
#ax2.set_ylabel('Width', fontsize=16)
ax2.set_title('Denoised and Rescaled Image', fontsize=16)
ax2.imshow(Transformed_img_Denoised, cmap = 'gray')
```

Out[29]:

```
<matplotlib.image.AxesImage at 0x126d23790>
```

In [30]:

```
"""Function_7.2 Use LAB Colour Space to Detect ROI"""
fig, (ax1, ax2, ax3) = plt.subplots(ncols=3, nrows=1, figsize=(30, 20))
#ax1.set_xlabel('Length', fontsize=16)
#ax1.set_ylabel('Width', fontsize=16)
ax1.set_title('Denoise Image', fontsize=16)
ax1.imshow(Transformed_img_Denoised)

#ax2.set_xlabel('Length', fontsize=16)
#ax2.set_ylabel('Width', fontsize=16)
ax2.set_title('ROI based on LAB Colour Space', fontsize=16)
ax2.imshow(dilated_lab_Img, cmap = 'gray')

#ax3.set_xlabel('Length', fontsize=16)
#ax3.set_ylabel('Width', fontsize=16)
ax3.set_title('Rescaled LAB Image For Feature Selection', fontsize=16)
ax3.imshow(leaf_LAB_img_rescale, cmap = 'gray')
```

Out[30]:

```
<matplotlib.image.AxesImage at 0x12f2efe90>
```

In [31]:

```
"""Function_8.1 Use kmeans Machine Learning to define pixel groups on the denoised image"""
from sklearn.cluster import KMeans

def show_colours(colours, block_size=5):
    img = np.zeros((block_size,block_size*len(colours),3), np.uint8)
    for i in range(len(colours)):
        a = int(block_size * i)
        b = int(block_size * (i+1))
        img[:, a:b] = colours[i]
    return img

# Prepare the RGB image, rescale it to reduce the computation 
image = Transformed_img_Denoised.copy()
image_rescale = img_as_ubyte(rescale(image, 0.25)) # 25% of the original image
# Reshape the image into three R,G,B arrays 
# image_rescale_ary = image_rescale.reshape((image_rescale.shape[0] * image_rescale.shape[1], 3))
image_rescale_ary = flatten_img(image_rescale)

# cluster the pixel intensities in RGB 
clt = KMeans(n_clusters = 8) # Assume we will have 8 colour clusters from the RGB images
clt.fit(image_rescale_ary) # Fit with RGB arrays

# Normalise the centroids detected from kmeans ML
n_groups = len(np.unique(clt.labels_))
numLabels = np.arange(0, n_groups + 1)
(hist, _) = np.histogram(clt.labels_, bins = numLabels)
hist = hist.astype("float")
hist /= hist.sum() # normalisation 

fig.tight_layout()
fig, (ax1, ax2, ax3) = plt.subplots(ncols=3, nrows=1, figsize=(30, 5))
#ax1.set_xlabel('Length', fontsize=16)
#ax1.set_ylabel('Width', fontsize=16)
ax1.set_title('kmeans clustering on an RGB image', fontsize=16)
ax1.plot(clt.cluster_centers_)

# Showing the colours of the k-means cluster centres whose frequency in histogram is picked by the algorithm
cluster_colours = clt.cluster_centers_
cluster_colour_img = show_colours(cluster_colours)
ax2.set_title('Colour groups detected from the image', fontsize=16)
ax2.imshow(cluster_colour_img)

# get the number of pixel groups
ax3.set_title('Colour groups simplified for classification', fontsize=16)
ax3.plot(hist)
ax3.plot(range(0, n_groups), [np.median(hist)] * n_groups, 'r', linestyle='--', linewidth = 2)

selected_clusters = hist >= np.median(hist)
# get the number of pixel groups
pixel_groups = np.sum(selected_clusters)
print 'The number of pixel groups found: ', pixel_groups
```

```
The number of pixel groups found:  4
```

In [32]:

```
"""Function_8.2 Use Excessive greenness and kmeans Machine Learning to define pixel clustering """
fig.tight_layout()

fig, ((ax1, ax2, ax3, ax4)) = plt.subplots(ncols=4, nrows=1, figsize=(40, 30))
#ax1.set_xlabel('Length', fontsize=16)
#ax1.set_ylabel('Width', fontsize=16)
ax1.set_title('Denoise Image', fontsize=16)
ax1.imshow(Transformed_img_Denoised)

#ax2.set_xlabel('Length', fontsize=16)
#ax2.set_ylabel('Width', fontsize=16)
ax2.set_title('Excessive Greenness Image', fontsize=16)
ax2.imshow(img_EGreen, cmap = 'YlGnBu')


#ax3.set_xlabel('Length', fontsize=16)
#ax3.set_ylabel('Width', fontsize=16)
ax3.set_title('Pixel Groups After Kmeans', fontsize=16)
ax3.imshow(kmeans_img_final, cmap='rainbow')

#ax4.set_xlabel('Length', fontsize=16)
#ax4.set_ylabel('Width', fontsize=16)
ax4.set_title('Pixel Groups After Kmeans', fontsize=16)
ax4.imshow(kmeans_mask, cmap = 'gray')
```

Out[32]:

```
<matplotlib.image.AxesImage at 0x126b2b4d0>
```

In [33]:

```
"""Function_8.3 Finalise Mask for Feature Selection"""
fig, (ax1, ax2) = plt.subplots(ncols=2, nrows=1, figsize=(15, 20))
#ax1.set_xlabel('Length', fontsize=16)
#ax1.set_ylabel('Width', fontsize=16)
ax1.set_title('Denoise Image', fontsize=16)
ax1.imshow(Transformed_img_Denoised)

#ax2.set_xlabel('Length', fontsize=16)
#ax2.set_ylabel('Width', fontsize=16)
ax2.set_title('Final ROI Mask', fontsize=16)
ax2.imshow(leaf_mask_ref, cmap = 'gray')
```

Out[33]:

```
<matplotlib.image.AxesImage at 0x12f2a3b50>
```

In [34]:

```
"""Function_9.1 Leaf level measurements - Refine Leaf Regions"""
fig, (ax1, ax2) = plt.subplots(ncols=2, nrows=1, figsize=(15, 20))
#ax1.set_xlabel('Length', fontsize=16)
#ax1.set_ylabel('Width', fontsize=16)
ax1.set_title('Finalised ROI Mask', fontsize=16)
ax1.imshow(dilation(leaf_mask_final, disk(2)), cmap = 'gray')

#ax2.set_xlabel('Length', fontsize=16)
#ax2.set_ylabel('Width', fontsize=16)
ax2.set_title('Finalised Leaf Region Mask', fontsize=16)
ax2.imshow(leaf_mask_ref_1, cmap = 'gray')
```

Out[34]:

```
<matplotlib.image.AxesImage at 0x12d8a2d10>
```

In [35]:

```
"""Function_9.2 Leaf level measurements - Loop Through Every Pot"""
fig, (ax1, ax2, ax3) = plt.subplots(ncols=3, nrows=1, figsize=(30, 20))
#ax1.set_xlabel('Length', fontsize=16)
#ax1.set_ylabel('Width', fontsize=16)
ax1.set_title('Extracted Pot Regions', fontsize=16)
ax1.imshow(Pot_Segment_Refine, cmap = 'gray')

#ax2.set_xlabel('Length', fontsize=16)
#ax2.set_ylabel('Width', fontsize=16)
ax2.set_title('ROI Refined After Selecting Pot Level Leaf Features', fontsize=16)
ax2.imshow(Final_Leaf_img, cmap = 'gray')

ax3.set_title('Finalised Leaf Regions', fontsize=16)
ax3.imshow(Final_Leaf_Clean, cmap = 'gray')
```

Out[35]:

```
<matplotlib.image.AxesImage at 0x12d5b1790>
```

In [36]:

```
"""Function_9.3 Leaf level measurements - Leaf Structure"""
fig, (ax1, ax2) = plt.subplots(ncols=2, nrows=1, figsize=(20, 20))
#ax1.set_xlabel('Length', fontsize=16)
#ax1.set_ylabel('Width', fontsize=16)
ax1.set_title('Skeletonisation to Represent Leaf Structure', fontsize=16)
ax1.imshow(Final_Leaf_Skeleton, cmap = 'gray')

#ax2.set_xlabel('Length', fontsize=16)
#ax2.set_ylabel('Width', fontsize=16)
ax2.set_title('Large Leaf Detection', fontsize=16)
ax2.imshow(np.logical_or(dilation(Final_Tip_img, disk(3)), Pot_Image), cmap = 'gray')
```

Out[36]:

```
<matplotlib.image.AxesImage at 0x12d8ad550>
```

In [37]:

```
"""Function_9.4 Leaf level measurements - Leaf Canopy"""
fig, (ax1, ax2) = plt.subplots(ncols=2, nrows=1, figsize=(20, 20))
#ax1.set_xlabel('Length', fontsize=16)
#ax1.set_ylabel('Width', fontsize=16)
ax1.set_title('Finding Leaf Canopy Region', fontsize=16)
ax1.imshow(np.logical_or(Leaf_hull_Final, Pot_Image), cmap = 'gray')

#ax2.set_xlabel('Length', fontsize=16)
#ax2.set_ylabel('Width', fontsize=16)
ax2.set_title('Calculate Stockiness and Compactness', fontsize=16)
ax2.imshow(np.logical_or(dilation(Leaf_hull_Outline_Final, disk(1)), Final_Leaf_img), cmap = 'gray')
```

Out[37]:

```
<matplotlib.image.AxesImage at 0x131d9a510>
```

In [38]:

```
"""Function_9.5 Leaf level measurements - Leaf Number Detection"""
fig, (ax1, ax2) = plt.subplots(ncols=2, nrows=1, figsize=(20, 20))
#ax1.set_xlabel('Length', fontsize=16)
#ax1.set_ylabel('Width', fontsize=16)
ax1.set_title('Finding Large Leaves', fontsize=16)
ax1.imshow(np.logical_or(np.logical_xor(Pot_Segment_Refine, np.invert(Skel_Overall)), Pot_Image), cmap = 'gray')

#ax2.set_xlabel('Length', fontsize=16)
#ax2.set_ylabel('Width', fontsize=16)
ax2.set_title('Leaf tip detection', fontsize=16)
ax2.imshow(Final_Leaf_Sweep_img, cmap = 'gray')
```

Out[38]:

```
<matplotlib.image.AxesImage at 0x12689db50>
```

In [39]:

```
###################################################################
#                      END of the functions                       #
###################################################################
```

In [ ]:

```

```
