# Supplementary material for: Leaf-GP: an open and automated software application for measuring growth phenotypes for arabidopsis and wheat
Source: Plant Methods. 2017 Dec 22;13:117. doi: 10.1186/s13007-017-0266-3 (PMC5740932; doi:10.1186/s13007-017-0266-3)
Supplement: Supplementary file 2 — Additional file 2. Install manual for Python environment, Anaconda Python distribution and OpenCV-Python binding. [file 13007_2017_266_MOESM2_ESM.docx]

**Additional File 2: Install Python, the Anaconda Python distribution and OpenCV Python Binding**

**1. Install Python releases:**

- Read the beginner’s guide to Python if you are new to the language:

<https://wiki.python.org/moin/BeginnersGuide>

- For Windows users, Python 2 release can be downloaded via:

<https://www.python.org/downloads/windows/>

- For Mac OS users, Python 2 release can be downloaded via:

<https://www.python.org/downloads/mac-osx/>

- Leaf-GP currently only supports Python 2.7.x

**2. Install Anaconda Python distribution:**

- Read the install instruction using the URL: <https://docs.continuum.io/anaconda/install>
- For Windows users, a detailed step-by-step installation guide can be found via:

<https://docs.continuum.io/anaconda/install/windows>

- For Mac OS users, a detailed step-by-step installation guide can be found via:

<https://docs.continuum.io/anaconda/install/mac-os.html>

- An Anaconda Graphical installer can be found via:

<https://www.continuum.io/downloads#macos>

- We recommend users to install the latest Anaconda Python distribution

**3. OpenCV**

- After having installed the Anaconda distribution, users can use **conda** (a package manager application) to install OpenCV (version 2.4.11, required by Leaf-GP).
- To install this package with conda run:

**conda install -c menpo opencv**

- Menpo online package repository can be found via: <https://anaconda.org/menpo/opencv>
- For installing the full-version OpenCV-Python, see the URL:

<http://opencv-python-tutroals.readthedocs.io/en/latest/py_tutorials/py_tutorials.html>
