# Supplementary material for: Leaf-GP: an open and automated software application for measuring growth phenotypes for arabidopsis and wheat
Source: Plant Methods. 2017 Dec 22;13:117. doi: 10.1186/s13007-017-0266-3 (PMC5740932; doi:10.1186/s13007-017-0266-3)

**Additional File 3: Processed Images for *Arabidopsis* Rosette**

**1. Processed images:**

1) **2016/09/16**, Stage No: 1.02, 2 rosette leaves are greater than 1 mm in length


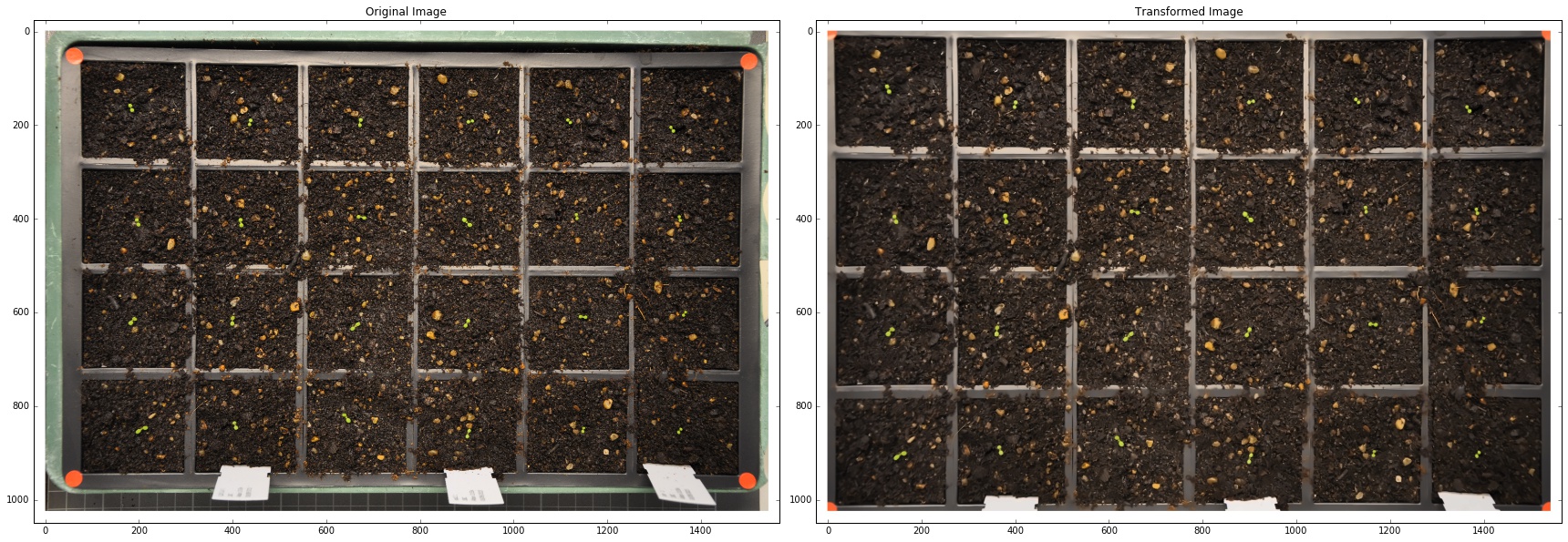

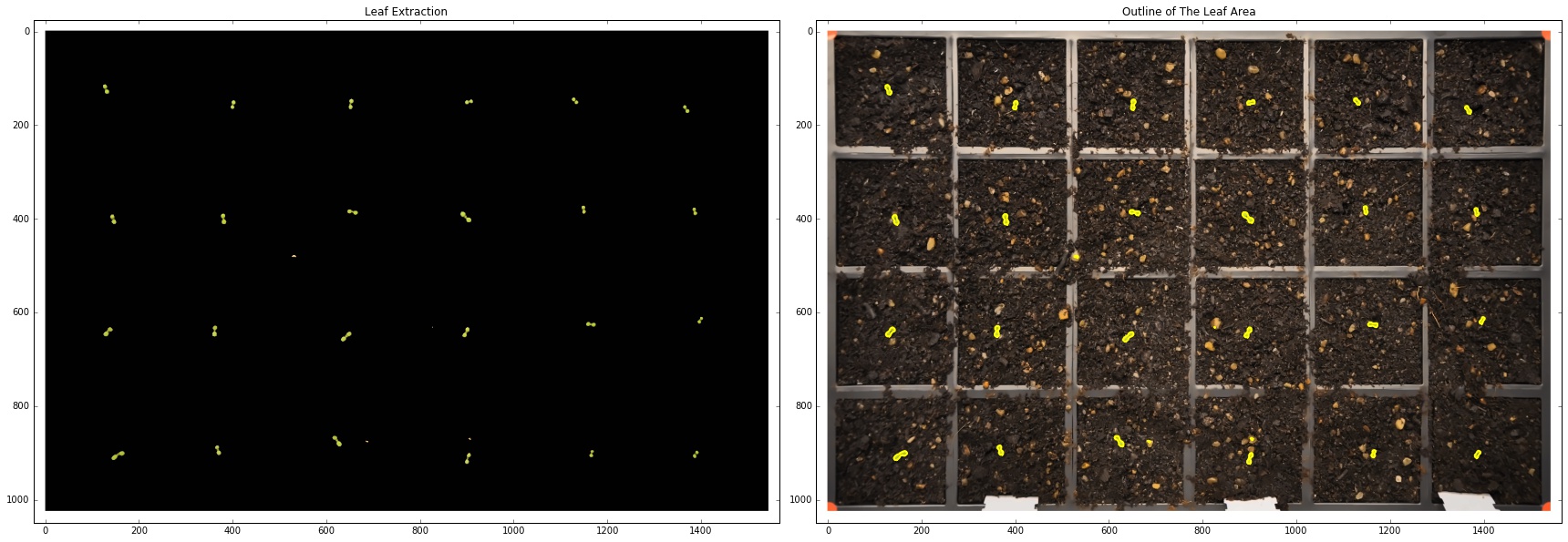

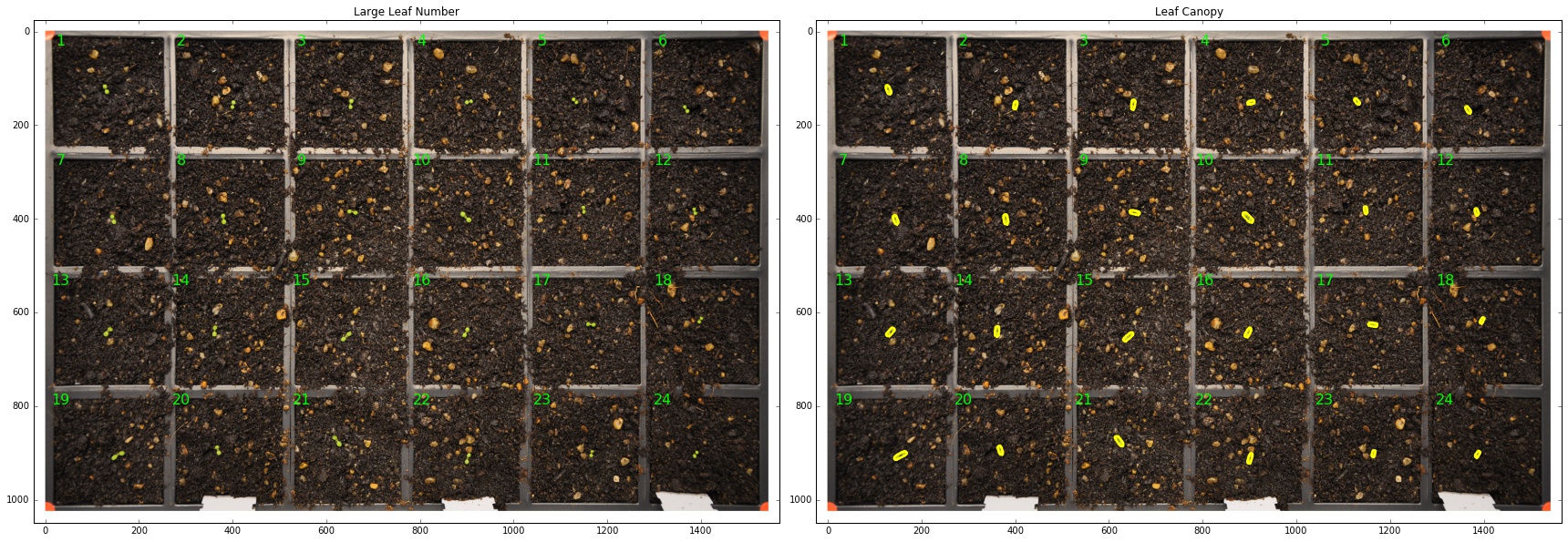

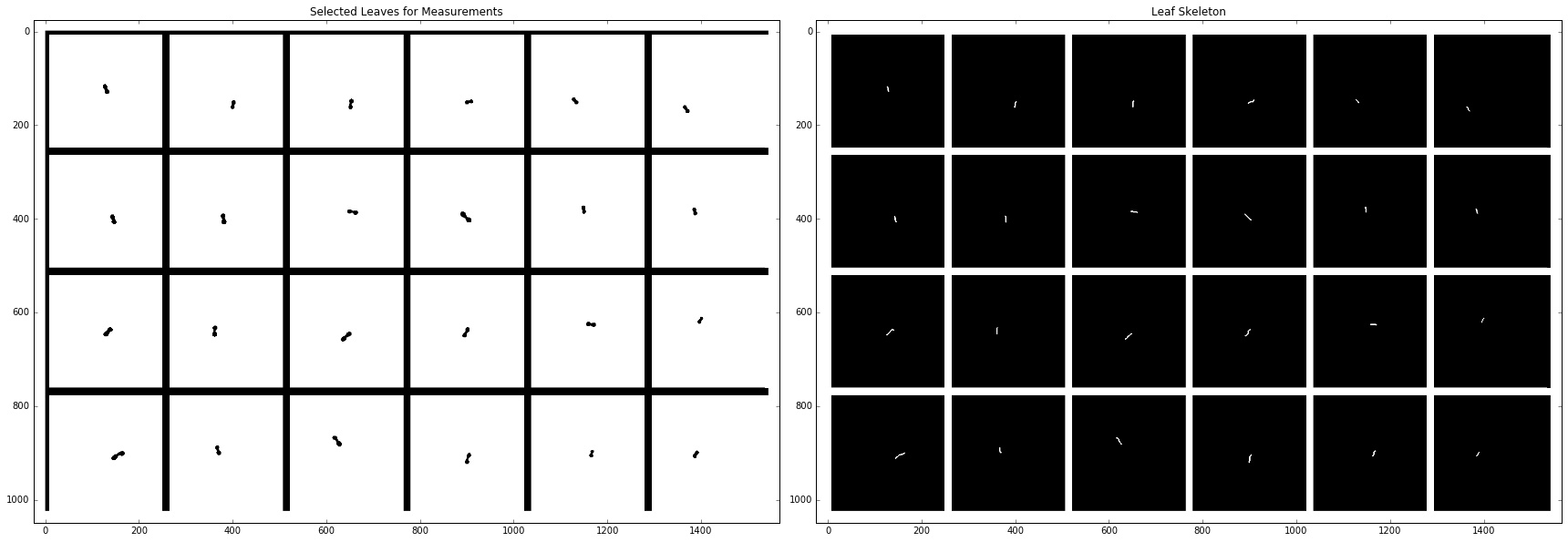


2) **2016/09/21**, stage number: 1.04, 4 rosette leaves are greater than 1mm in length


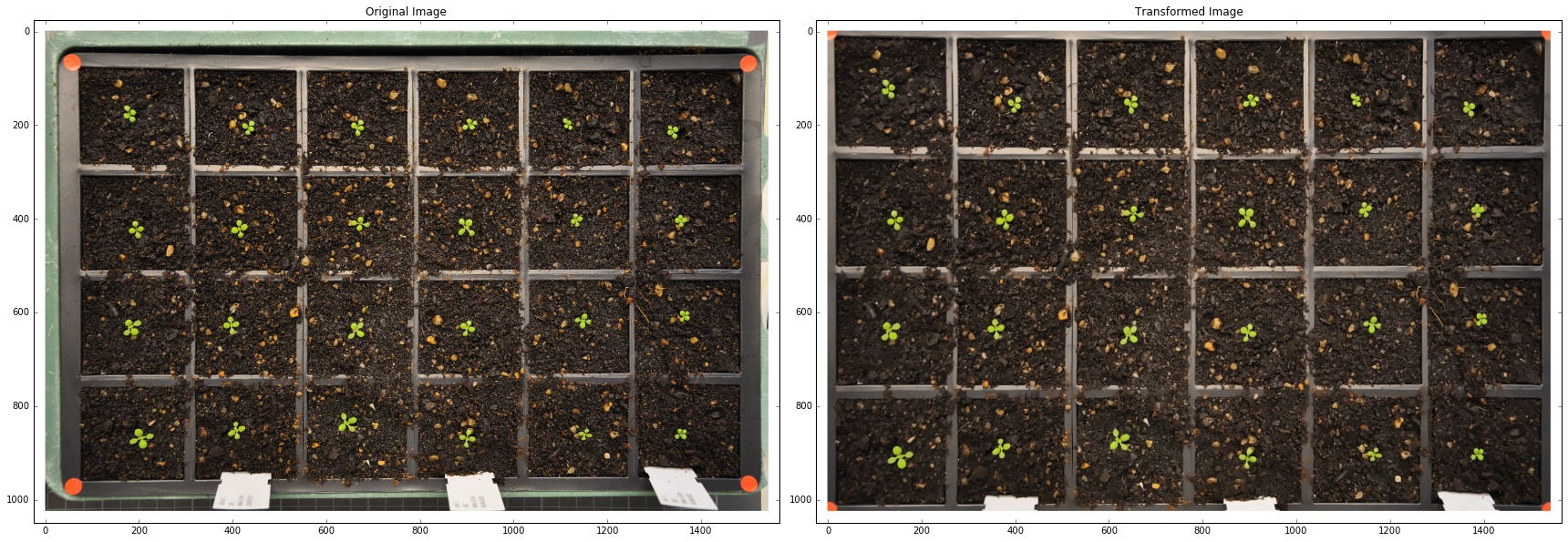

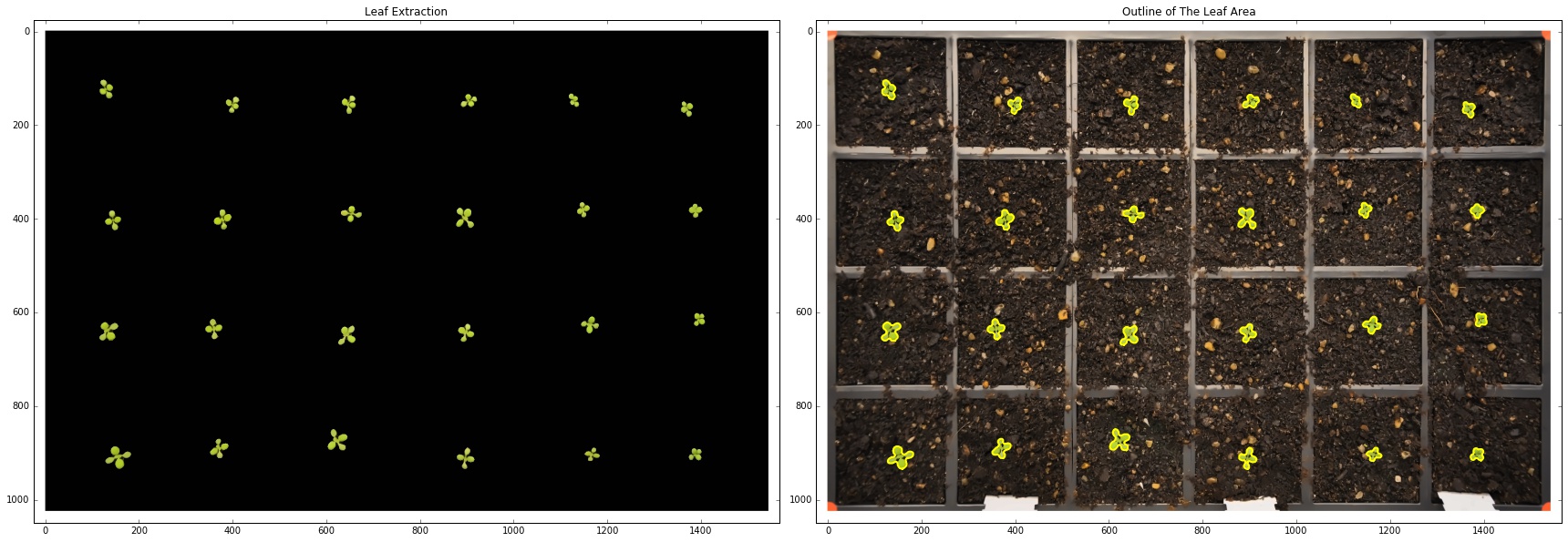

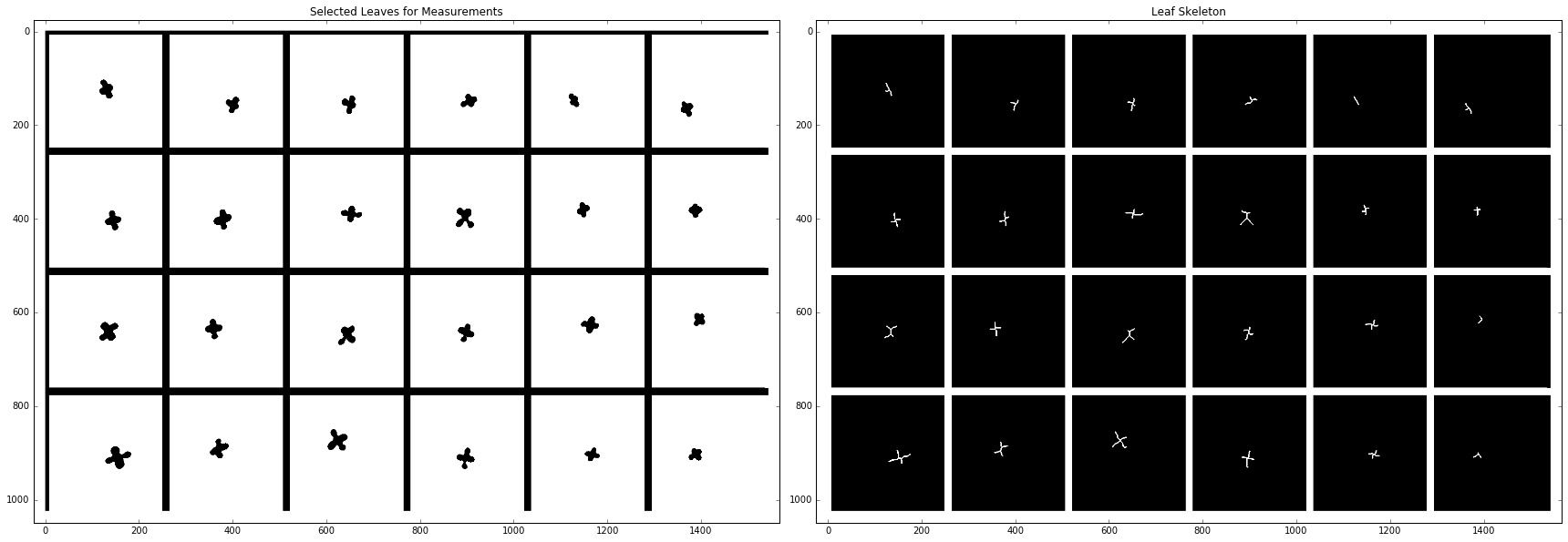

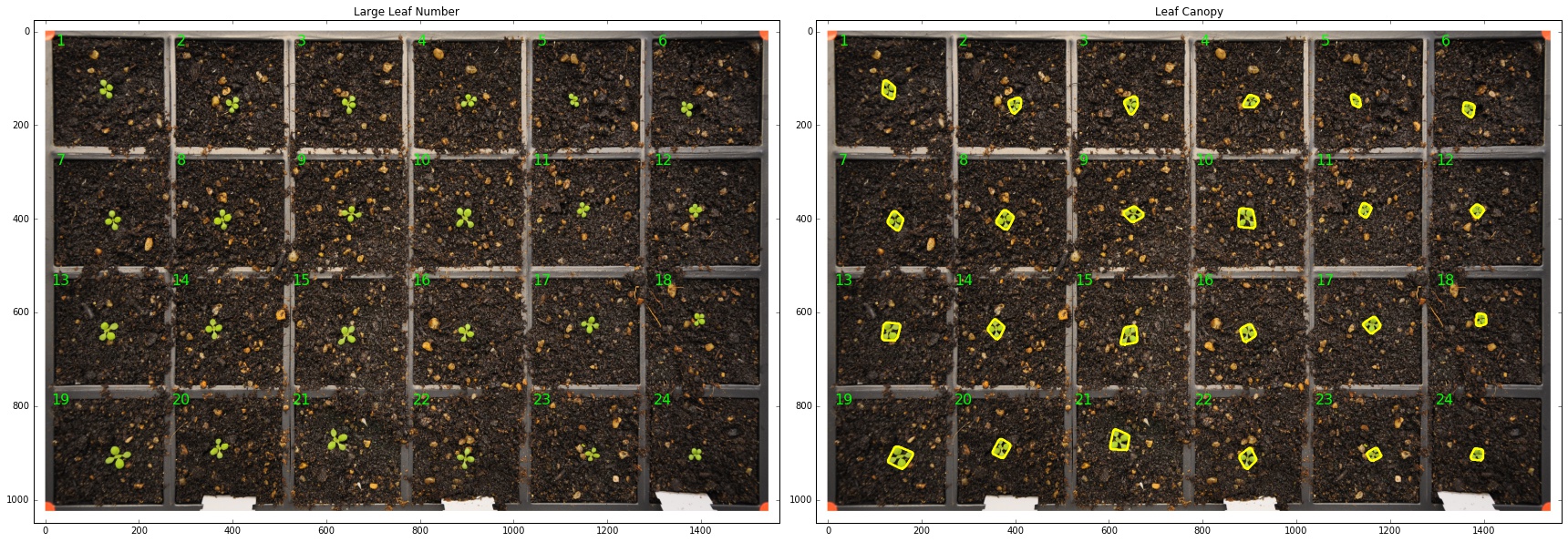


3) **2016/09/28**, stage number: 1.08, 8 rosette leaves are greater than 1mm in length


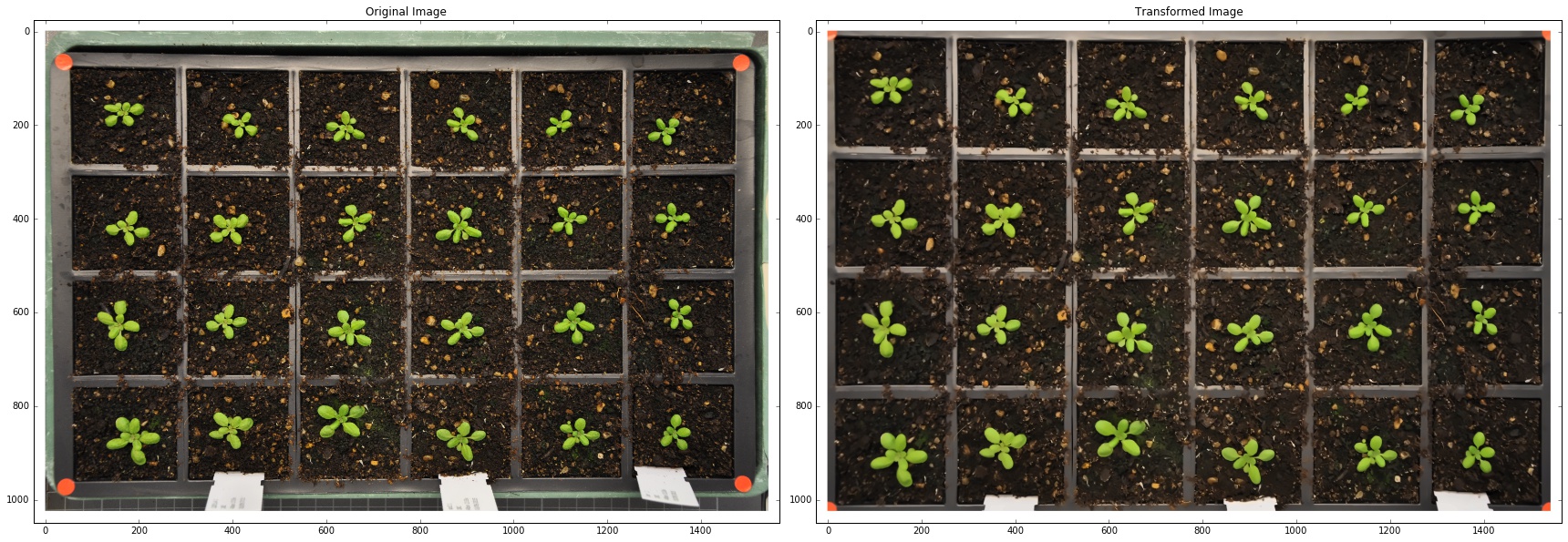

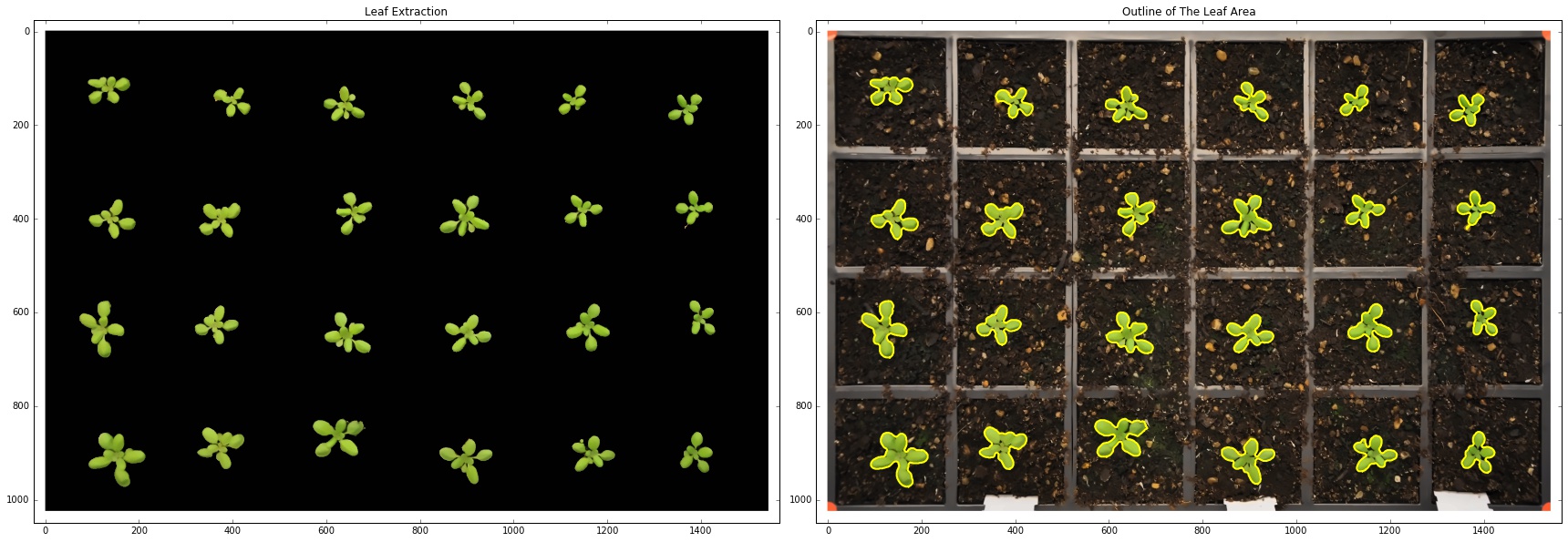

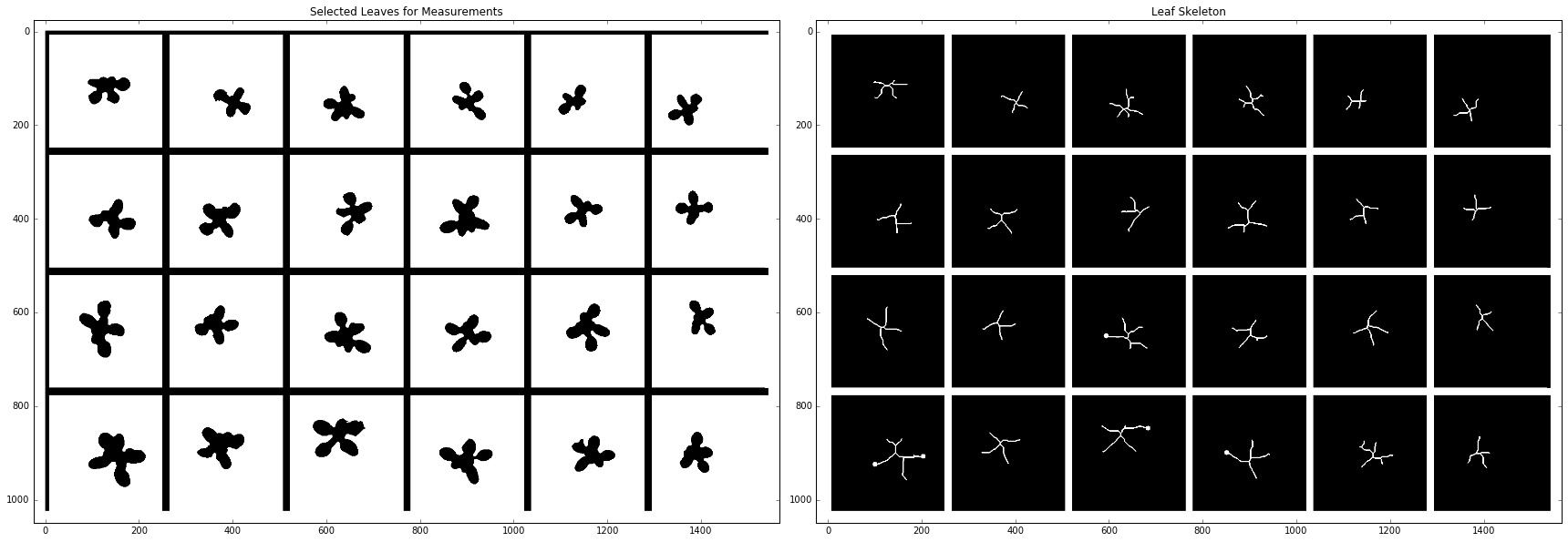

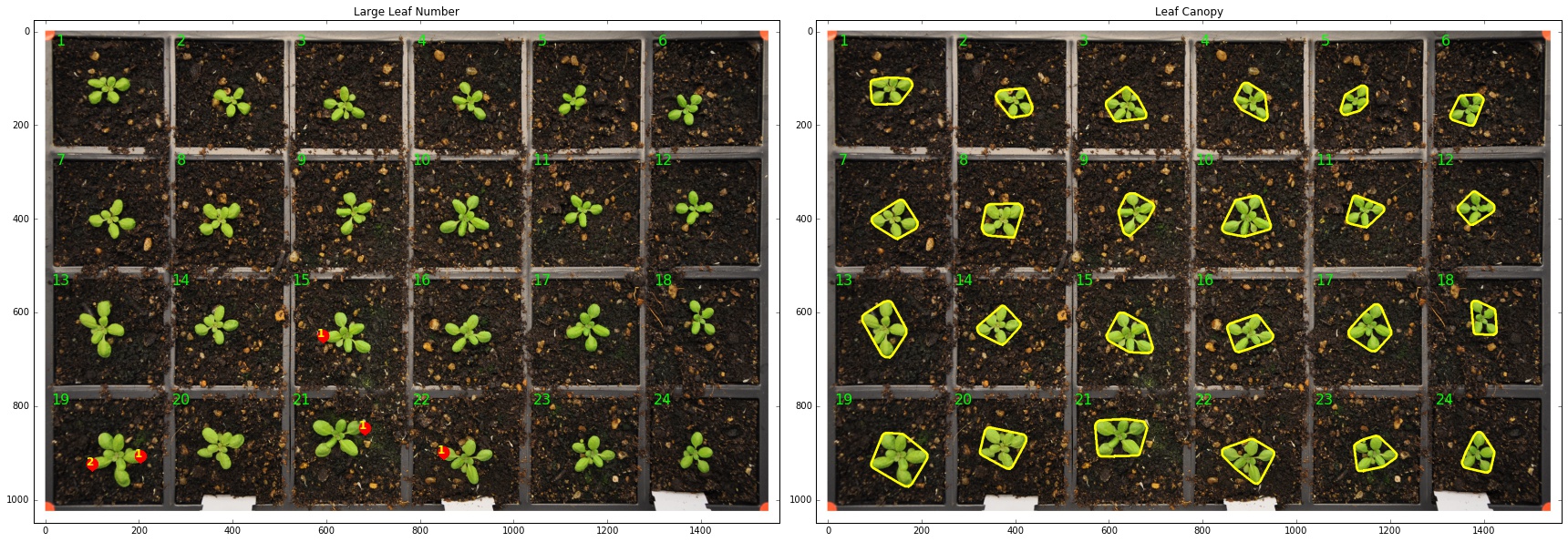


4) **2016/10/02**, stage number: 1.12, 12 rosette leaves are greater than 1mm in length


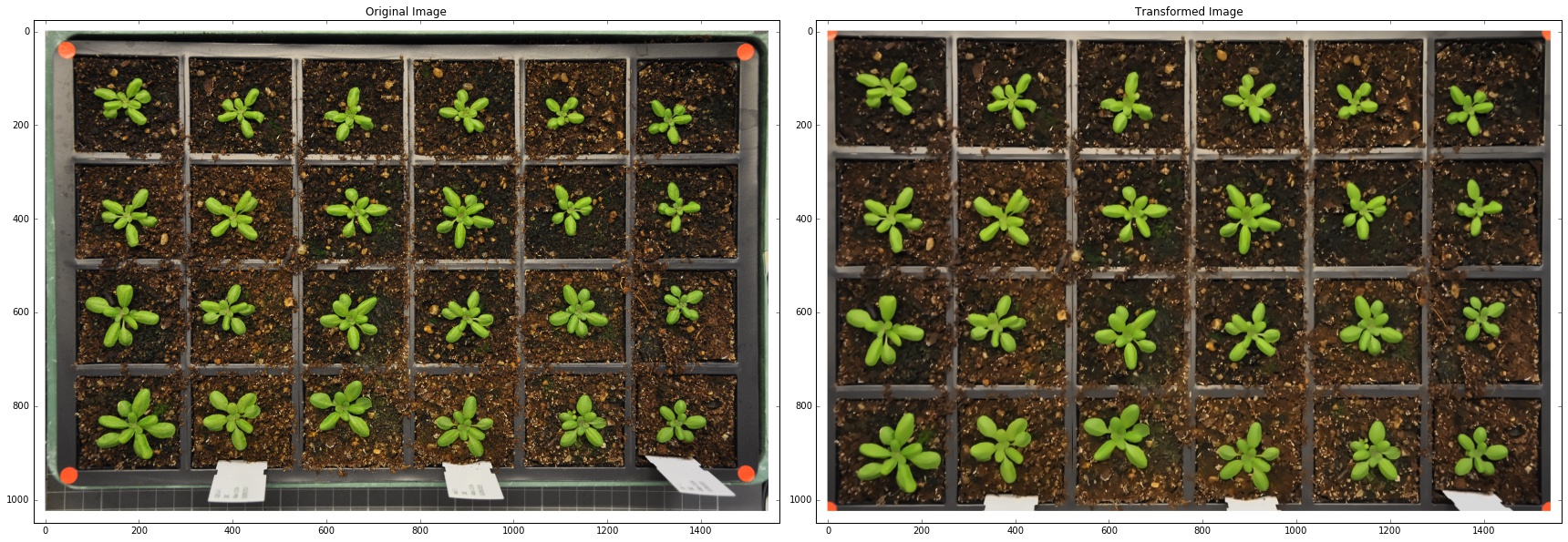

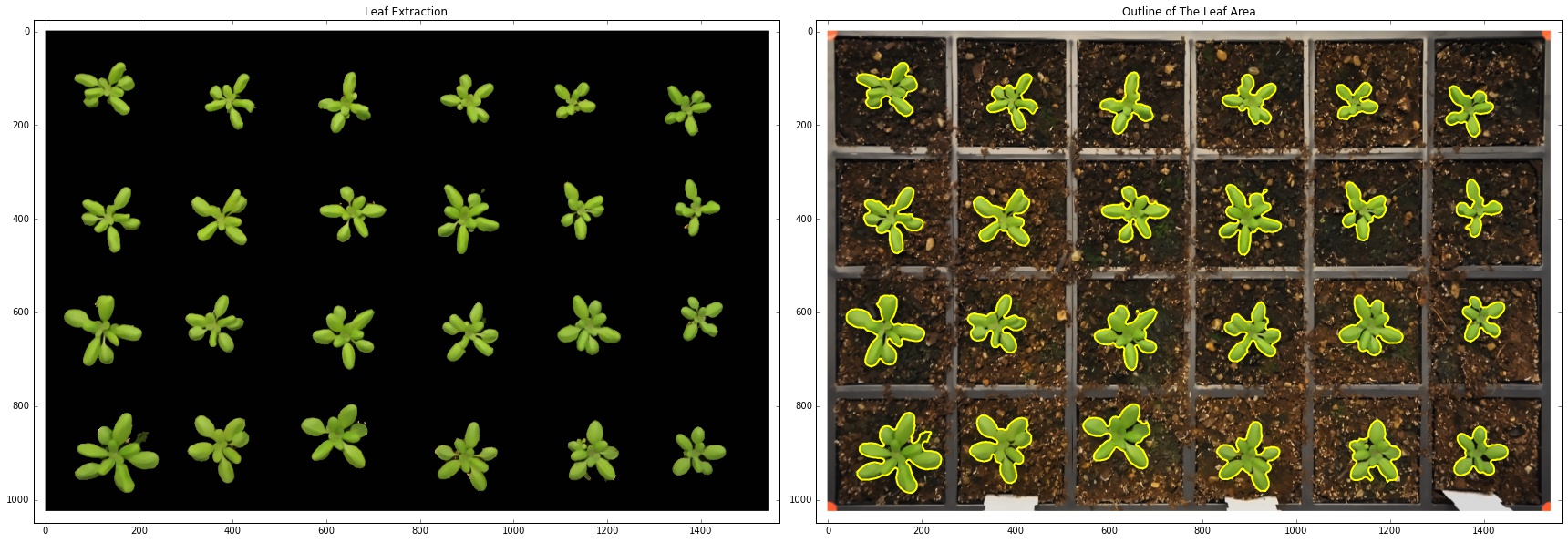

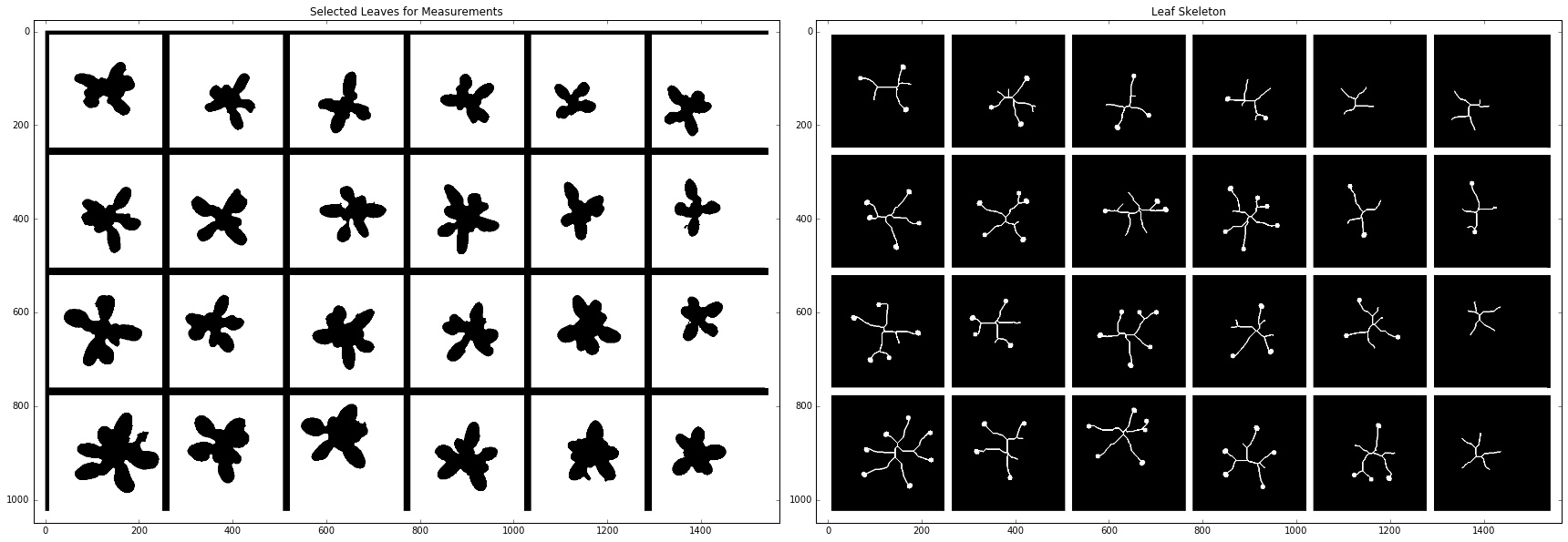

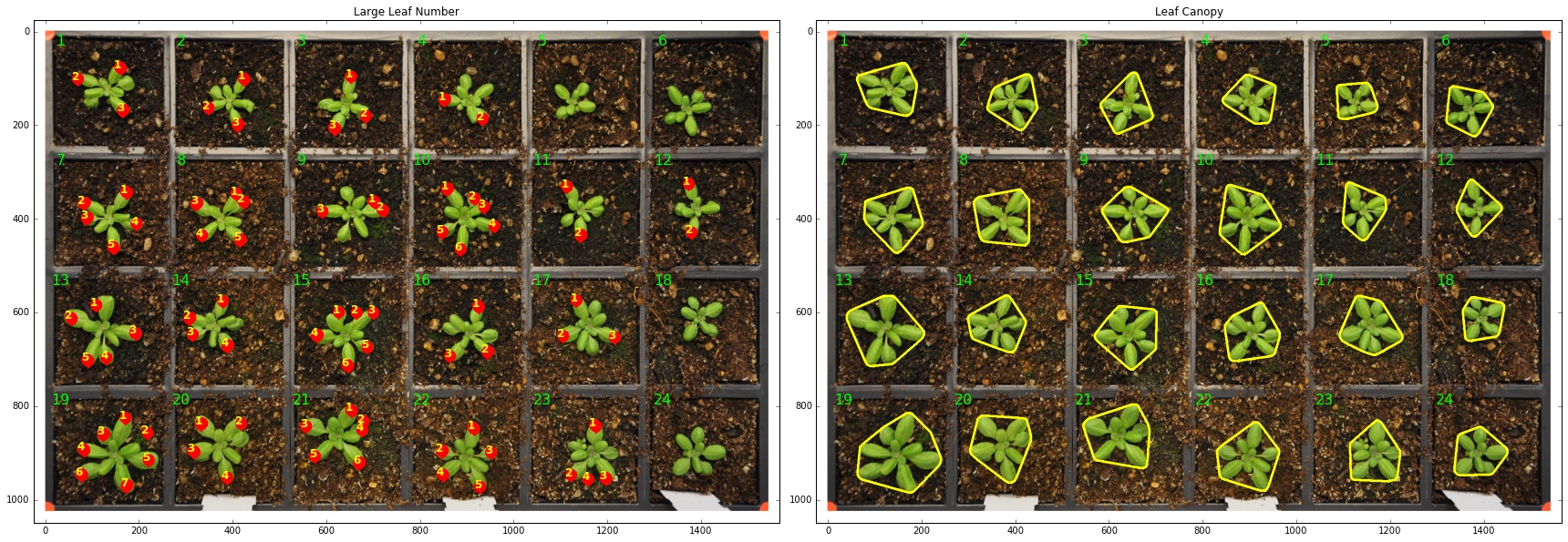


5) **2016/10/07**, stage number: 3.50, Rosette is 50% final size


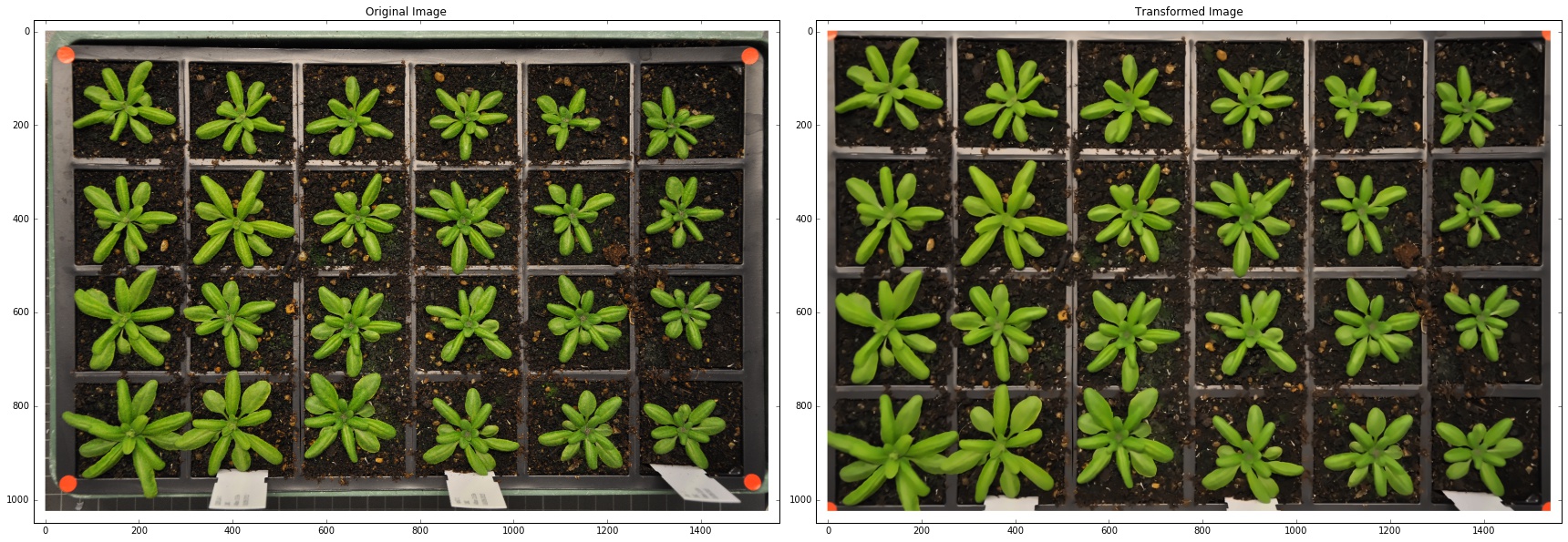

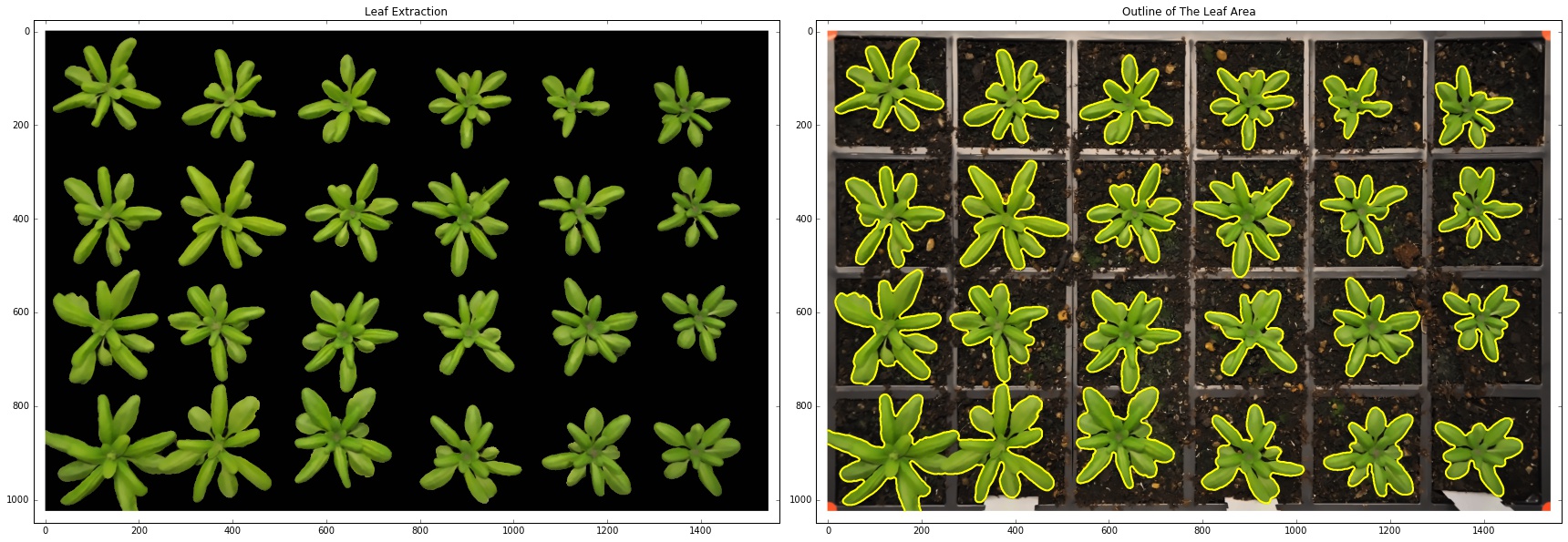

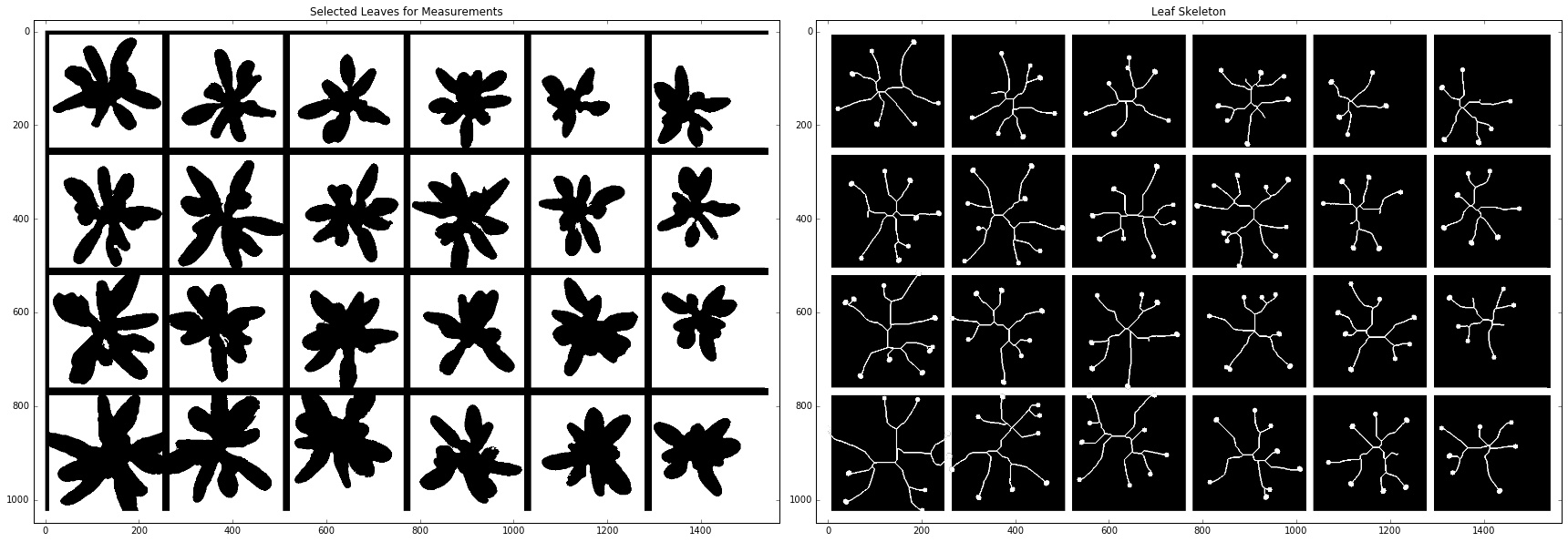

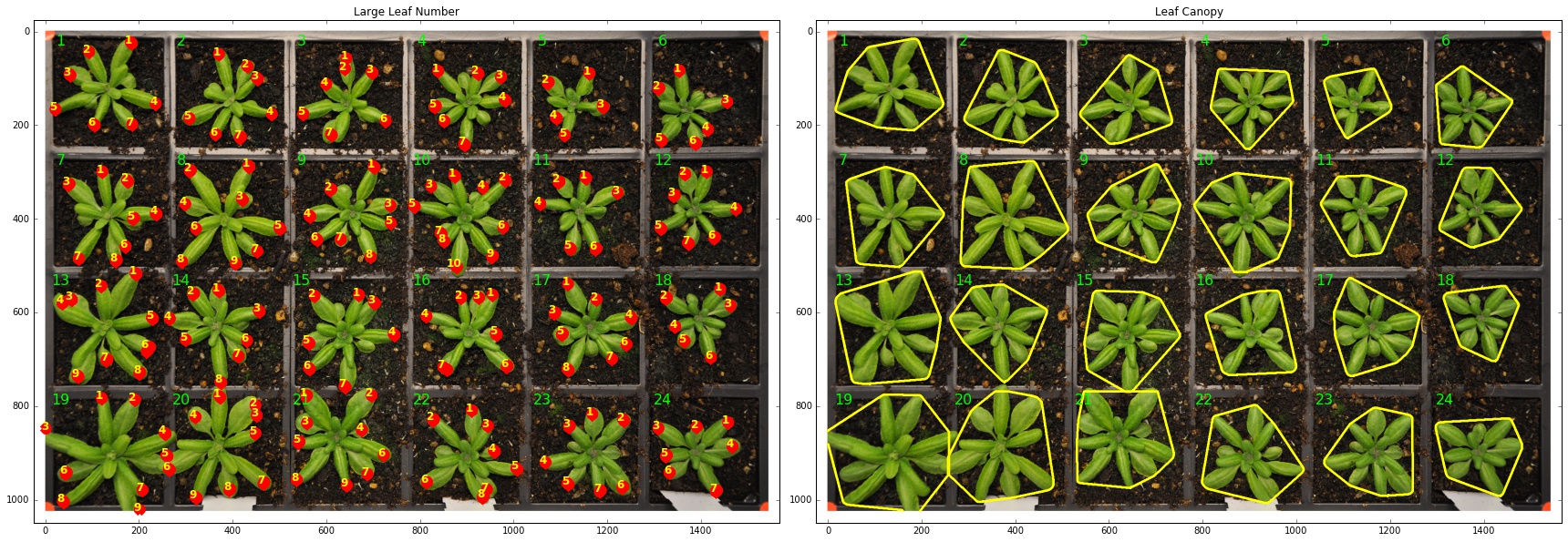


6) **2016/10/13**, stage number: 3.90, Rosette growth is complete.


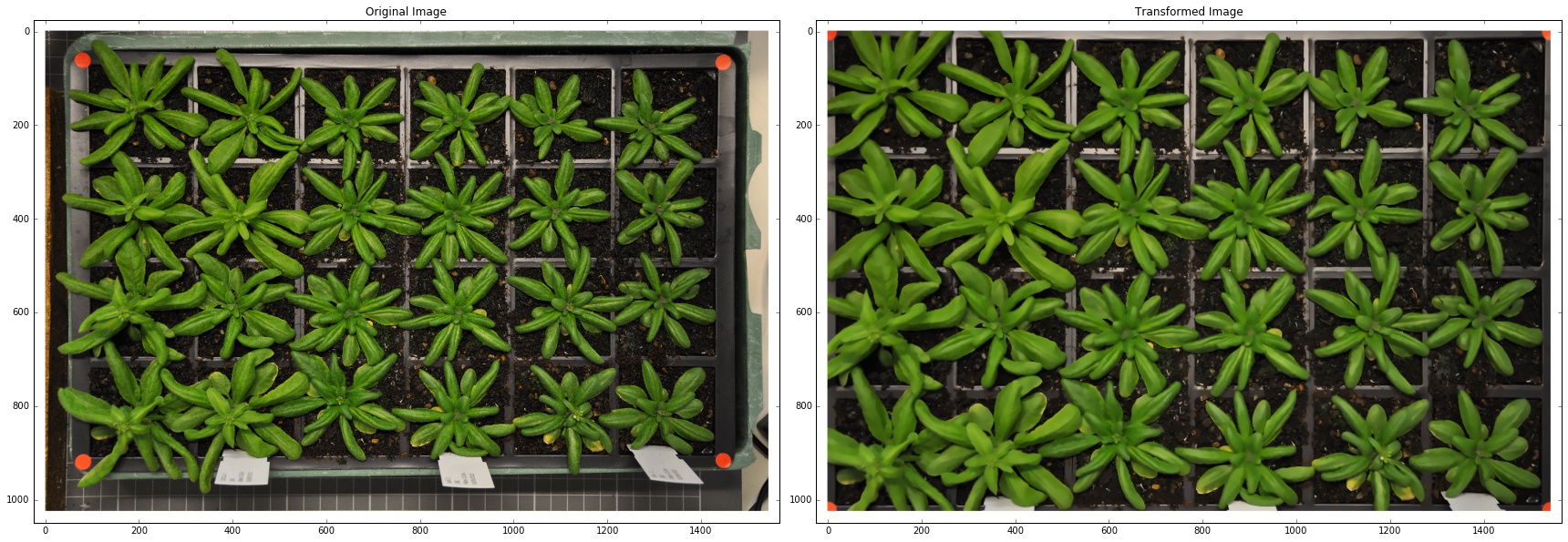

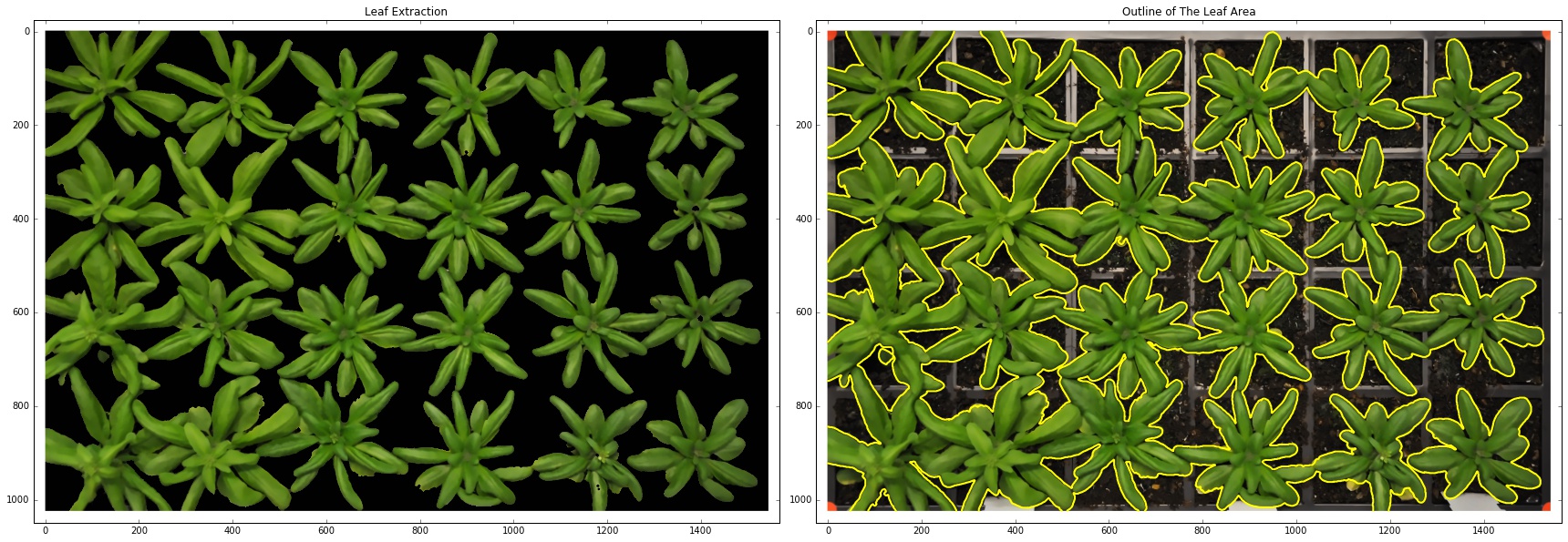

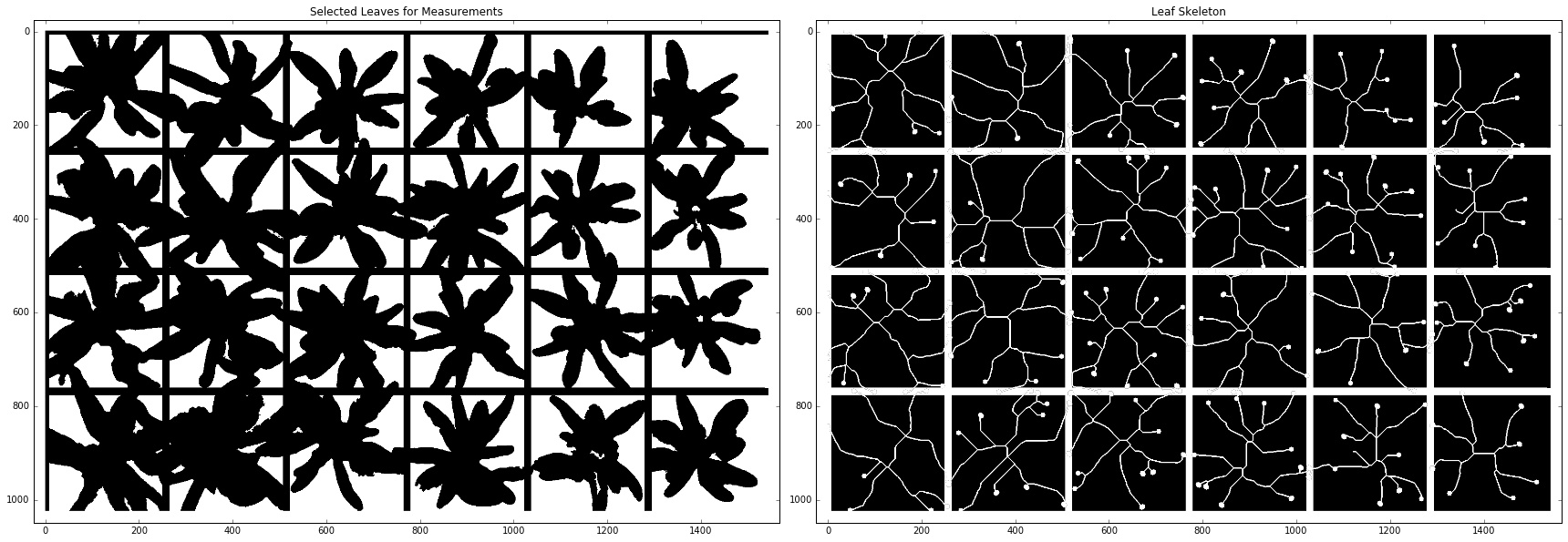

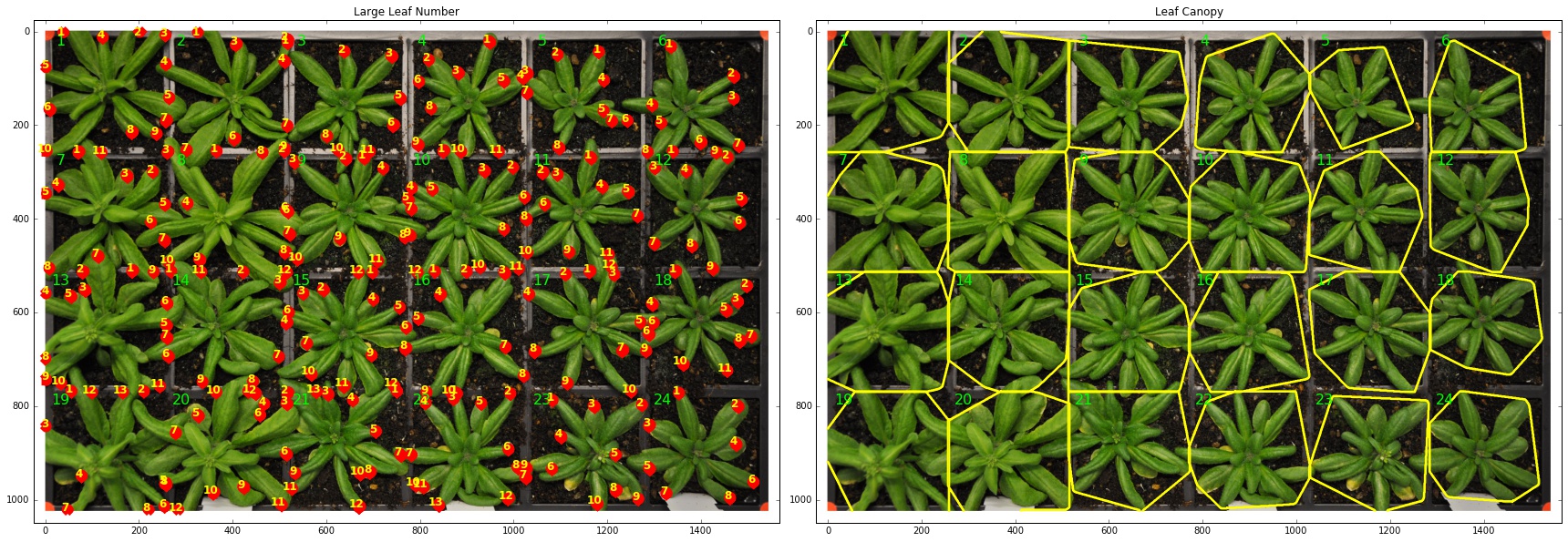

Supplement: Supplementary file 3 — Additional file 3. Processed images of Arabidopsis rosettes at different growth stages. [file 13007_2017_266_MOESM3_ESM.docx]
