# Supplementary material for: Leaf-GP: an open and automated software application for measuring growth phenotypes for arabidopsis and wheat
Source: Plant Methods. 2017 Dec 22;13:117. doi: 10.1186/s13007-017-0266-3 (PMC5740932; doi:10.1186/s13007-017-0266-3)
Supplement: Supplementary file 5 — Additional file 5. The Jupyter Notebook version for plotting and cross-referencing growth traits between experiments. [file 13007_2017_266_MOESM5_ESM.html]

Leaf-GP\_plot\_generator\_V1.0


In [1]:

```
import csv
import matplotlib
import matplotlib.pyplot as plt
from datetime import datetime
import numpy as np
from trait_ids import Trait
```

In [2]:

```
# loads specified csv file and returns a list of dictionaries where
# dictionary keys are csv column headers and values are data values.
# list of dictionaries are ordered as they appear in csv file.
# column headers must be unique and 1st row of csv file must contain column headers.
# attribute values must be able to be represented as floating point values.
# @param csv_filename: the csv file to load
def load_csv(csv_filename):
    # store list of ordered column headers
    column_headers = []
    # store ordered list of row key/value dictionaries
    row_dicts = []
    # store current row number
    row_number = 0
    # open csv file
    with open(csv_filename, 'r') as csvfile:
        # create reader object
        reader = csv.reader(csvfile, delimiter=',', quotechar='|')
        # iterate through the rows of the csv file
        for row in reader:
            if len(row) > 0:
                # if this is the first row (header row)
                if row_number == 0:
                    # iterate through the columns in the header row and add to key set
                    for value in row:
                        column_headers.append(value)
                # otherwise if we are on a data row
                else:
                    # create an empty dictionary for this csv row
                    row_dict = dict()
                    # counter to store which column we are currently processing
                    value_counter = 0
                    # iterate through the columns in the row
                    for value in row:
                        # add key/value pair to dictionary
                        row_dict[column_headers[value_counter]] = value
                        # increment the column counter
                        value_counter += 1
                    # add the row dictionary to the list of dictionaries
                    row_dicts.append(row_dict)
                # move to next row in csv file
                row_number += 1
    # return list of dictionaries
    return row_dicts
```

In [5]:

```
# get date ordered list of values for specified plot id and attribute from list of dictionaries representing csv file
# attribute values must be able to be represented as floating point values
# @param row_data: ordered list of dictionaries (as returned by load_csv function)
# @param experiment_id: the experiment id to return data for (genotype/treatment)
# @param tray_number: the tray number to return data for
# @param plt_id: the plot id to return data for
# @param column_header: the trait attribute id to get data for (column header in csv file)
# @param input_date_format: the date format in the csv file
def get_trait_info(row_data, experiment_id, tray_number, plt_id, column_header, input_date_format="%Y-%m-%d"):
    # create a dictionary where key is row date and value is list of attribute values
    data_map = dict()
    # iterate through the rows of the csv file and add date/attribute pair to dictionary
    for row in row_data:
        # if the experiment id and tray number are the specified experiment id and tray number
        if row[Trait.EXPERIMENT_REF] == experiment_id and float(row[Trait.TRAY_NO]) == float(tray_number):
            # if the current row is showing data for the specified plot id
            if plt_id is None or row[Trait.POT_ID] == plt_id:
                # get the date
                date = row[Trait.EXP_DATE]
                # parse as datetime object
                datetime_obj = datetime.strptime(date, input_date_format)
                # convert to y/m/d format and output as string (so date strings can be ordered)
                date = datetime_obj.strftime('%Y-%m-%d')
                # get the attribute value (convert to floating point representation)
                value = float(row[column_header])
                # do we already have a record for this date in our dictionary?
                if date in data_map.keys():
                    # if so, add the value to the end of the list of values for this date
                    data_map[date].append(value)
                # otherwise, add the date and attribute value to the dictionary
                else:
                    data_map[date] = [value]
    # sort the dates (dictionary keys)
    sorted_dates = list(data_map.keys())
    sorted_dates.sort()
    # create a list of dates and a list of values for plotting
    date_list = []
    value_list = []
    # iterate through the sorted dates
    for date_key in sorted_dates:
        # iterate through the ordered attribute values for this date
        for value in data_map[date_key]:
            date_list.append(date_key)
            value_list.append(value)
    # return the list of dates and attribute values
    return date_list, value_list
```

In [3]:

```
# generate a plot from the specified csv file for the specified trait attribute
# returns a numpy matrix of the plot
# @param csv_filename: the csv file to plot
# @param experiment_id: the experiment id to plot
# @param tray_number: the tray number to plot
# @param col_header: the trait attribute id to plot (csv column header)
# returns None if there is an exception
def generate_plot(csv_filename, experiment_id, tray_number, col_header):
    try:
        # set font
        font = {'family': 'normal',
                'weight': 'normal',
                'size': 16}
        matplotlib.rc('font', **font)
        # load the csv file as a list of dictionaries for each row
        row_data = load_csv(csv_filename)
        # get a set of pot ids
        pot_ids = set()
        # iterate through the rows
        for row in row_data:
            if len(row) > 0:
                # set pot id to none by default
                # this is needed when processing wheat images as there is no pot id column in csv file
                pot_id = None
                # if pot id is in this row
                if Trait.POT_ID in row.keys():
                    # update the pot id
                    pot_id = int(row[Trait.POT_ID])
                # add the pot id to the set
                pot_ids.add(pot_id)
        # sort the keys so that they appear in the legend in a sensible order
        sorted_pot_ids = list(pot_ids)
        sorted_pot_ids.sort()
        # create the figure
        fig, axis = plt.subplots(1, 1, figsize=(15, 10), dpi=300)
        # get the earliest date (for any pot)
        dates = []
        # iterate through all of the pots
        for pot_id in sorted_pot_ids:
            # get list of dates and attribute values for current pot id
            if pot_id is not None:
                pot_id = str(pot_id)
            dates_strs, values = get_trait_info(row_data, experiment_id, tray_number, pot_id, col_header)
            # iterate through the dates
            for date_str in dates_strs:
                # get the date as a datetime object. This will also throw exception if date cannot be parsed
                datetime_obj = datetime.strptime(date_str, "%Y-%m-%d")
                # convert datetime object to string (so they can be ordered).
                date = datetime_obj.strftime('%Y-%m-%d')
                # add the date string the list of dates
                dates.append(date)
        # sort the date strings
        dates.sort()
        # get the very first date
        first_date = dates[0]
        # convert to datetime object for days calculations
        first_date_obj = datetime.strptime(first_date, "%Y-%m-%d")
        # iterate through all of the pots (plot each pot as different line)
        for pot_id in sorted_pot_ids:
            # store list of days from 1st date
            days = []
            # get trait information for current pot id
            if pot_id is not None:
                pot_id = str(pot_id)
            dates_strs, values = get_trait_info(row_data, experiment_id, tray_number, pot_id, col_header)
            # iterate through the returned dates
            for date_str in dates_strs:
                # convert date to datetime object (for day calculations)
                datetime_obj = datetime.strptime(date_str, "%Y-%m-%d")
                # determine the number of days this is from the first date
                time_elapsed = datetime_obj - first_date_obj
                # add 1 to the date (so not 0 when the first day), and add to days list
                days.append(time_elapsed.days + 1)
            # determine label for legend (if label is None then don't add a label. this occurs when there is no pot id)
            if pot_id is not None:
                label = "Pot " + str(pot_id)
                # plot the data for this pot
                axis.plot(days, values, ls='-', label=label, marker='o')
            else:
                axis.plot(days, values, ls='-', marker='o')
        # set-up x axis label
        axis.set_xlabel("Days")
        # replace underscores with spaces for y axis label
        y_axis_label = col_header.replace("_", " ")
        # set y-axis label
        axis.set_ylabel(y_axis_label, fontsize=16)
        axis.minorticks_on()
        # set-up legend
        handles, labels = axis.get_legend_handles_labels()
        axis.legend(handles, labels)
        plt.legend()
        # set layout options
        fig.tight_layout()
        # force rendering (so that we can capture the data and convert to numpy matrix)
        fig.canvas.draw()
        # convert to numpy matrix
        data = np.fromstring(fig.canvas.tostring_rgb(), dtype=np.uint8, sep='')
        data = data.reshape(fig.canvas.get_width_height()[::-1] + (3,))
        # clear the figure
        plt.cla()  # Clear axis
        plt.clf()  # Clear figure
        plt.close()  # Close a figure window
        fig = None
        # return the image data
        return data
    except Exception as ex:
        print("Error: Could not generate plot:", ex)
        return None
```

In [6]:

```
# class that stores mapping between csv column ids as enums and text-friendly descriptions
class Trait:

    # enums for csv column names
    IMAGE_NAME = "Img_name"
    EXP_DATE = "EXP_Date"
    EXPERIMENT_REF = "Genotype_Treatment"
    TRAY_NO = "Tray_No"
    POT_ID = "Pot_ID"
    POT_X = "Pot_X"
    POT_Y = "Pot_Y"
    PROJECTED_LEAF_AREA = "Projected_LeafArea(mm^2)"
    LEAF_PERIMETER = "Leaf_perimeter(mm)"
    CANOPY_LENGTH = "Canopy_Length(mm)"
    CANOPY_WIDTH = "Canopy_Width(mm)"
    STOCKINESS = "Stockiness(%)"
    LEAF_STOCKINESS = "Leaf_Stockiness"
    LEAF_CANOPY_SIZE = "Leaf_CanopySize(mm^2)"
    LEAF_COMPACTNESS = "Leaf_Compactness(%)"
    LARGE_LEAF_NO = "Large_Leaf_No"
    LEAF_TOTAL_NO = "Leaf_TotalNo"
    GREENNESS = "Greenness(0-255)"
    PIX2MM_RATIO = "pix2mm2_ratio"

    # mapping between column ids and text-friendly descriptions (e.g. for GUI table columns)
    TRAIT_IDS = {   PROJECTED_LEAF_AREA: "Projected Leaf Area (mm^2)",
                    LEAF_PERIMETER: "Leaf Perimeter (mm)",
                    CANOPY_LENGTH: "Canopy Length (mm)",
                    STOCKINESS: "Stockiness (%)",
                    LEAF_STOCKINESS: "Leaf Stockiness",
                    LEAF_CANOPY_SIZE: "Leaf Canopy Size (mm^2)",
                    LEAF_COMPACTNESS: "Compactness (%)",
                    LEAF_TOTAL_NO: "Total No. Leaves"
    }

    # list of column id enums for attributes that will be plotted when analysing arabidopsis datasets
    ARABIDOPSIS_PLOT_IDS = [PROJECTED_LEAF_AREA, LEAF_PERIMETER, CANOPY_LENGTH, STOCKINESS, LEAF_CANOPY_SIZE, LEAF_COMPACTNESS, LEAF_TOTAL_NO]
    # list of column id enums for attributes that will be plotted when analysing wheat datasets
    WHEAT_PLOT_IDS = [PROJECTED_LEAF_AREA, CANOPY_LENGTH, LEAF_STOCKINESS, LEAF_COMPACTNESS]
    # list of column id enums for attributes that will be plotted when no experimental data available
    NO_EXPERIMENTAL_DATA_PLOT_IDS = []
```
