# Supplementary material for: Leaf-GP: an open and automated software application for measuring growth phenotypes for arabidopsis and wheat
Source: Plant Methods. 2017 Dec 22;13:117. doi: 10.1186/s13007-017-0266-3 (PMC5740932; doi:10.1186/s13007-017-0266-3)
Supplement: Supplementary file 6 — Additional file 6. The analysis workflow and a detailed activity diagram of Leaf-GP. [file 13007_2017_266_MOESM6_ESM.docx]

**Additional File 6: The Analysis Workflow of Leaf-GP**

**Additional Fig. 6.1** The analysis workflow of Leaf-GP.

To explain functions and procedures developed in Leaf-GP, we designed a UML (unified modelling language) activity diagram for elucidating analysis actions (Add. Fig. 6.1). Software engineering activities such as choice, iteration, and concurrency are also presented in the diagram, which are customised for the batch image processing. The analysis workflow contains three phases: data selection, trait analysis, and results output.

#### **Phase 1 – data selection**

This phase (coloured red, Add. Fig. 6.1) is accomplished via the GUI or the command-line interface. After entering input parameters, all qualified images in a directory will be grouped into series. The HPC and Notebook versions can only accept images following the naming convention.

#### **Phase 2 – automated trait analysis**

The second phase (Add. Fig. 6.2) contains a range of computer vision actions to extract meaningful results from selected image series. This phase can be divided into preparing the series (Add. Fig. 6.2.1), carrying out pre-processing (Add. Fig. 6.2.2), and quantifying phenotypes (Add. Fig. 6.2.3):

1. To prepare the image series (coloured light gold), the file extension of each image in the directory is verified. Only JPEG (Joint Photographic Experts Group, i.e. .jpg or .jpeg) and PNG (Portable Network Graphics, i.e. .png) files are accepted. Based on experimental metadata, verified images will be associated with experiments and images without metadata are grouped as an arbitrary series. A result folder and an empty CSV file will be created to contain processing results (e.g. processed images and trait measurements) during the batch processing.

1. To pre-process and calibrate images (coloured light green), this phase rescales gamma and intensity histograms of a given image by a fixed scaling range (i.e. from 25^th^ to 75^th^ percentile of the original value) and then applies noise reduction method to smooth the rescaled image. By completing the above calibration actions, all images can be analysed with the same standard.
2. The trait analysis phase (coloured light green) utilises unsupervised machine-learning (i.e. k-means), Lab colour space, and excessive greenness to produce a global leaf mask at the image level. After that, all pots are reconstructed based on the row and column values entered previously, so that the core algorithm can improve leaf object detection through a local feature selection approach within each pot. When the refined leaf masks are generated, complex trait analysis algorithms are applied to quantify growth phenotypes.

#### Phase 3 - results output

The last phase (coloured light red, Add. Fig. 6.3) exports processed images and a CSV file containing the quantification of leaf growth phenotypes in each pot over time (if a pot has been destructively harvested, all trait analysis in the pot will be set to *zero*). The GUI version of the software also saves processing statistics, for example, how many images have been successfully analysed and how many images have been declined, together with related error or warning messages in a log file for debugging.
