# Supplementary material for: Leaf-GP: an open and automated software application for measuring growth phenotypes for arabidopsis and wheat
Source: Plant Methods. 2017 Dec 22;13:117. doi: 10.1186/s13007-017-0266-3 (PMC5740932; doi:10.1186/s13007-017-0266-3)
Supplement: Supplementary file 7 — Additional file 7 The manual for importing image datasets via the GUI version of Leaf-GP. [file 13007_2017_266_MOESM7_ESM.docx]

**Additional File 7: Import Image Series Using Leaf-GP GUI**

**Scenario 1: Selecting “From Image Name”**

- Read experiment data (i.e. metadata) from image names.
- The naming convention of images is: “*YYYY*-*MM*-*DD*_*Experiment*_*Tray-Number*”.
- All image files followed the naming convention in the selected directory are imported, subdirectories and images that are *not* following the naming convention will not be imported (**Additional Figure 7.1**).
- Imported images will be ordered by the date specified in the filename (YYYY-MM-DD) before batch processing.
- LeafAP will group imported image files into different image series based on metadata contained in filenames, for example, different experiments (e.g. “Ler-12C”) and then different tray numbers.

Selected Image Directory


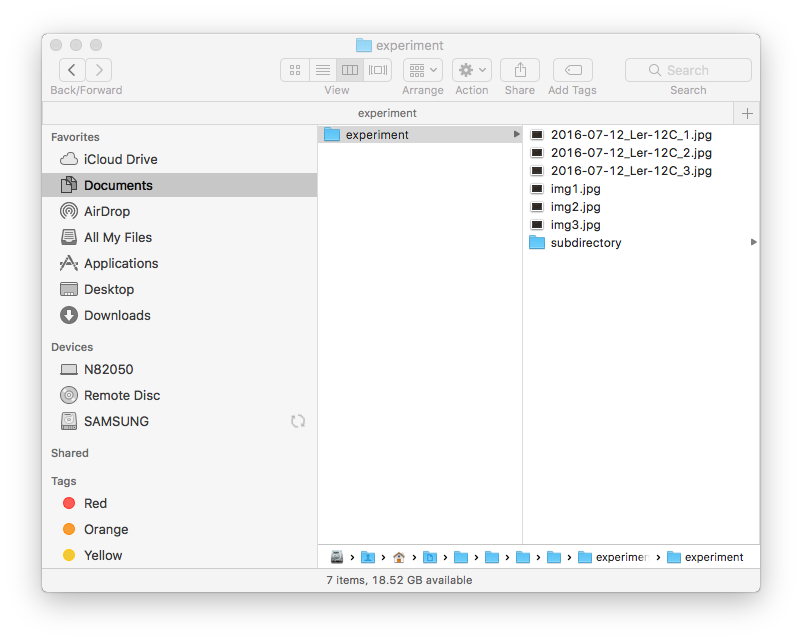


Imported

Not Imported

**Additional Figure 7.1 Scenario 1 – read metadata from image filenames**

**Scenario 2: Selecting “From Folder Name”**

- Read experiment data from folder names.
- The naming convention of a folder name is: “*Experiment* _*Tray-Number*”.
- Level one subdirectories of the selected directory are imported.
- All image files in the selected directory are not imported as they do not belong to a specified folder series.
- Folder(s) that do not follow the naming convention are not imported (**Additional Figure 7.2**).
- All image files within the imported level-one subdirectories are grouped and treated as an image series (**Additional Figure 7.2**).
- Imported images will be ordered based on the modification dates recorded on the operating system.

Selected Image Directory


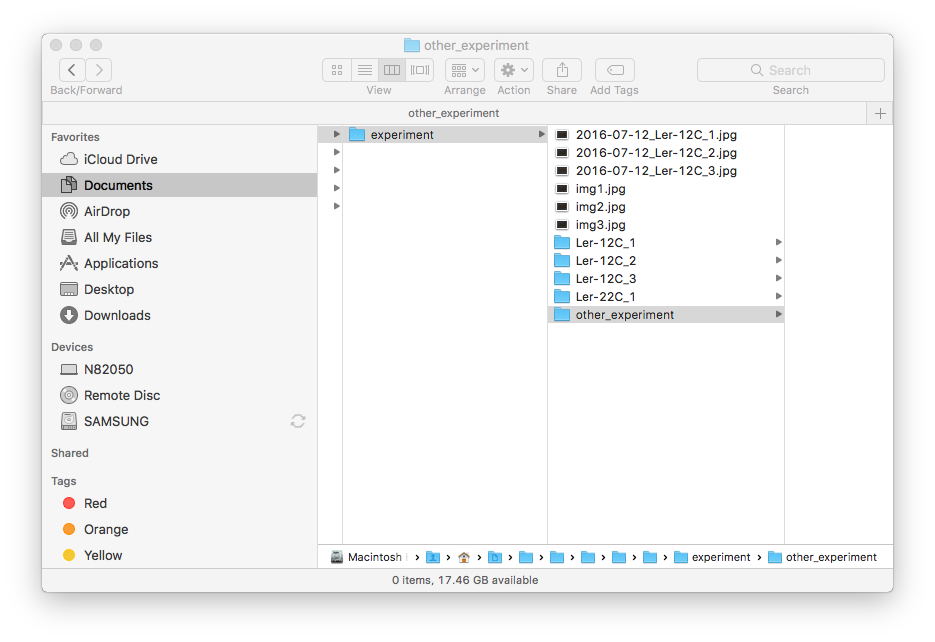


Imported
(each folder as separate series)

Not Imported

Not Imported

**Additional Figure 7.2 Scenario 2 – read metadata from folder names**

**Scenario 3: Selecting “No Experimental Data Available”**

- No metadata is provided.
- All image files in selected folder are imported.
- No subdirectories are imported.
- All images are treated as one image series – the trait plot function in the result section of LeafAP will be *disabled*.


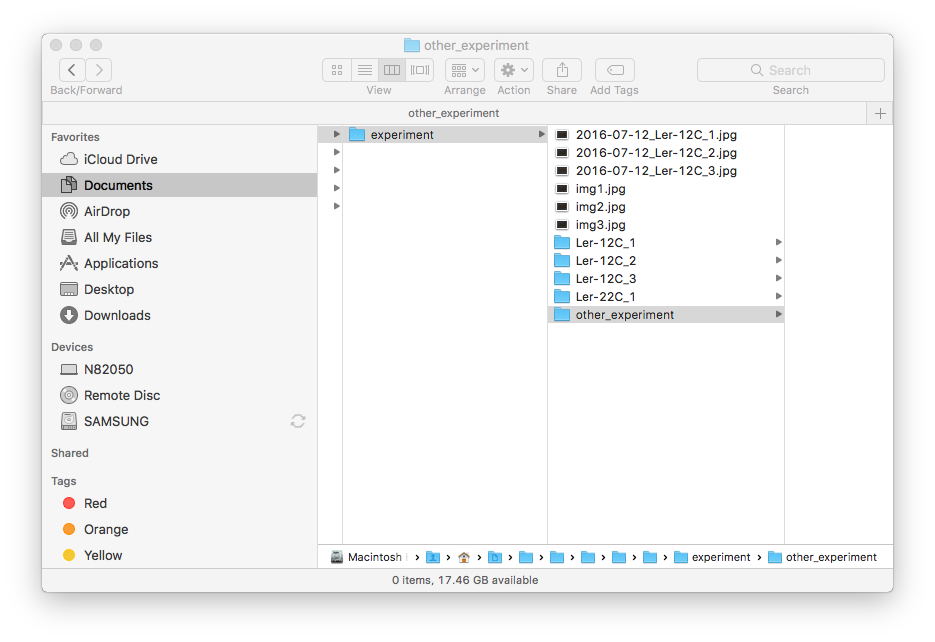


Imported
(each file as separate series)

Not Imported

Selected Image Directory

**Additional Figure 7.3 Scenario 3 – no metadata is provided**
